# Additives Promote Divergent Reactivity in Photolytic Deoxygenation and Lactonization Reactions of Cobalt Alkoxycarbonyls

**DOI:** 10.1002/ejoc.202501091

**Published:** 2026-03-07

**Authors:** Jacob N. Hackbarth, Dana R. Chambers, Cory T. Ludwig, Tyler K. Brown, David B. C. Martin

**Affiliations:** 1 Department of Chemistry, University of Iowa, Iowa City, Iowa, USA; 2 Department of Chemistry, University of California, Riverside, California, USA; 3 Contineum Therapeutics, San Diego, California, USA

**Keywords:** alkoxycarbonyl radical, cobalt, lactonization, ligand effects, photolysis

## Abstract

The formation of lactones via radical-mediated C─C bond formation is a powerful but overlooked method, especially given recent interest in the conversion of alcohols to radical intermediates using photochemistry. Building on our previous work with cobalt porphyrin complexes, a carbonylation-homolysis process is described for complex unsaturated alcohols and a simple benzylic model system, leading to the production of saturated or unsaturated products. Ligands and additives are demonstrated to promote either reductive or oxidative termination, leading to divergent reactivity in different contexts. The role of cobalt hydride intermediates is also discussed, as well as the application of this strategy toward the synthesis of a lactone-containing limonoid natural product.

## 1 | Introduction

**SCHEME 1 |:**
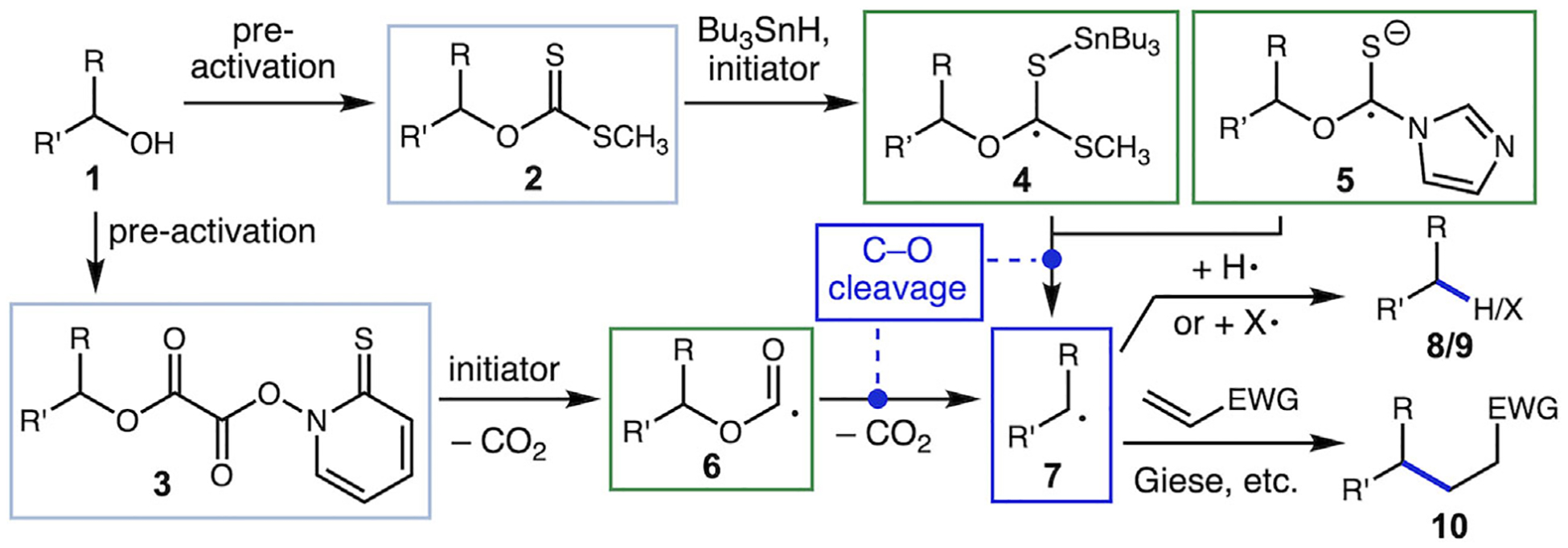
Barton–McCombie deoxygenation and related methods for alcohol activation.

Alcohols are among the most abundant functional groups in both natural products and commercially available chemical feed-stocks, making step-economical transformations of these moieties of particular interest in the synthetic community. In the realm of radical chemistry, a historically important contribution was the development of the Barton–McCombie deoxygenation, which uses xanthate esters **2** as activated intermediates to enable reductive cleavage of the C─O bond upon reaction with tributyltin hydride and a radical initiator (Scheme 1, **2** → **8**) [1, 2]. Many variations of this chemistry have been developed, such as alternative activation groups (e.g., mixed oxalate ester **3**) [3] and trapping of the C-centered radical with other reagents, allowing for the formation of C─C, C─S and other C─X bonds (products **9** and **10**) [4–7]. More recent advances have included the development of photoredox strategies for the reductive generation of species such as **5** for deoxygenation by Ollivier and oxidative fragmentation of alcohol-NHC adducts (not shown) for deoxygenative coupling by MacMillan [8–10]. A key feature of these strategies is the weakening of the C─O bond in intermediates such as **4**–**6**, allowing for ß-scission. Furthermore, formation of even unstabilized radicals **7** is driven by the formation of a stable carbonyl-containing byproduct (thiocarbonate derivative or CO_2_, etc.).

**SCHEME 2 |:**
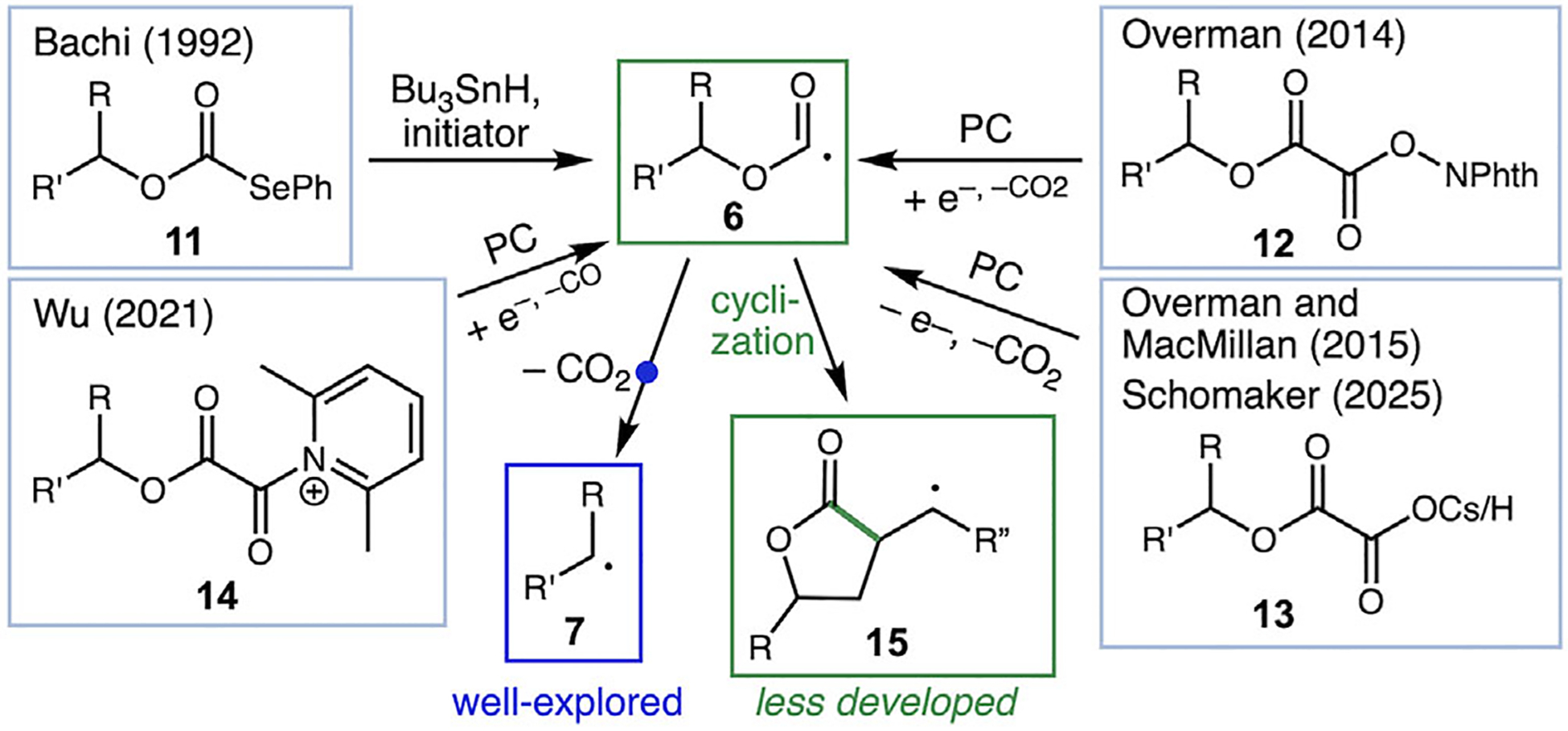
Methods to access alkoxycarbonyl radicals, including lactonization by Bachi and recent photoredox methods.

Recently, a number of reactions have been developed that take advantage of oxalate esters related to **3** for radical generation from alcohols, including contributions by Overman, MacMillan, Wu, Schomaker, and others (**12**–**14**, Scheme 2) [11–14]. While the main focus has generally been the double decarboxylation of the oxalate group by C–O ß-scission to form radical **7**, the decarboxylation of acyl radical intermediate **6** is known to be slower and substrate-dependent [15]. Thus, depending on the substrate and reaction conditions, it is possible to intercept the alkoxycarbonyl radical **6** to provide products such as esters and lactones (**15**) that retain the carboxyl group [11, 14, 16]. Historically, selenocarbonates such as **11** have been used as activated intermediates for alkoxycarbonyl radical generation, producing undesirable selenium- and tin-containing byproducts [17]. Despite the attractiveness of generating lactones via C─C bond formation in this manner, this strategy remains much less developed and overlooked as a tool for lactone formation.

**SCHEME 3 |:**
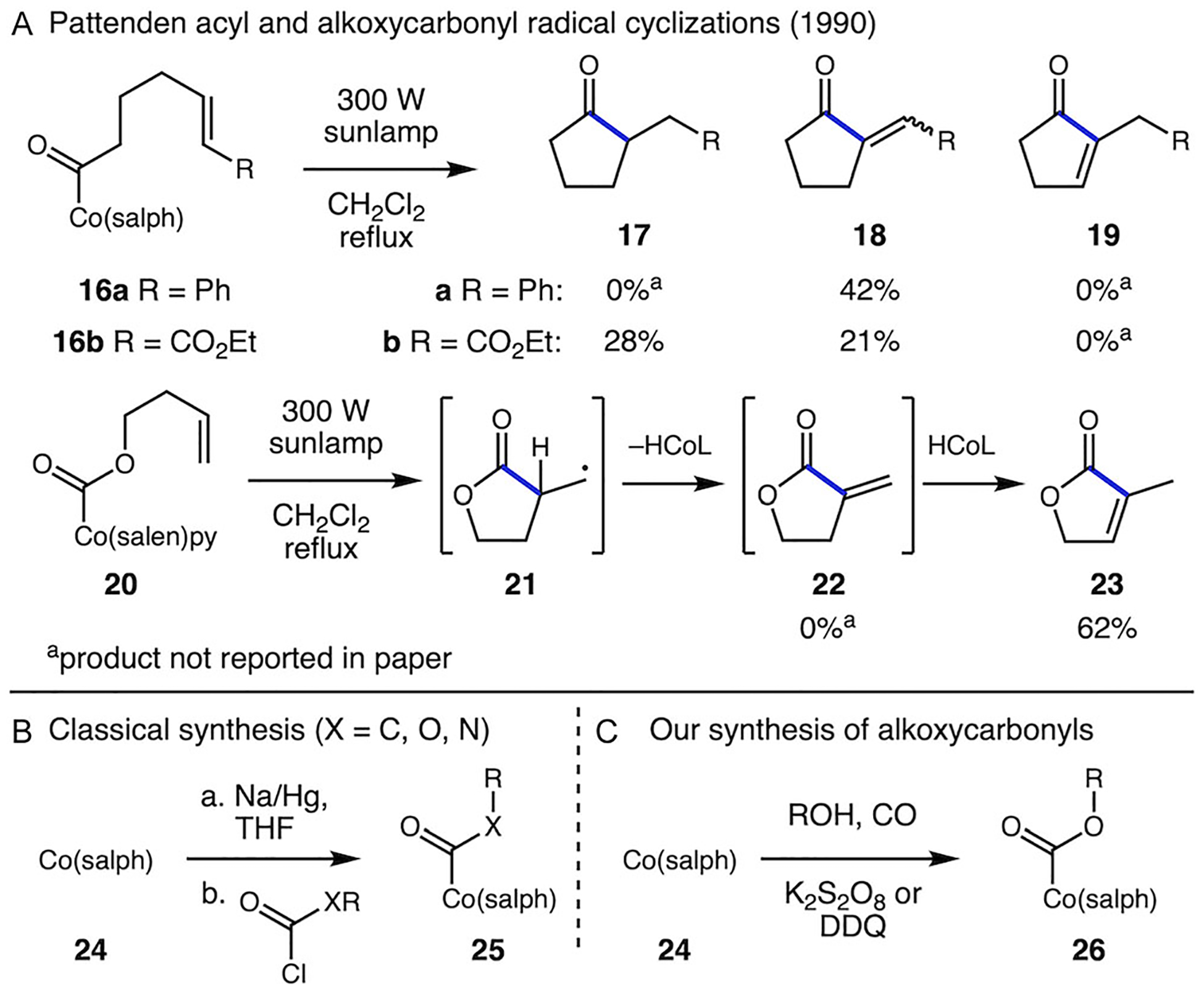
Use of acyl cobalt complexes and synthesis methods to access them.

A unique approach to the generation of acyl radicals was reported by Pattenden and coworkers via homolysis of isolable cobalt(III) acyl species (Scheme 3) [18]. As part of their pioneering studies on the synthetic utility of organocobalt species as radical precursors, Pattenden reported the homolysis of a range of acyl cobalt complexes to give ketone products with either inter or intramolecular C─C bond formation (Scheme 3A); the ratio of saturated and unsaturated ketones depended on the substrate (product **18a** vs. **17b/18b**) or the addition of reagents [19]. A limited number of examples of alkoxycarbonyl cobalt species were irradiated to give ester products, and one example of cyclization to give a butenolide product **23** was reported [20]. These acyl and alkoxycarbonyl cobalt species are synthesized through the reduction of Co(II) with Na/Hg amalgam to form the supernucleophile Co(I), which reacts with acid chlorides and chloroformates to form the acylated Co(III) complexes (Scheme 3B) [18]. While this work is a key inspiration for our studies of alkoxycarbonyl cobalt species as radical precursors, more detailed investigations of the lactone-forming reaction and the influence of ligands on reaction outcome have not been reported.

**SCHEME 4 |:**
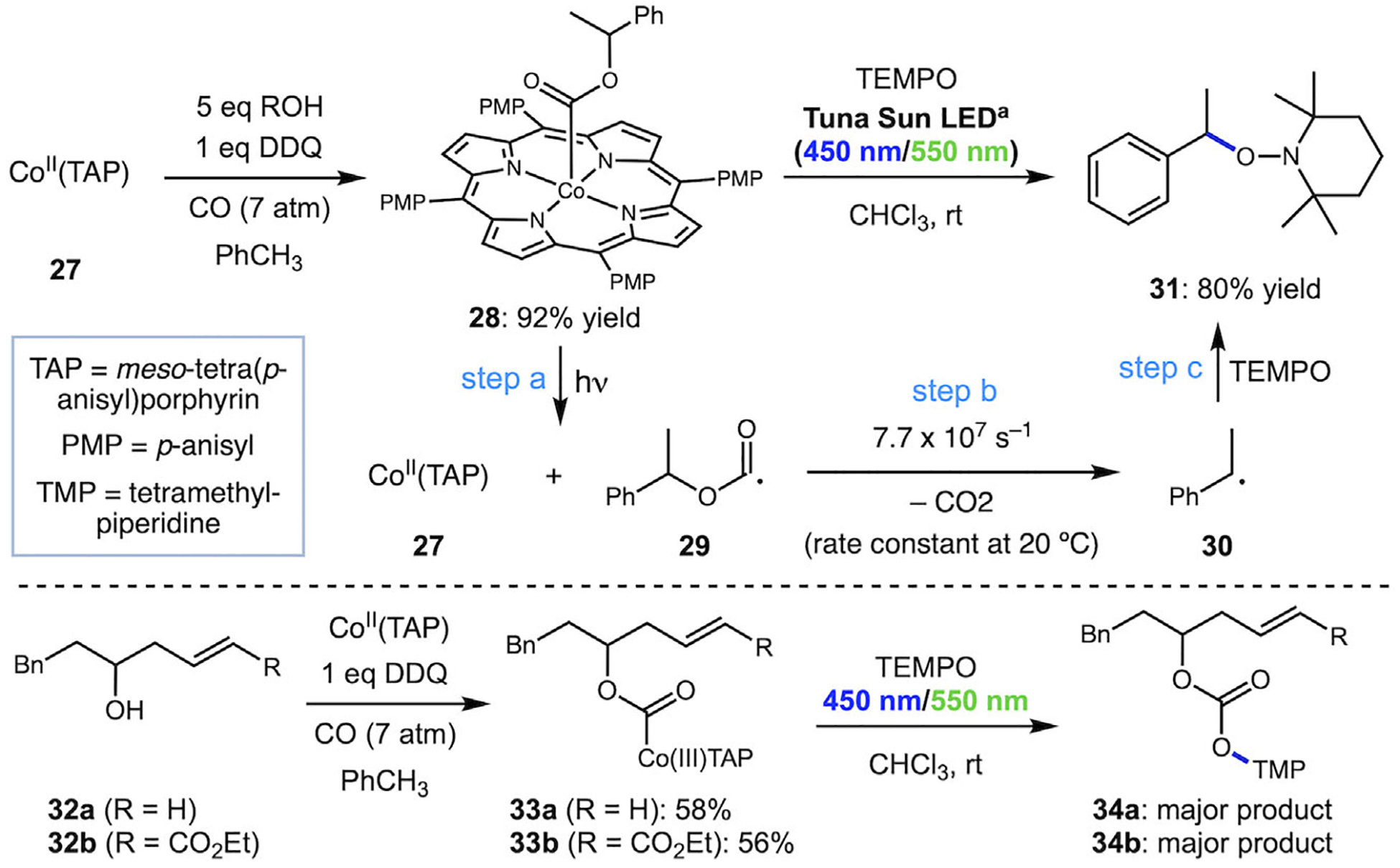
Carbonylation of alcohols and photolysis in the presence of TEMPO. ^a^See Supporting Information for the emission spectrum of Kessil Tuna Sun LED.

Our laboratory has previously investigated the use of alkoxycarbonyl cobalt species as an alternative strategy for the activation of alcohol C─O bonds via decarboxylation of alkoxycarbonyl radicals [21, 22]. We developed carbonylation reactions of cobalt salen/salophen and porphyrin complexes as a general and more streamlined approach to alkoxycarbonyl cobalt products directly from alcohols (Scheme 3C). The weak Co─C bond of species like **25/26** (calculated to be ~40 kcal/mol for a Co(III)porphyrin derivative **28**, Scheme 4) is readily cleaved with visible light to give alkoxycarbonyl radicals, which we confirmed can undergo decarboxylation and trapping [22]. Inspired by Pattenden’s extensive work on C─C bond-forming reactions and the potential for a cobalt-based approach to provide a more direct alternative to the methods described above, especially if rendered catalytic, we set out to redirect these radical intermediates toward a cyclization process to give lactone products. Success in this approach would require a more complete understanding of three main features of the mechanism: 1) the competition between decarboxylation and cyclization of the alkoxycarbonyl radical, 2) the fate of the radical in the presence of ligated Co(II) byproducts from homolysis, and 3) the role of cobalt hydrides. Herein, we report our findings on cobalt-mediated deoxygenation and lactonization, as well as our application of this strategy toward the synthesis of a lactone-containing limonoid natural product.

## 2 | Results and Discussion

### 2.1 | Benzylic Alcohol Deoxygenation Studies

We began our studies with a series of experiments using a benzylic alcohol substrate to investigate the fate of simpler radicals under relevant reaction conditions. Our mechanistic hypothesis begins with the photolytic homolysis of alkoxycarbonyl cobalt (III) tetra-anisyl porphyrin (TAP) complexes, generating alkoxycarbonyl radicals (step a, Scheme 4). These intermediates can undergo decarboxylation (step b) or engage in C─C bond-forming reactions (see Scheme 2), with the outcome governed by the relative rates of several competing pathways. Newcomb and coworkers measured rate constants for decarboxylation and reported that benzylic systems decarboxylate rapidly (7.7 × 10^7^ s^–1^ for **29**) due to the stability of the resulting carbon-centered radical **30**, while non-benzylic species decarboxylate much slower (~5 × 10^2^ s^–1^) [15]. We used our oxidative carbonylation method to convert 1-phenylethanol to substrate **28** in 92% yield [22]. Irradiation of **28** with visible light in the presence of TEMPO led to the exclusive formation of product **31** in 80% yield, and other benzylic alcohol substrates gave similar results, as reported previously [22]. For comparison, irradiation of nonbenzylic substrates **33a** and **33b** in the presence of TEMPO led to isolation of products **34a** and **34b** (~65% yield for both) that retain the CO_2_ group (confirmed by ^13^C nuclear magnetic resonance (NMR) and high-resolution mass spectrometry (HRMS)). These results are consistent with the slower rate of decarboxylation for unstabilized radical generation, and mirror results from Pattenden with the related Co(salph)-derived species (Scheme 3) [20].

**SCHEME 5 |:**
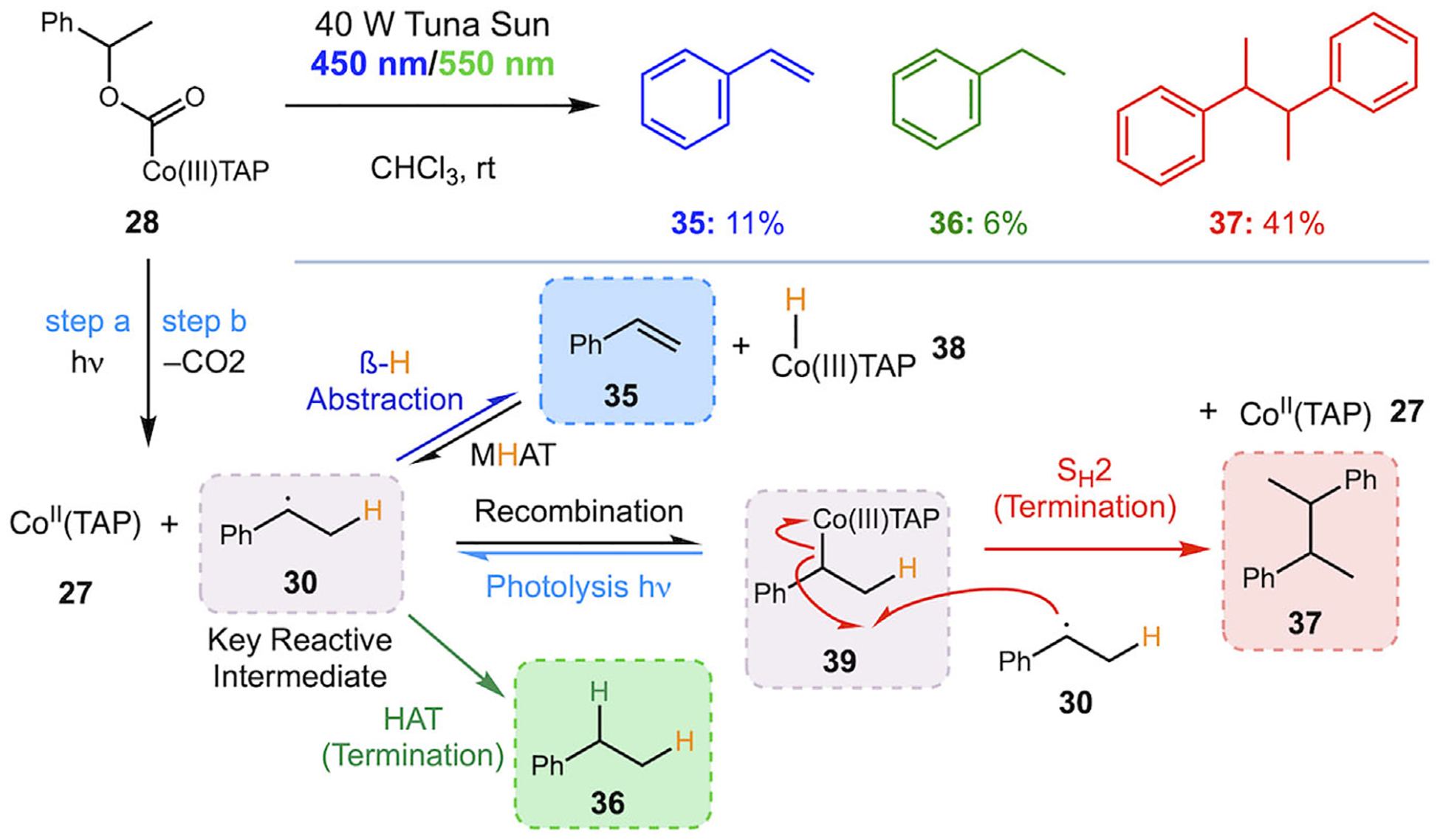
Additive-free photolysis of complex **28** with proposed mechanism.

With evidence for radical generation in hand, we began to investigate the homolysis reaction with and without different additives. When substrate **28** was subjected to the standard homolysis conditions without additives, we found that the major product was consistently the homodimer **37** (41%, Scheme 5). Smaller amounts of styrene and ethylbenzene were also produced (11% and 6%, respectively).

A mechanism to explain the formation of all three products was put forward (Scheme 5, bottom). Styrene presumably results from *β*-hydrogen abstraction from alkyl radical **30** by Co(II) to give Co(III)-H **38** as a byproduct, while ethylbenzene **36** results from H-atom transfer (HAT) from a source like Co(III)-H **38**. Pathways leading to styrene or ethylbenzene without the involvement of Co(II) are unlikely due to the slow rate of H-atom transfer from carbon to carbon [23]. We have not directly observed the proposed Co(III)-H **38**. Numerous efforts to observe Co(III)-H species with related ligand systems in the literature have failed [24], although cobalt hydrides are key intermediates proposed in many processes, including alkene hydrofunctionalization and hydrogen evolution [25–27]. The formation of the homodimer product **37** could, in principle, result from direct dimerization of the benzylic radical intermediate **30**, but its formation is better explained by an S_H_2 reaction between the benzylic radical **30** and the alkyl cobalt complex **39** [28–30]. Alkyl complex **39** [25] is a key intermediate in the Co(II)TAP-catalyzed radical polymerization of styrene [31, 32] and should be readily formed by recombination of the benzylic radical and Co(II)TAP byproduct generated from the homolysis. This process is a key step in recent “radical sorting” methods reported by MacMillan and coworkers [29, 30]. Once formed, complex **39** would be in equilibrium with the benzylic radical, which could undergo S_H_2 with each other in solution. Such reactivity is well known for Co(III) alkyl complexes and is analogous to the pathways found in vitamin B12 biocatalysis [29]. Formation of alkyl cobalt species, at least reversibly, is especially likely due to the stoichiometric amount of Co(II)TAP produced as a byproduct and the reported isolation of species like **39** by Gridnev, Halpern, and others [18, 25, 33].

Next, we examined the influence of additives and ligands in order to intercept the benzylic radical **30** to selectively form ethylbenzene (**36**). The addition of most H-atom donors led to the exclusive formation of ethylbenzene (Table 1, entries 2–7), with sterically hindered thiols providing the highest yield (entries 2–5, up to 94% yield) [22]. However, over the course of the reactions in entries 2 and 3, it was apparent that significant amounts of the corresponding disulfide were forming, potentially consuming the thiol before it could reduce the radical intermediate. In a control experiment, we independently showed that thiols are efficiently converted to disulfides in the presence of Co(II)TAP, even in a catalytic amount. This may be attributable to trace oxygen based on a prior report of such reactivity [34], although reactions were run with rigorous removal of air. To mitigate this issue, Hantzsch ester (HE) was included to reduce thiyl radical byproducts and prevent the rapid formation of disulfide under the reaction conditions (entries 4–5). Hantzsch ester alone was not effective, presumably due to the slow rate of H-atom transfer from carbon to carbon [22, 23, 35]. Tributyltin hydride and supersilane eliminated the formation of styrene but gave lower yields of ethylbenzene (54% and 15%, respectively).

In addition to thiols, we tested hydroquinones as electrophilic H-atom sources [36]. In this exploration, we were surprised to discover that when trimethylhydroquinone (Me_3_HQ) was used as the additive, styrene (**36**) became the major product (Table 1, entry 8). This product presumably increases by favoring *β*-hydrogen abstraction by Co(II) to give Co(III)-H **38**, similar to catalytic chain transfer in radical polymerization [25, 31, 32]. The precise role of the hydroquinone in driving this reaction forward or suppressing the reverse reaction metal hydrogen atom transfer (MHAT) is not obvious. It could involve hydrogen gas evolution [37] with the Co(III)-H species or H-atom shuttling [38] via the semiquinone. Control experiments indicate that hydroquinone Me_3_HQ is oxidized to the corresponding quinone in the presence of Co(II)TAP, accompanied by precipitation of the cobalt catalyst from solution and formation of detectable amounts of H_2_ gas in the headspace. These observations suggest that hydroquinone reacts with Co(III)-H species and effectively sequesters cobalt, thereby preventing the formation of the alkyl cobalt complex via MHAT addition of Co(III)-H into styrene to yield the alkyl cobalt intermediate **39** thereby allowing styrene to exist as the major product (see below for discussion of MHAT).

In parallel, we investigated the addition of potential axial ligands (amines and phosphines) to tune the reactivity of the cobalt species (e.g. favorability of ß-hydrogen abstraction, the relative hydricity of Co(III)-H intermediate, etc.) [39, 40]. PPh_3_ gave a modest improvement in the yield of ethylbenzene to give the optimal result in entry 4 (86%). Conversely, among the ligands tested, benzimidazole led to complete suppression of ethylbenzene formation, and styrene became the major product in moderate yield. When both benzimidazole and hydroquinone were used in combination, styrene was produced in a 91% yield with complete selectivity (Table 1, entry 10). The additive effect of benzimidazole and hydroquinone suggests they may play complementary roles, with benzimidazole serving as a pi-withdrawing ligand and the corresponding semiquinone or quinone serving to favor H_2_ evolution.

With these results in hand, we sought to apply these additive effects to the lactonization reactions of alkene-tethered substrates with the goal of tuning for saturated or unsaturated products selectively.

### 2.2 | Unsaturated Alcohol Lactonization Studies

**SCHEME 6 |:**
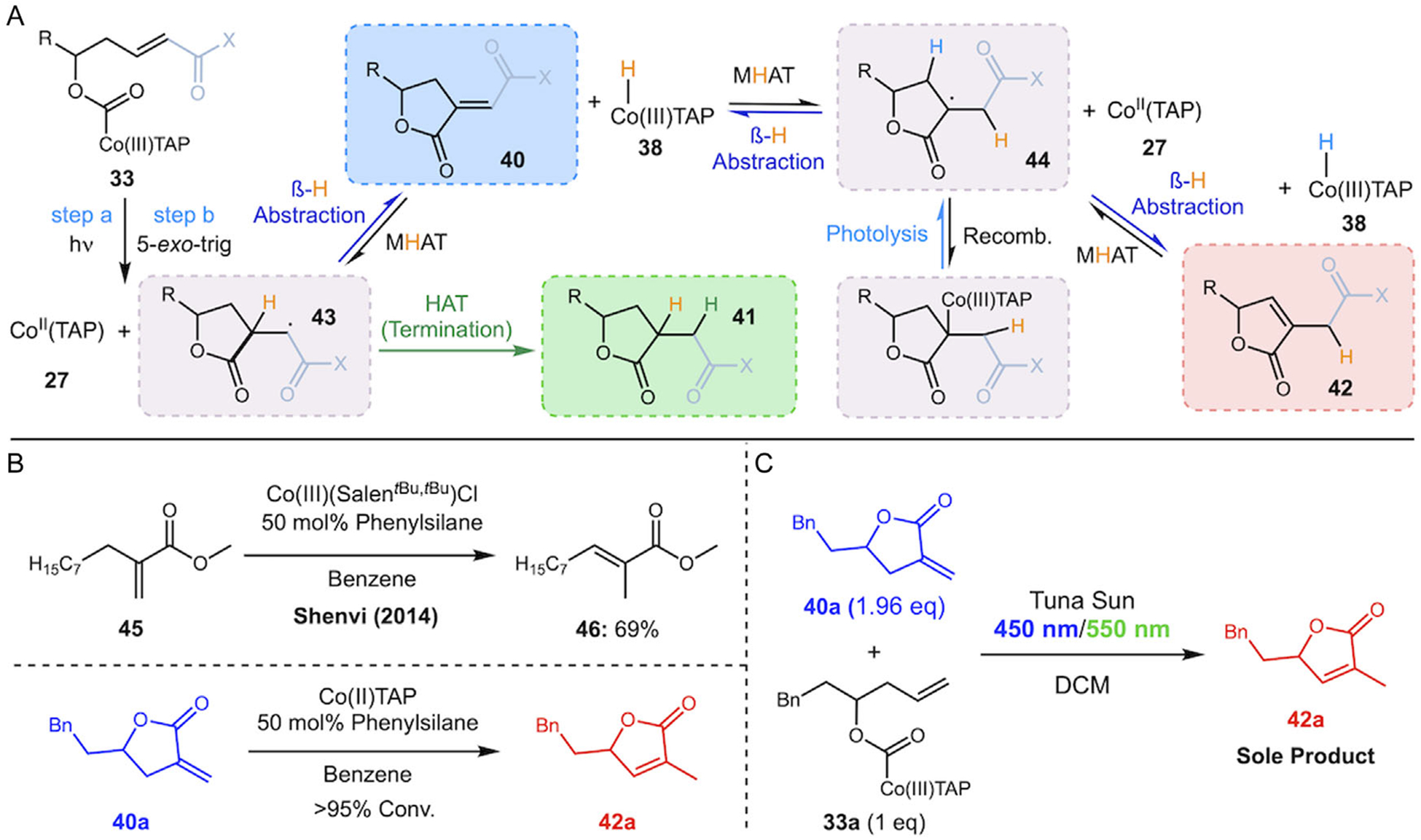
(A) Mechanism of lactone formation with isomerization pathways. (B) Control reaction shows evidence of isomerization promoted by cobalt hydride as reported by Shenvi and coworkers. (C) Isomerization of exocyclic alkene **40a** under the standard reaction conditions.

We next turned our attention to the lactonization reaction with terminal alkene **33a**, *α*,ß-unsaturated ester **33b**, and *α*,ß-unsaturated ketone **33c** (see Scheme 4 and SI for their synthesis). Under additive-free conditions, irradiation of both substrates **33a** and **33b** led to efficient cyclization; however, both reactions yielded exclusively isomerized products **42a** and **42b** with an endocyclic alkene (Table 2, entries 1 and 2). Enone substrate **33c** also led exclusively to the endocyclic product **43c** (entry 3). We observed no evidence of reduced products **41a**–**c** under these conditions, and under no conditions did we observe products resulting from dimerization or decarboxylation of the radical intermediates as significant products. Our initial expectation was that after 5-*exo* cyclization, ß-hydrogen atom abstraction by Co(II)TAP would lead to the exocyclic alkenes **40a**–**c** and the cobalt hydride Co(III)TAP-H (**38**). It appears that under the majority of conditions tested, exocyclic alkene products undergo rapid MHAT isomerization to the more stable endocyclic alkene [26, 27] with the highest yields and selectivity of the isomerized product coming from the additive-free conditions. Control experiments supported this interpretation: when terminal exocyclic alkene **40a** was subjected to Shenvi’s MHAT conditions (phenylsilane, benzene, rt) [26] but with our Co(II)TAP used as the isomerization catalyst, only the isomerized product **42a** was observed (see Scheme 6B). Furthermore, when 2 equivalents of terminal exocyclic alkene **40a** were added at the start of the lactonization reaction using **33a** under standard conditions, the isomerized isomer **42a** was again the sole product (see Scheme 6C) measured via NMR assay to be equal to 3 eq of isomerized product, meaning that the conversion for both isomerization and lactonization from **33a** was close to 100%. These results indicate the exocyclic alkene is a viable intermediate and would be expected to undergo isomerization under these conditions, and this process is likely mediated by an H–Co(III)TAP species formed during the reaction and echoes the substrate-dependent isomerization reported by Pattenden (Scheme 3A) [19, 20].

These results led us to consider the more complex potential mechanistic scenario for product divergence presented in Scheme 6A and investigate further. After homolysis and rapid 5-*exo*-trig cyclization [15], the carbon-centered radical **43** may follow several pathways: it can react with hydrogen atom sources to yield reduced products (**41**), or undergo *β*-hydrogen abstraction to form exocyclic alkenes (**40**) and Co(III)–H. The hydride species can then participate in MHAT isomerization, converting **40** to the thermodynamically favored **42** as depicted in Scheme 6A. In the context of the more complex mechanistic landscape, we applied the conditions described in the simple model system to see if these additives could favor reduced or unsaturated products in a similar manner. For both lactonization substrates **33a** and **33b**, the results were markedly different (Table 2, entries 4–7). For *α*,ß-unsaturated ester **33b**, neither the thiol additive nor the hydroquinone/benzimidazole conditions were able to shift the major pathway away from the isomerized product **42b** or produce any detectable exocyclic alkene **40b**. Rather, the only discernible secondary product for both of these conditions was the reduced product **41a/b**, which was especially surprising considering the hydroquinone/benzimidazole conditions had completely eliminated the formation of ethylbenzene as reported above. In contrast, only terminal alkene **33a** responded to hydroquinone/benzimidazole (i.e., styrene-favoring) conditions, yielding nonisomerized **40a** as the major product in moderate yield (Table 2, entry 6), albeit with poor selectivity (all three products are produced).

**SCHEME 7 |:**
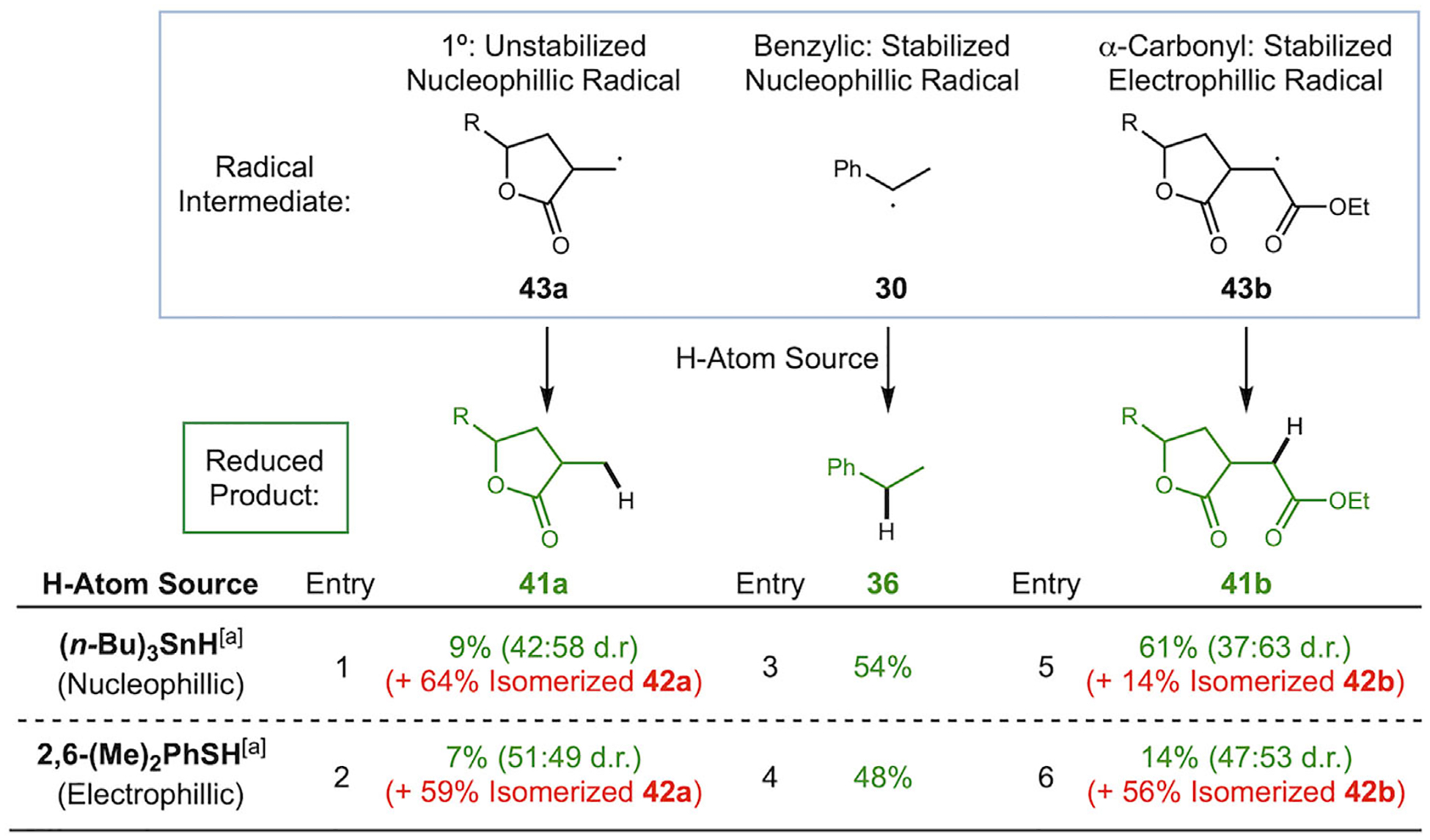
Photolysis results in the presence of nucleophilic and electrophilic H-atom sources. Conditions: 2 eq H-atom source, CH_2_Cl_2_ (entries 1, 2, 5, 6) or CHCl_3_ (entries 3 and 4). ^a^Reported X–H BDEs: 78–79 kcal/mol for Bu_3_SnH, 79–80 kcal/mol for PhSH and MesSH [42, 43].

To explain this divergence in reactivity with thiol compared to the model system (Table 1), we hypothesized a complex interplay between polarity mismatch and radical stability between the radical formed after lactonization and the H-atom source [41]. The initially formed radicals after 5-*exo* cyclization and decarboxylation (see Scheme 7) are either nucleophilic (**43a** and **30**) or electrophilic (**43b**), and a polarity-matched partner is required for fast and efficient reactivity, as described by the seminal studies of Roberts [35]. Testing both nucleophilic and electrophilic H-atom donors revealed that for nonstabilized radicals like primary radical **43a**, *β*-hydrogen abstraction appears to be rapid compared to the reduction, forming **40a** and Co(III)–H, which then quickly isomerizes to endocyclic **42a**. This explains the dominance of the isomerized product **42a** regardless of H-atom source philicity (Scheme 7, entries 1 and 2).

In contrast, stabilized radicals such as benzylic **30** and *α*-carbonyl radical **43b** persist long enough before ß-hydrogen atom abstraction to be reduced by a suitable H-atom donor. The benzylic substrate proceeding via radical **30** gave similar yields with both nucleophilic tin hydride and electrophilic thiol (Scheme 7, entry 3: 54%, entry 4: 48%). In contrast, polarity matching appears crucial for electrophilic radicals like *α*-carbonyl radical **43b**, which gave poor yields of reduced product **41b** when electrophilic thiophenol was used (entry 6, 14%), but an improved yield with nucleophilic tributyltin hydride (entry 5, 61%), despite essentially identical X–H bond dissociation energies (BDEs) for the two donors [41–43]. These findings support the hypothesis that radical philicity is a key determinant of product outcomes and that the intermediates responsible for selectivity divergence are those formed directly after lactonization (**43a** vs **43b**), rather than those formed under homolytic equilibrium following MHAT addition (i.e., **44**, *α*-carbonyl for both **44a** and **44b**).

While the lactonization system was not as amenable to tuning product selectivity with simple additives as the deoxygenation system, the additive-free lactonization provided consistently high yields and led us to consider applications toward specific lactone targets. With an appropriate substrate, we could compare the relative rates of decarboxylation and lactonization in the synthesis of limonoid natural products, where the lactonization was expected to be accelerated by the particular features of the substrate.

### 2.3 | Application to Limonoid Natural Products

**SCHEME 8 |:**
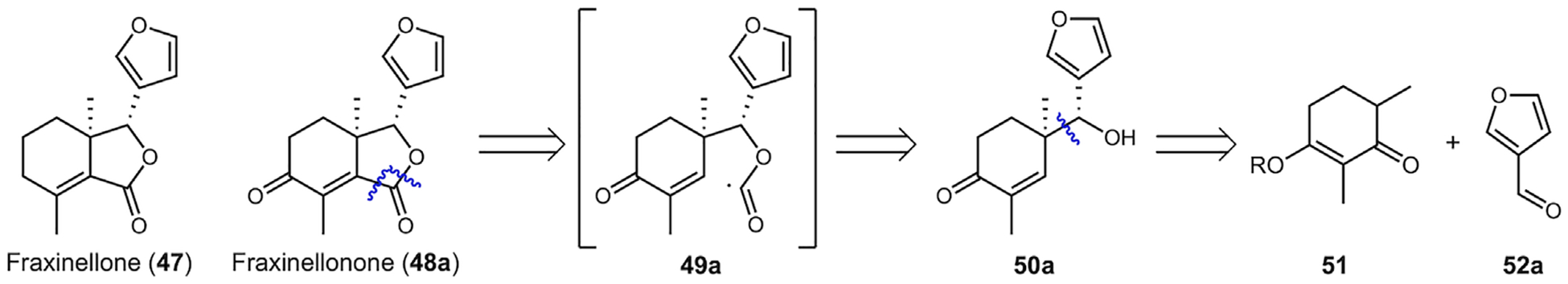
Retrosynthesis of limonoid natural products via lactonization.

Lactones are common in bioactive natural products, and the limonoids attracted our attention due to their significant neuroprotective properties [44–46]. In our recent work, we synthesized fraxinellone (**47**, Scheme 8) and simplified derivatives, identifying analogs that protect cells from both endogenous (e.g., glutamate) and exogenous (e.g., rotenone) neurotoxicants, offering a promising therapeutic opportunity for oxidative stress-related neurodegenerative conditions [47]. Recognizing the furanolactone moiety in the limonoids, we sought to apply our cobalt-mediated radical chemistry to the synthesis of limonoid scaffolds, offering a mechanistically rich platform to probe the lactonization reaction further [48].

The synthesis of fraxinellonone (**48a**) would require a cobalt-mediated carbonylation/lactonization of pseudo-benzylic furyl alcohol **50a** (Scheme 8). We were cognizant that decarboxylation of intermediate **49a** would present a competing pathway due to the radical stabilization provided by the furan. However, the highly substituted centers between the alkoxycarbonyl radical and alkene (Thorpe–Ingold effect) and activating influence of the conjugated ketone would potentially accelerate ring closure and enable lactonization to outcompete ß-C–O scission.

**SCHEME 9 |:**
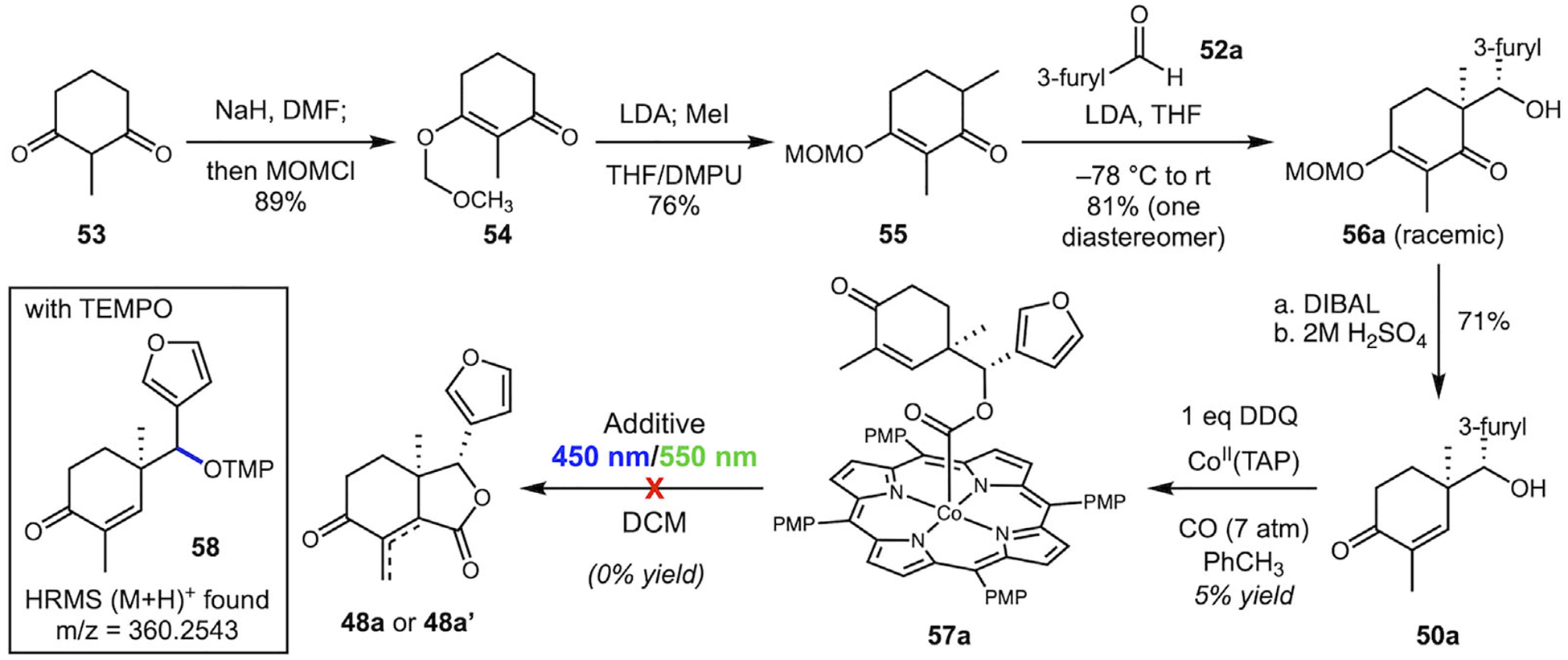
Synthesis of 3-furyl substrate for lactonization studies.

The synthesis of the key intermediate **50** began with 1,3-diketone **53**, which was transformed into MOM-protected vinylogous ester **54**, followed by *α*-methylation to give the common precursor **55** (Scheme 9). The enolate derived from **55** was subjected to an aldol reaction with 3-furaldehyde (**52a**) to give aldol product **56a** with high diastereoselectivity [49]. DIBAL reduction of the carbonyl and careful acidic workup led to cyclohexenone **50a** in good yield via an atypical Stork–Danheiser transposition[50]. The use of the MOM-ester rather than a more typical alkyl ester led to faster hydrolysis and less degradation of the secondary alcohol present in the substrate under acidic conditions [51].

The carbonylation of **50a** to form **57a** proceeded in low yield and proved difficult to purify. It was crucial to remove the alcohol from the product for the next step, and the product readily decomposed on various treated silica gel columns and regular basic alumina, as well as on preparative silica TLC plates. Of all purification methods attempted, only the use of neutral alumina preparative TLC would consistently separate the product from both the alcohol and Co(II)TAP starting material, which was expensive and inefficient (consistently below 5% yield). This gave us only a small amount of product to experiment with. When **57a** was subjected to standard lactonization conditions, the reaction produced a complex mixture without clear evidence of the desired product **48a/48a**’ via NMR or HRMS. When the photolysis was carried out in the presence of TEMPO, we observed the decarboxylation adduct **58** by HRMS, indicating that fragmentation was competing with ring closure to a significant degree. The fate of the majority of the material in both experiments was not clear by NMR or HRMS analysis. The difficulty of obtaining this material caused us to shift our focus to other models that would help us explore this system.

**SCHEME 10 |:**
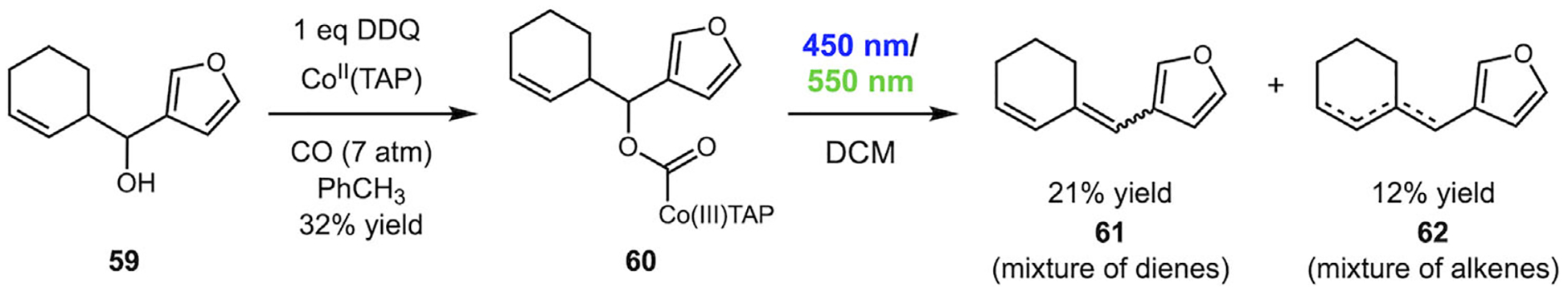
Observation of decarboxylation products with a model substrate **60**.

To further investigate, we examined the less sterically hindered alcohol **59** bearing a lesselectronically withdrawn alkene, subjecting it to the same sequence (Scheme 10). The carbonylation product **60** was isolated in 32% yield, significantly higher than **57a** but still lower than the lactonization models examined in the previous section (Scheme 4, 56%–58%). We attribute this improvement to the reduced steric hindrance of alcohol **59**; however, we cannot rule out some influence of the electronic differences in the alkene. Cobalt(III) alkoxycarbonyl species **60** was subjected to lactonization conditions, yielding a mixture of decarboxylated alkene isomers (diene **61** and alkenes **62**) in a combined yield of 33%, supported by GCMS and ^1^H NMR data, strengthening the proposal that decarboxylation outcompetes lactonization in furyl alcohol systems.

**SCHEME 11 |:**
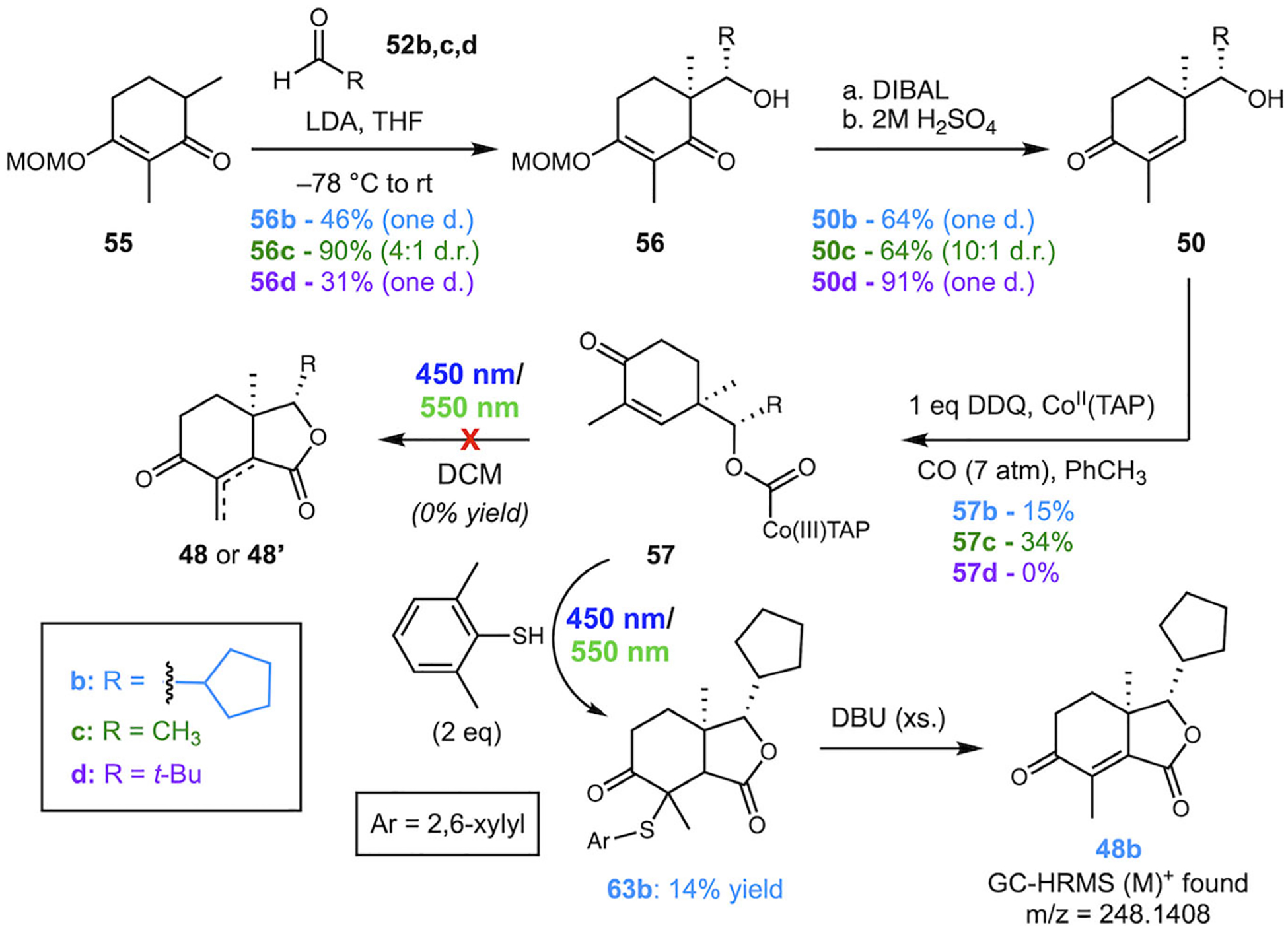
Synthesis and photolysis of alkyl analogs.

To determine the effect of sterics on the carbonylation reaction and if the lactonization proceeds when the 3-furyl group is replaced with an alkyl group, we next explored a series of substrates bearing methyl, cyclopentyl, and *tert*-butyl groups (Scheme 11, **50b**–**d**). Carbonylation yields decreased with increasing steric bulk (34%, 15%, 0%). In all cases, additive-free lactonization produced complex mixtures of products lacking key structural features of the desired products **48/48**’, indicating that numerous side reactions were occurring.

Interestingly, when 2,6-dimethylthiophenol was used as an additive in the lactonization of **57b**, a cyclized sulfide adduct **63b** was isolated in 14% yield, demonstrating that lactonization can occur in the absence of a furyl group. Further treatment of **63b** with DBU promoted elimination, providing less than a milligram of enone **48b**, which bears some similarity to the target compound fraxinellonone. Unfortunately, due to the low yields and difficult isolations of key species in the final steps, it was difficult to pursue more detailed studies of the lactonization reaction in this series of substrates. Furthermore, due to the particularly poor performance of the substrates bearing the biologically relevant 3-furyl group, further investigations were unfruitful and ultimately abandoned.

## 3 | Conclusion

We have investigated the effects of H-atom donor and ligand additives on the visible light photolysis of simple benzylic and several more complex unsaturated alkoxycarbonyl cobalt(III) species. In some cases, a polarity-matched H-atom donor provides good yields of reduced products, while in other cases, only products resulting from ß-hydrogen elimination are observed.

Furthermore, the effects of axial ligands and substrate electronics on the ß-hydrogen elimination and subsequent alkene isomerization mediated by Co(III) hydride species are reported. This study demonstrates the nuanced interplay between cobalt-mediated radical pathways, specifically ß-hydrogen elimination and MHAT, and how subtle changes in substrate structure, additive choice, and radical philicity can dramatically influence product outcomes. Through mechanistic investigations and additive studies, we established that while deoxygenation reactions of simple benzylic alcohols are highly tunable via electrophilic and nucleophilic hydrogen atom donors, lactonization pathways appear more resistant to such chemoselective tuning. The divergence in reactivity appears to stem from the nature of the radical intermediates formed post-lactonization, with radical polarity matching and radical stability playing a critical role in determining selectivity.

Our application of this chemistry to limonoid analog synthesis revealed both the promise and limitations of the approach. Although additive-free lactonization proved robust in simpler systems, the reaction failed to perform with substrates of increased complexity, where other side reactions became major issues. These findings underscore the importance of understanding radical behavior in complex environments and suggest that future efforts may benefit from designing substrates or catalysts that better exploit polarity effects or suppress competing pathways. Ultimately, this work lays the foundation for further exploration of cobalt-mediated radical transformations in natural product synthesis and highlights the mechanistic richness of these systems as both synthetic tools and platforms for fundamental discovery.

## Supplementary Material

Supplementary material file

Additional supporting information can be found online in the Supporting information section.

## Figures and Tables

**TABLE 1 |:** Additive effects on the photolysis of complex **28**.^a^

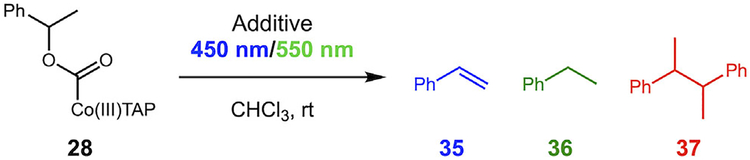				
Entry	Additive	35	36	37
1	No additive	11%	6%	41%
2	2,6-(Me)_2_PhSH	0%	48%	0%
3	**2,4,6-(iPr)**_**3**_ **PhSH, PPh**_**3**_	0%	43%	0%
4	**2,4,6-(iPr)**_**3**_ **PhSH** ^b^ **, HE**	0%	**94%**	0%
5	0.5 eq **2,4,6-(iPr)**_**3**_**PhSH**^b^**, HE**	0%	**86%**	0%
6	Bu_3_SnH	0%	54%	0%
7	(TMS)_3_SiH	0%	15%	22%
8	Me_3_HQ	22%	5%	0%
9	Benzimidazole	55%	0%	0%
10	**Me**_**3**_ **HQ, benzimidazole**	**91%**	0%	0%

a Conditions: 0.0133 M in CHCl_3_, 2 eq additive unless otherwise stated, under N_2_ in a sealed vessel. Yields determined by GC assay with benzodioxole as internal standard.

b 2 eq PPh_3_ also added. HE = Hantzsch ester. Me_3_HQ = trimethylhydroquinone.

**TABLE 2 |:** Lactonization studies with unsaturated alkoxycarbonyl complexes.^a^

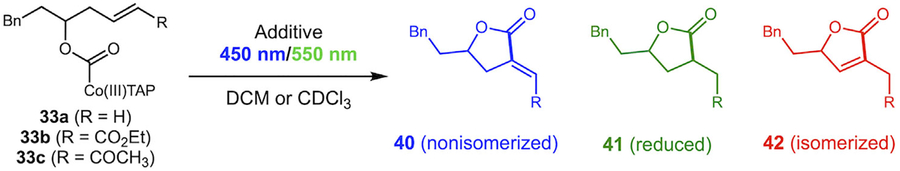					
Entry	Substrate	Additive	40	41 *(trans:cis)*	42
1	**33a**	No additive	0%	0%	68% (51%)
2	**33b**		0%	0%	68% (54%)
3	**33c**		0%	0%	44% (32%)
4	**33a**	0.5 eq **2,6-(Me)2PhSH** and 2 eq **Hantzch Ester**	0%	22% (13:87 d.r.)	37%
5	**33b**		0%	15% (n.d.)^b^	54%
6	**33a**	2 eq **Me**_**3**_**HQ** and 2 eq benzimidazole	49%	11% (0:100 d.r.)	30%
7	**33b**		0%	29% (51:49 d.r.)^c^	47%

a Conditions: 0.0125 M in CHCl_3_, under N_2_ in a sealed vessel. Yields determined by ^1^H NMR assay with benzodioxole as internal standard. Isolated yield in parentheses.

b n.d. = not determined due to partial peak overlap.

c Solvent is CDCl_3_.

## Data Availability

The data that support the findings of this study are available in the supplementary material of this article.
