# Supplementary material for: Additives Promote Divergent Reactivity in Photolytic Deoxygenation and Lactonization Reactions of Cobalt Alkoxycarbonyls
Source: European J Org Chem. Author manuscript; Available in PMC 2026 May 7. (PMC13148346; doi:10.1002/ejoc.202501091)
Supplement: Supplementary material file [file NIHMS2169234-supplement-Supplementary_material_file.pdf]

Supporting Information

Additives Promote Divergent Reactivity in Photolytic Deoxygenation and Lactonization Reactions of Cobalt Alkoxycarbonyls

Jacob N. Hackbarth[a], Dana R. Chambers[b], Cory T. Ludwig[a], Tyler K. Brown[a], David B.C. Martin[a]*

*Corresponding Author : david-martin@uiowa.edu

[a]Department of Chemistry, University of Iowa, Iowa City, Iowa 52242, United States.

[b]Department of Chemistry, University of California Riverside, Riverside, California 92521, United States.

General Methods:

All reactions were carried using oven dried or flame dried glassware charged with a magnetic stir bar and conducted under an inert nitrogen atmosphere using typical Schlenk techniques, unless otherwise noted. All solvents were dried by passage through columns of activated alumina or distilled and stored under nitrogen over freshly activated 4 Å sieves or otherwise freshly distilled. All starting materials were prepared according to known literature procedures or used as obtained from commercial sources, unless otherwise indicated. Reactions were monitored by thin-layer chromatography (TLC) and carried out on 0.25 mm coated commercial silica gel plates (Analtech TLC Uniplates, F254 precoated glass plates) using UV light as visualizing agent. Unless otherwise indicated, silica gel chromatography was performed using flash chromatography on P60 silica. ^{1}H and ^{13}C NMR spectra were recorded on a Bruker Avance NEO 400, Bruker Avance III 500, or Bruker Avance III 700 MHz spectrometer and were internally referenced to residual protio solvent signal (note: $CDCl_3$ referenced at δ 7.26 ppm for ^{1}H NMR and δ 77.16 ppm for ^{13}C NMR, respectively). Data for ^{1}H NMR are reported as follows: chemical shift (δ ppm), multiplicity (s = singlet, d = doublet, t = triplet, q = quartet, m = multiplet, app=apparent), coupling constant (Hz), and integration. Data for ^{13}C{1H} NMR are reported in terms of chemical shift and no special nomenclature is used for equivalent carbons. High-resolution mass spectrometry (HRMS) data were recorded on a Thermo Q-Exactive instrument (hybrid quadrupole Orbitrap) using direct injection of samples in dichloromethane diluted into acetonitrile/water (1:1) into the electrospray source (ESI) with positive ionization or negative ionization (i.e. HRMS (FTMS + p ESI)), or on an Agilent 7250 GCMS Q-TOF instrument with electron ionization (EI) using direct injection of samples in dichloromethane (i.e. GC QTOF). Gas Chromatography was carried out using a Shimadzu GC-2010 Plus instrument equipped with a Shimadzu SH-Rxi-5ms column. Column Specifications: 15 m (L), 0.25 mm (ID), 0. 25 µm (df), (diphenyl/dimethyl polysiloxane) stationary phase. UV-Vis experiments were carried out with an Agilent Cary 5000, with a total wavelength range of 175-3300 nm, but collecting data from 200 nm to 800 nm.

General Photochemical Methods:

A typical setup for a photoreaction in the Martin Lab utilizes an EvoluChem PhotoRedOx Box from Hepatochem (part number HCK1006-01-016). This provides fan cooling as well as mirrors to disperse light more evenly up to eight samples. PR160L and A160WE lamps from Kessil are used in experiments. Emission spectra provided by Kessil are included below. The PhotoRedOx box is placed on a stirplate to provide stirring.

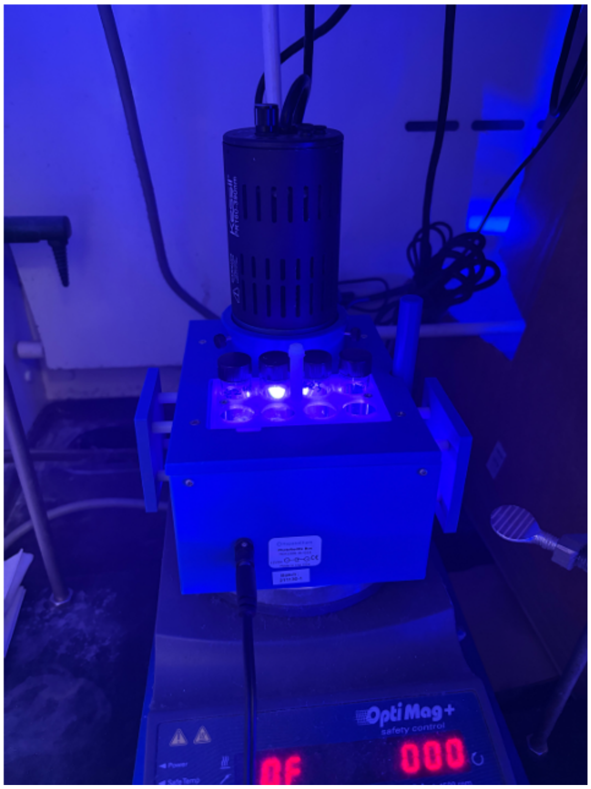

Pictured above: typical setup for photochemical reactions.

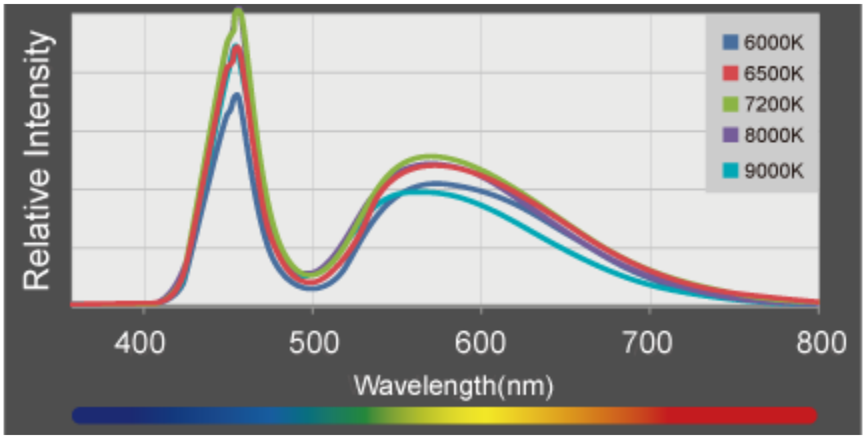

Emission Spectra for TUNA Sun A160WE Data and graphs provided by Kessil.

Safety Statement Caution! This work uses a 40 W LED for irradiation. Care should be taken to protect from exposure to heat and intense light. Tinted glasses are recommended when working

with light sources. Carbon monoxide (CO) gas is toxic and should be handled with care in a well-ventilated area.

General Procedures

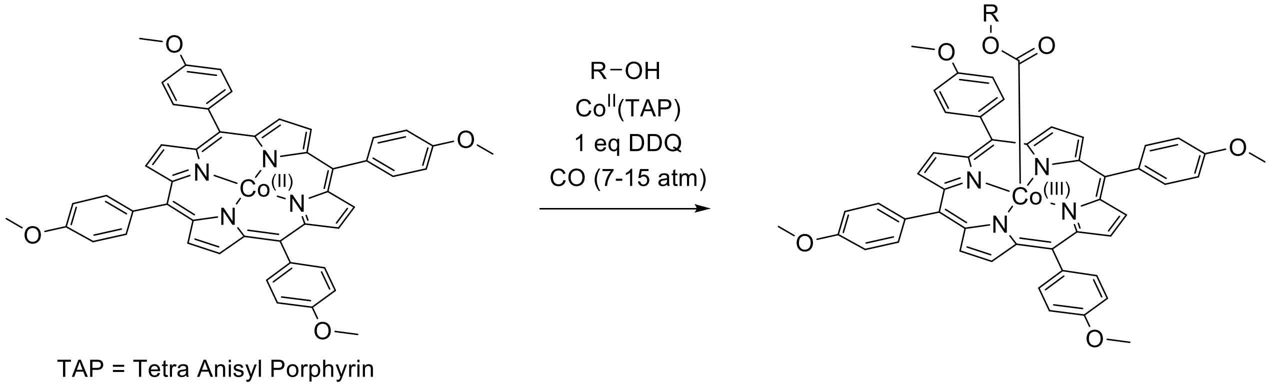

Procedure A – General Carbonylation Procedure: CO (g) is toxic and should be handled with care in a well-ventilated area. A 100 mL oven-dried round-bottom flask equipped with a Teflon stir bar was charged with DDQ (1.01 eq) and Co(TAP) (1.0 eq). Dichloromethane (0.0125 M) and alcohol (2.5 eq) were then added to the flask. The flask was placed in a Parr pressure vessel on a magnetic stir plate and pressurized to 7-10 atm of CO (g). The apparatus was cycled three times[i], purging with CO (g) in a fume hood. After pressurization, the stir plate was activated, and the reaction mixture was stirred overnight at room temperature.

Workup: The reaction mixture was filtered through a Hirsch funnel equipped with filter paper to remove solids (waste), which were rinsed with DCM. The eluent was concentrated on a minimal amount of oven-dried basic alumina[ii] to produce a free-flowing powder suitable for a dry load column. The product was obtained by running a dry-loaded basic alumina column[iii], initially loaded in hexanes, and eluted with a gradient from 1:10 DCM:hexanes to 100% DCM.[iv] To obtain a red foam, the product-containing fractions were identified on basic alumina TLC (red spot with RF[v] of

---

[i] When depressurizing Parr vessel, it is important to purge slowly with stir plate off to avoid the reaction mixture spontaneously leaving the round bottom to fill the Parr reactor.
[ii] Alternatively, good success has been seen from using celite for the dry loading step.
[iii] Use significantly less alumina than you would silica, only about 4 times the volume of the dryload and be wary of pressure at the beginning, as it packs differently than silica and has a larger particle size meaning a slower flow rate is needed to get good separation.
[iv] The point of the alumina is to remove the higher molecular weight alcohols; if you want to recover the alcohols try flushing with EtOAc.
[v] A purple spot with an RF of 1 is usually a cobalt porphyrin decomposition product similar to Co(TAP) and will be present on TLC but not necessarily in the sample being analyzed through 1H NMR analysis.

0.7-1 in 100% DCM) concentrated under reduced pressure (350 mbar) at 35-40 °C until a red syrup was formed, which was then subjected to high vacuum to give a crunchy foam.

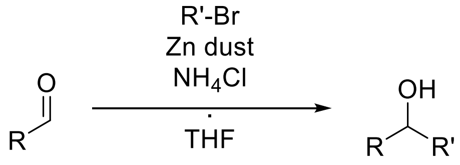

Procedure B – General Barbier Procedure: A round-bottomed flask was charged with a solution of the alkyl bromide (1.2-2 eq) in THF (0.25 M with respect to limiting reagent). Zinc dust (1.2-2 eq) as well as the aldehyde (1 eq) was added at 0 °C to the reaction mixture. The resulting suspension was stirred vigorously while a saturated NH_4Cl (aq) solution (1 reaction volume) was slowly added via addition funnel to initiate the reaction. The reaction was allowed to come to room temperature from 0 °C overnight. The mixture was then filtered through celite and extracted with EtOAc. The combined organic layers were washed with brine, dried with Na_2SO_4, and filtered. The filtrate was concentrated *in vacuo* to give the crude alcohol, which was purified by column chromatography (5–20 % EtOAc in petroleum ether).

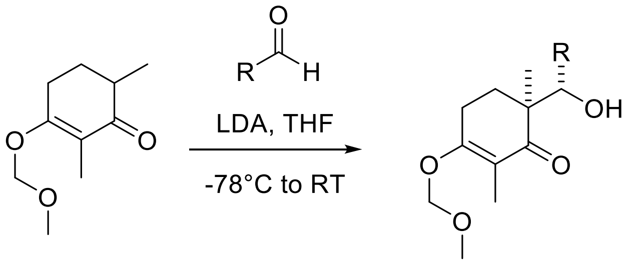

Procedure C – General Aldol Procedure: Lithium diisopropylamide (LDA) was prepared from diisopropylamine (DIPA, 1.02 eq) and 2.5 M[1],[vi] *n*-BuLi (9.4 mL, 1.05 eq) in THF (23 mL, 1 M) at 0 °C. To freshly prepared LDA in THF (1.02 eq, 1 M) was added vinylogous ester **54** (1.0 eq) at 0 °C and stirred for 1 hour to furnish a yellow/orange solution. The aldehyde[vii] (1.05 eq) was then added dropwise down the side of the flask into the enolate solution at –78 °C and stirred for 1-2 h at this temperature and quenched with saturated NH_4Cl (aq) solution. The product was then

[vi] *n*-BuLi was titrated against BHT using PDA as an indicator. See reference provided.
[vii] All aldehydes were distilled prior to use except for acetylaldehyde

extracted with ethyl acetate and dried with Na_2SO_4. The organic phase was then concentrated via rotary evaporation and purified via flash chromatography (EtOAc:hexanes).

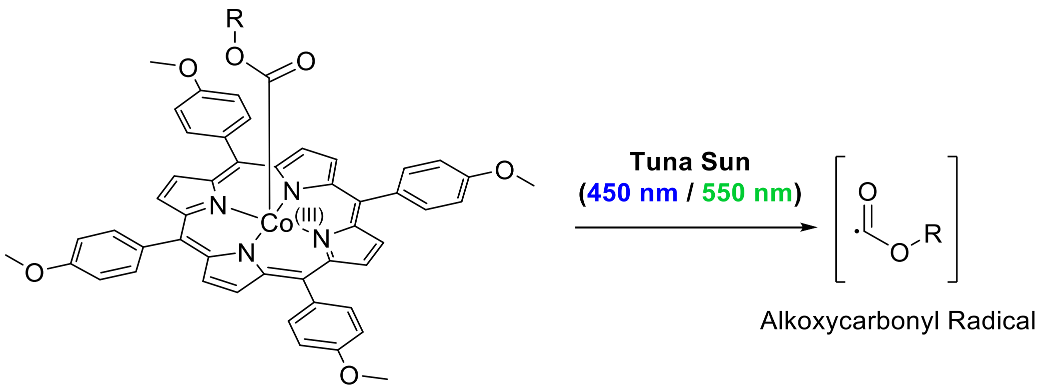

Procedure D – General Photolysis Procedure: To an oven dried 7 ml vial equipped with a stir bar was added Co(TAP) carbonylated complex (1 eq) and DCM (0.0125 M) then additives, if used, were added at this point. The vial was closed using a Teflon septa cap and was sparged with N_2 (g) via long 20G needle for 1 minute and sealed with parafilm both over the top of the septa cap as well as around the gap left by the cap. This vial was subjected to irradiation from a Tuna Sun white LED in a Hepatochem photoreactor for 16 hours. The vial was topped off with hexane or pentane to precipitate Co(II)TAP impurities and was filtered through a Monstr-pette celite plug, followed by a 2x1 ml DCM rinse of the precipitate to give NMR assayable[viii] crude product after rotary evaporation. All NMR assays utilized benzodioxole as an internal standard which was added via microsyringe to the crude concentrate before adding $CDCl_3$. For isolated products a gradient column was run 1:10 EtOAc:hexanes to 1:3 EtOAc:hexanes to furnish pure products.

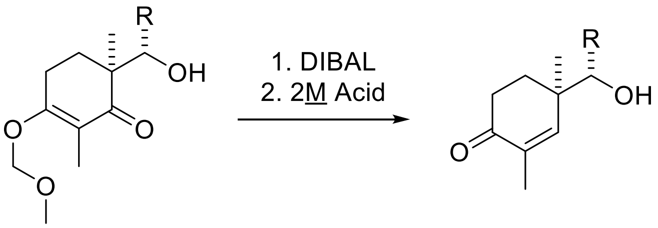

[viii] It was found that the paramagnetic nature of Co (II) would negatively affect the quality of the shims of the crude product and needed to be removed to better analyze NMR yields.

Procedure E – General Stork-Danheiser Procedure. To vinylogous ester (1 eq) in toluene (0.5 M), under N_2 (g) was added DIBAL (neat) (3 eq) at −78 °C and allowed to come to room temperature overnight. **IMPORTANT**: The reaction mixture was quenched very slowly at 0 °C with MeOH (10 ml), then add 2M H_2SO_4 (aq) and stir vigorously for 2 hours[ix] at RT. The aqueous phase was extracted with Et_2O then EtOAc, and concentrated *in vacuo* and purified via flash chromatography (EtOAc:hexanes) to give pure product.

Deoxygenation section

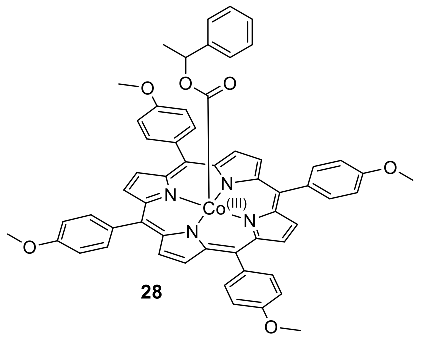

28

1-Phenethyloxycarbonylcobalt meso-(4-methoxyphenyl)porphyrin (28). From 1-Phenethanol (1.14 mL, 5 eq), Co(II)TAP (1.0420 g, 1 eq), DDQ (0.2988 g, 1 eq) according to carbonylation procedure A was obtained **28** (1.04 g, 80% yield) as a red crunchy foam. ^{1}H NMR was consistent with literature values.[2] ^{1}H NMR (500 MHz, $CDCl_3$) δ 8.90 (s, 8H), 7.98 (s, 8H), 7.24 (d, *J* = 8.3 Hz, 8H), 6.88 (t, *J* = 7.6 Hz, 1H), 6.73 (t, *J* = 7.7 Hz, 2H), 5.09 (d, *J* = 7.3 Hz, 2H), 4.07 (s, 12H), 3.27 (q, *J* = 6.4 Hz, 1H), -0.57 (d, *J* = 6.5 Hz, 3H).

Additive Study

All additive reactions were run according to general Procedure D. For deoxygenation, after celite plug to remove precipitated cobalt, benzodioxole was added as internal standard to the eluent which was run on GC without rotary evaporation and compared to retention time of prepared samples.

H_2 Evolution Experiment

A flame-dried round-bottom flask equipped with a magnetic stir bar was charged with cobalt tetra-anisyl porphyrin (0.1015 g, 0.128 mmol) and trimethoxyhydroquinone (0.038 g, 0.250 mmol)

[ix] This time was not optimized, but letting it go overnight caused a drastic decrease in yield

under inert atmosphere. The flask was evacuated under vacuum, then backfilled with argon. This evacuation and backfill cycle was repeated three times to ensure an inert atmosphere. The flask was equipped with a septum, and an oil bubbler was attached to the flask outlet to allow gas release while maintaining the inert atmosphere. A Forensics Detector FD-90A-H2 hydrogen gas meter was used to detect H_2 production by positioning the instrument's sampling pump inlet adjacent to the outlet of the oil bubbler. The detector, and sampling pump was turned on before dichloromethane (10 mL) was added to the flask via syringe, and the reaction mixture was stirred at room temperature. After 5 minutes of stirring, an argon balloon was connected to the flask headspace, which continuously purged the headspace through the oil bubbler and past the detector inlet. Under these conditions, hydrogen gas was successfully detected at a peak concentration of 22 ppm, confirming hydrogen evolution from the reaction mixture. When the same procedure was carried out using 2,6-dimethylthiophenol, no H2 was detected on the same timescale.

Lactonization

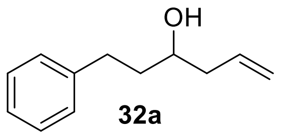

32a

1-Phenylhex-5-en-3-ol (32a). From hydrocinnamaldehyde (10.0879 g, 1 eq), allyl bromide (13 mL, 2 eq), zinc dust (9.83 g, 2 eq) and NH_4Cl (aq) (300 ml) in THF (300 mL, 0.25 M) via General procedure B was obtained (**32a**) (7.1671 g, 54%) as a yellow oil. ^{1}H NMR was consistent with literature values.[3] ^{1}H NMR (400 MHz, $CDCl_3$) δ 7.34 – 7.25 (m, 2H), 7.24 – 7.14 (m, 3H), 5.90 – 5.75 (m, 1H), 5.20 – 5.10 (m, 2H), 3.72 – 3.65 (m, 1H), 2.82 (ddd, *J* = 13.6, 8.6, 6.8 Hz, 1H), 2.69 (dt, *J* = 13.7, 7.7 Hz, 1H), 2.33 (dddt, *J* = 13.6, 6.6, 4.3, 1.3 Hz, 1H), 2.19 (dtd, *J* = 13.9, 7.9, 1.1 Hz, 1H), 1.88 – 1.71 (m, 2H), 1.60 (bs, 1H).

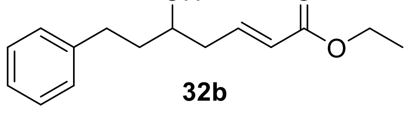

32b

Ethyl (E)-5-hydroxy-7-phenylhept-2-enoate (32b). Homoallylic alcohol (**32a**) (2.0015 g, 11.35 mmol, 1 eq) was added to a round bottom flask equipped with a Teflon stirbar. The flask was flushed and backfilled with inert gas (N_2), then DCM (113 mL, 0.1 M) was added. Grubbs II catalyst

(1.928 g, 0.227 mmol, 0.02 eq) and ethyl acrylate (3.1 mL, 2.5 eq, 28.38 mmol) were subsequently added, and the mixture was refluxed under an inert atmosphere for 16 hours. After completion, the reaction mixture was subjected to column chromatography, using a gradient from 5% to 20% ethyl acetate in hexanes, giving **32b** (2.3086 g, 82% yield) as a colorless oil. ^{1}H NMR was consistent with literature values.[4] ^{1}H NMR (600 MHz, $CDCl_3$) δ 7.29 (dd, *J* = 8.0, 7.0 Hz, 2H), 7.23 – 7.16 (m, 3H), 6.96 (ddd, *J* = 15.7, 7.8, 7.1 Hz, 1H), 5.90 (dt, *J* = 15.7, 1.5 Hz, 1H), 4.19 (q, *J* = 7.1 Hz, 2H), 3.78 (ddd, *J* = 12.4, 7.1, 4.8 Hz, 1H), 2.81 (ddd, *J* = 13.5, 8.6, 6.9 Hz, 1H), 2.69 (dt, *J* = 13.8, 7.8 Hz, 1H), 2.42 (dddd, *J* = 14.4, 7.1, 4.5, 1.6 Hz, 1H), 2.36 (dtd, *J* = 14.4, 7.7, 1.4 Hz, 1H), 1.86 – 1.75 (m, 2H), 1.29 (t, *J* = 7.1 Hz, 3H). OH peak did not resolve.

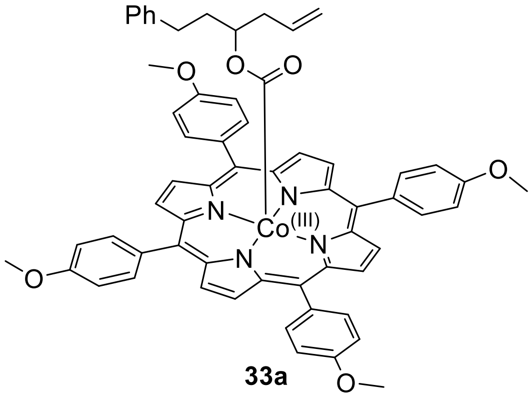

33a

1-Phenylhex-5-en-3-oxycarbonylcobalt meso-tetraphenylporphyrin (33a). From (32.A) (0.3273 g, 1.57 mmol, 2.5 eq), Co(II)TAP (0.5054 g, 0.63 mmol, 1 eq), and DDQ (0.1430 g, 0.63 mmol, 1.01 eq) in DCM (50 mL, 0.0125 M) under 7 atm of CO (g) via General procedure A was obtained (33a) (0.3683 g, 58 %) as a deep red-purple foam. ^{1}H NMR (400 MHz, $CDCl_3$) δ 8.91 (s, 8H), 8.01 (s, 8H), 7.23 (d, *J* = 8.1 Hz, 8H), 7.01 – 6.95 (m, 3H), 6.26 – 6.17 (m, 2H), 4.30 – 4.23 (m, 1H), 4.07 (s, 12H), 4.00 – 3.91 (m, 1H), 3.58 (ddt, *J* = 17.2, 10.2, 7.0 Hz, 1H), 2.31 (td, *J* = 9.1, 5.6 Hz, 1H), 0.45 (ddd, *J* = 14.7, 10.4, 4.8 Hz, 1H), 0.35 – 0.21 (m, 2H), 0.14 (dt, *J* = 13.9, 6.6 Hz, 1H), 0.01 (dddd, *J* = 13.6, 9.9, 6.4, 3.1 Hz, 1H), -0.14 (dtd, *J* = 14.3, 9.9, 4.9 Hz, 1H). ^{13}C{^{1}H} NMR (151 MHz, $CDCl_3$) δ 159.5, 146.00, 145.98, 141.0, 134.8, 134.4, 132.7, 132.7, 132.1, 128.2, 128.0, 125.6, 121.7, 116.4, 112.4, 74.8, 55.7, 37.4, 34.4, 29.6. HRMS (FTMS + p ESI) m/z: $[M]^+$ Calculated for $C_{61}H_{51}CoN_4O_6$ 944.3135; Found 944.3125

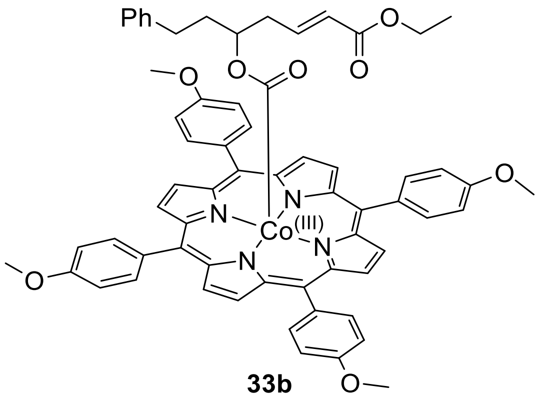

33b

Ethyl (E)-5-oxycarbonylcobalt meso-tetraphenylporphyrin-7-phenylhept-2-enoate (33b). From (**32b**) (0.85 g, 3.45 mmol, 2.5 eq), Co(II)TAP (1.085 g, 1.38 mmol, 1 eq), and DDQ (0.3130 g, 1.38 mmol, 1.01 eq) in DCM (110 mL, 0.0125 M) under 7 atm of CO (g) via General procedure A was obtained **33b** (0.8357 g, 56 %) as a deep red foam. ^{1}H NMR (400 MHz, $CDCl_3$) δ 8.93 (s, 8H), 8.02 (bs, 8H), 7.23 (d, *J* = 8.2 Hz, 8H), 7.04 – 6.96 (m, 3H), 6.24 – 6.13 (m, 2H), 5.10 (dt, *J* = 15.1, 7.4 Hz, 1H), 4.78 (d, *J* = 15.6 Hz, 1H), 4.07 (s, 12H), 4.08 – 4.00 (m, 1H), 2.38 – 2.30 (m, 1H), 1.35 – 1.23 (m, 2H), 1.20 (t, *J* = 7.1 Hz, 3H), 0.50 (ddd, *J* = 14.0, 10.1, 4.7 Hz, 1H), 0.38 – 0.18 (m, 2H), 0.11 – -0.05 (m, 1H), -0.12 (ddt, *J* = 14.2, 9.4, 4.8 Hz, 1H). ^{13}C{^{1}H} NMR (101 MHz, $CDCl_3$) δ 165.6, 159.5, 145.9, 141.8, 140.6, 134.9, 134.3, 132.8, 132.8, 128.1, 128.1, 125.7, 123.3, 121.8, 112.5, 73.3, 60.1, 55.7, 35.8, 34.2, 29.5, 14.4. HRMS (FTMS + p ESI) m/z: $[M]^+$ Calculated for $C_{64}H_{55}CoN_4O_8$ 1066.3360; Found 1066.3355

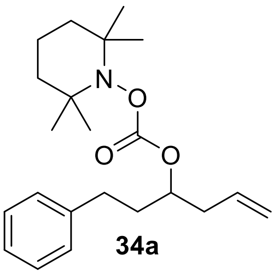

34a

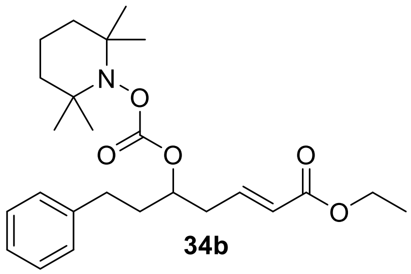

34b

TEMPO Adducts 34a and 34b. From respective compound **33** (a or b) according to general procedure D, 4 eq of TEMPO was used as the additive, enriched samples of **34** were obtained as the major product after silica gel chromatography (in each case about 65% yield by mass). However, due to issues with ^{1}H NMR resolution of the products (either due to residual paramagnetic TEMPO or rotational restrictions of the products leading to varied peak broadening), ^{13}C NMR and HRMS were relied upon for conclusive evidence of the major product formation. The purity was not adequately determined with the methods used. For **34a** 13C ^{13}C{^{1}H}

NMR (101 MHz, CDCl3) δ 154.2, 139.0, 130.8, 126.1, 126.0, 123.6, 115.8, 74.3, 58.0, 36.8, 36.4, 33.2, 29.2, 29.2, 20.3, 18.1, 14.6, 11.8. HRMS (FTMS + p ESI) m/z: $[M+H]^+$ Calculated for $C_{22}H_{34}O_3N$ 360.2533; Found 360.2529. For **34b** $^{13}C\{^1H\}$ NMR (101 MHz, CDCl3) δ 161.2, 151.7, 138.2, 136.2, 123.9, 123.7, 121.5, 119.9, 71.0, 55.7, 55.7, 34.5, 32.5, 31.2, 26.9, 26.8, 15.8, 15.8, 12.3, 9.7. HRMS (FTMS + p ESI) m/z: $[M+H]^+$ Calculated for $C_{25}H_{38}O_5N$ 432.2744; Found 432.2740.

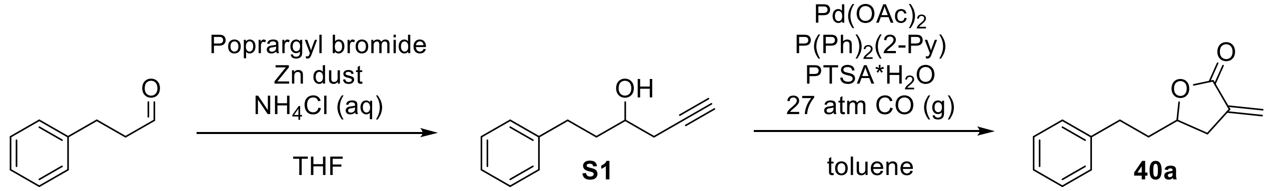

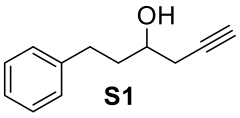

S1

1-Phenylhex-5-yn-3-ol (S1). To hydrocinnamaldehyde (2.3677 g, 1 eq), propargyl bromide (Technical grade 80%) (3.83mL[x], 2 eq), and zinc dust (2.30 g, 2 eq) in THF (88 mL, 0.25 M) was added NH_4Cl (aq) (88 ml) according to General procedure B giving (**S1**) (1.4787 g, 92:8 alkyne:allene, 48%[xi]) as a yellow oil. HNMR was consistent with literature values.[5] ^1H NMR (500 MHz, $CDCl_3$) δ 7.33 – 7.27 (m, 2H), 7.24 – 7.13 (m, 3H), 3.79 (ddd, *J* = 12.1, 6.7, 4.9 Hz, 1H), 2.86 – 2.66 (m, 2H), 2.45 (ddd, *J* = 16.7, 4.7, 2.7 Hz, 1H), 2.36 (ddd, *J* = 16.7, 6.8, 2.7 Hz, 1H), 2.06 (t, *J* = 2.7 Hz, 1H), 1.90 – 1.85 (m, 2H). OH peak did not resolve. Middle of allene multuplets at 4.88 ppm (2H) and 5.29 ppm (1H).

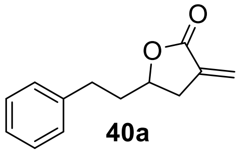

40a

3-Methylene-5-phenethyldihydrofuran-2(3H)-one (40a). Method adapted from Dong *et al.*.[6]: To a flame dried round bottom flask charged with a teflon stirbar was added (**S1**) (1.4787 g, 1 eq),

---

[x] Adjusted by 1.2 x to accommodate for the 80% purity of the reagent grade material.
[xi] 8:92 ratio between allene and homopropargyillic product

$Pd(OAc)_2$ (0.0386 g, 0.02 eq), diphenyl-2-pyridyl phosphine (0.1810 g, 0.08 eq), and PTSA*H_2O (0.2620 g, 0.16 eq) in toluene (16 mL, 0.33 M) and was placed in a parr reactor and purged[xii] with five cycles of CO (10 atm) and was finally pressurized to 27 atm of CO (g) and allowed to react at room temperature for 16 hours under vigorous stirring. The reaction was depressurized after the stir plate was turned off. The reaction was subsequently quenched with $NaHCO_3$ (aq), extracted with EtOAc, and dried over Na_2SO_4. The crude was concentrated *in vacuo* and was taken up in DCM and flushed through a silica plug to remove excess Pd waste giving the desired lactone product (**40a**) (1.5634 g, 90%) as a colorless oil without the need for further purification. HNMR was consistent with literature values.[7] ^{1}H NMR (500 MHz, $CDCl_3$) δ 7.33 – 7.27 (m, 2H), 7.24 – 7.14 (m, 3H), 6.24 (t, *J* = 2.9 Hz, 1H), 5.63 (t, *J* = 2.5 Hz, 1H), 4.50 (tdd, *J* = 8.0, 6.2, 4.8 Hz, 1H), 3.05 (ddt, *J* = 17.0, 7.7, 2.5 Hz, 1H), 2.84 (ddd, *J* = 14.5, 9.3, 5.4 Hz, 1H), 2.75 (ddd, *J* = 13.9, 9.0, 7.2 Hz, 1H), 2.59 (ddt, *J* = 17.1, 6.0, 2.9 Hz, 1H), 2.05 (dtd, *J* = 14.1, 8.7, 5.5 Hz, 1H), 1.93 (dddd, *J* = 14.0, 9.4, 7.2, 4.8 Hz, 1H).

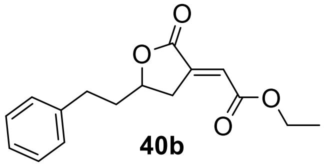

40b

(E)-Ethyl 2-(2-oxo-5-phenethyldihydrofuran-3(2H)-ylidene)acetate (40b). From Co(III)TAP carbonylation adduct (**33b**) (0.0764 g, 1 eq), and DMAP[xiii] (0.0087 g, 1 eq) in DCM (6 mL, 0.0125 M) was obtained (40b) (0.0024 g, 12%) via procedure D as a colorless oil. E/Z assignment was done via comparison to J values and chemical shift data for known compounds of similar structure in the literature.[8] ^{1}H NMR (600 MHz, $CDCl_3$) δ 7.33 – 7.28 (m, 2H), 7.25 – 7.18 (m, 3H), 6.76 (t, *J* = 3.2 Hz, 1H), 4.60 (tt, *J* = 8.0, 5.2 Hz, 1H), 4.25 (q, *J* = 7.1 Hz, 2H), 3.50 (ddd, *J* = 20.1, 7.6, 3.0 Hz, 1H), 2.95 (ddd, *J* = 20.1, 5.5, 3.3 Hz, 1H), 2.84 (ddd, *J* = 14.5, 9.2, 5.6 Hz, 1H), 2.80 – 2.74 (m, 1H), 2.10 – 2.01 (m, 1H), 2.01 – 1.92 (m, 1H), 1.32 (t, *J* = 7.1 Hz, 3H). $^{13}C\{^1H\}$ NMR (151 MHz, $CDCl_3$) δ 169.8, 165.5, 142.3, 140.5, 128.8, 128.6, 126.5, 124.7, 78.0, 61.4, 38.3, 34.0, 31.4, 14.3. HRMS (FTMS + p ESI) m/z: $[M + H]^+$ Calculated for $C_{16}H_{19}O_4$ 275.1278; Found 275.1278.

[xii] When depressurizing parr vessel, it is important to purge slowly with stir plate off to avoid the reaction mixture spontaneously leaving the round bottom to fill the parr reactor.
[xiii] This was found as an isolable product in a separate additive screen exploring the selectivity of these reactions in the presence of other additives. These results will be reported in due course.

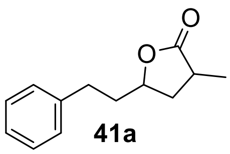

41a

Dihydro-3-methyl-5-(2-phenylethyl)-2(3*H*)-furanone. (41a) A cis-41a enriched product was made through the hydrogenation of **42a** using Pd on C giving a cis enriched 41a with a d.r. of 10:90 trans:cis. These reaction results were compared with literature values for a 1:1 d.r. of cis and trans isomers of the exact compound[9], and assigned using the findings of Hoye, et al.[10] ^{1}H NMR (400 MHz, $CDCl_3$) δ 7.33 – 7.27 (m, 2H), 7.24 – 7.18 (m, 3H), 4.32 (ddt, *J* = 10.2, 8.0, 5.1 Hz, 1H), 2.94 – 2.53 (m, 3H), 2.47 (ddd, *J* = 12.3, 8.6, 5.4 Hz, 1H), 2.17 – 1.75 (m, 2H), 1.53 (td, *J* = 12.2, 10.3 Hz, 1H), 1.27 (d, *J* = 7.0 Hz, 3H).

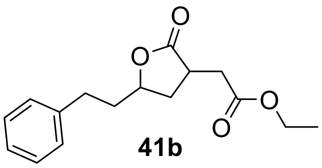

41b

Ethyl 2-(2-oxo-5-phenethyltetrahydrofuran-3-yl)acetate. (41b) From Co(III)TAP carbonylation adduct (**33b**) (0.1055 g, 1 eq) and Tributyltin hydride (0.090 mL, 3 eq) in DCM (8.9 mL, 0.0125 M) was obtained **(41b)** (0.0205 g, 75% 37:63 trans:cis) via procedure D as a red oil[xiv]. A modified workup was used where the crude reaction was partitioned between acetonitrile and hexanes and was extracted 3 times with hexanes to remove excess tin biproducts. The cis/trans isomers were assigned in accordance with literature regarding this motif in acetogenin natural products.[9] ^{1}H NMR (500 MHz, $CDCl_3$) δ 7.33 – 7.26 (m, 2H), 7.24 – 7.17 (m, 3H), 4.62 – 4.33 (m, 1H), 4.17 (q, *J* = 7.1 Hz, 2H), 3.10 – 2.97 (m, 1H), 2.97 – 2.67 (m, 4H), 2.61 – 2.53 (m, 1H), 2.56 – 2.47 (m, 1H), 2.26 – 2.08 (m, 1H), 2.11 – 1.99 (m, 1H), 1.99 – 1.82 (m, 1H), 1.27 (t, *J* = 7.1 Hz, 3H). $^{13}C\{^1H\}$ NMR (126 MHz, $CDCl_3$) δ **178.0**, 177.5, 171.3, **171.2**, 140.8, **140.7**, 128.8, 128.7, 128.6, 128.6, 128.6, 126.3, 78.1, **77.8**, **61.2**, 61.1, 37.8, **37.5**, 37.3, 35.7, 35.2, **35.2**, 34.7, 33.1, **31.8**, 31.8, 14.3, **14.3**.[xv] HRMS (GC QTOF) m/z: Calculated for $C_{16}H_{20}O_4$ 276.1356; Found 276.1360

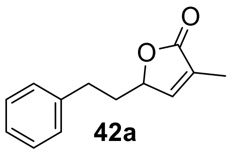

42a

[xiv] The color was due to residual Co(II)TAP

[xv] All bolded ^{13}C signals indicate the resolved signals for the minor trans isomer.

3-Methyl-5-phenethylfuran-2(5H)-one (42a).

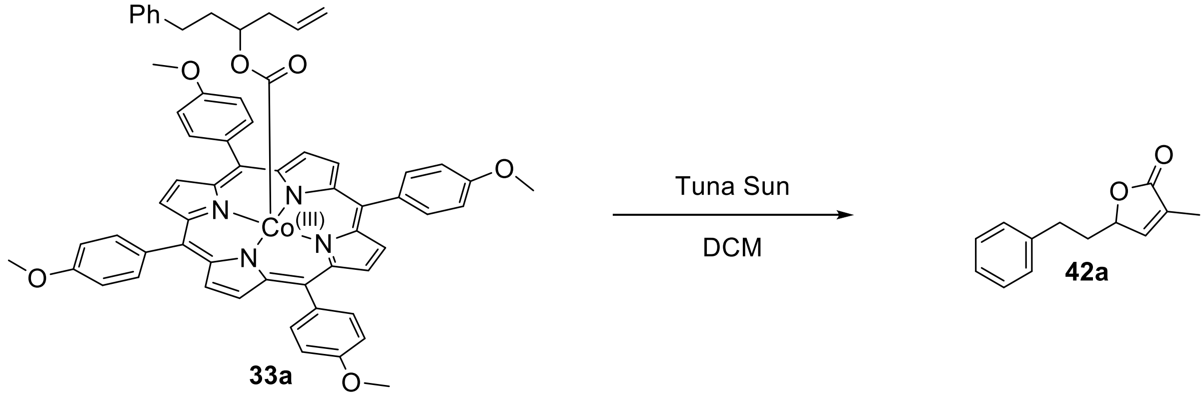

Photolactonization: Co(III)TAP carbonylation adduct (**33a**) (0.0759 g, 1 eq) was mixed in DCM (6.1 mL, 0.0125 M) and photoirradiated for 16 hours to obtain (**42a**) (0.0078 g, 51%) via general procedure D as a colorless oil. HNMR was consistent with literature values.[11] ^{1}H NMR (400 MHz, $CDCl_3$) δ 7.34 – 7.25 (m, 2H), 7.21 (td, *J* = 7.4, 6.8, 1.5 Hz, 3H), 6.97 (p, *J* = 1.6 Hz, 1H), 4.92 – 4.82 (m, 1H), 2.89 – 2.70 (m, 2H), 2.03 (dddd, *J* = 13.8, 9.1, 7.3, 4.6 Hz, 1H), 1.97 – 1.83 (m, 4H).

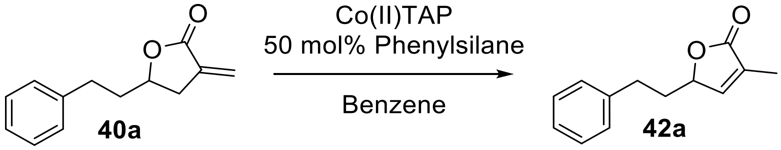

Method Adapted from Shenvi *et al.*[12] - Co(II)TAP/$PhSiH_3$ isomerization: In an oven dried 7 mL vial equipped with teflon stirbar was added Exocyclic alkene (**40a**) (0.010 g, 1 eq), Co(II)TAP (0.0051 g, 0.1 eq), and $PhSiH_3$ (1 μL, 0.1 eq) were added to benzene (2 mL, 0.025 M) and stirred for 24 hours at room temperature without photoirradiation. The vial was topped off with hexanes to precipitate Co(II)TAP impurities[xvi] and was filtered through a monster pipet celite plug, followed by a 2x1 ml DCM rinse of the plug. The eluent was concentrated *in vacuo* to give NMR assayable[xvii] crude product which showed complete isomerization of starting material to endocyclic alkene (42a).

[xvi] It was found that the paramagnetic nature of Co (II) would negatively affect the quality of the shims of the crude product and needed to be removed to better analyze NMR yields.

[xvii] 7 μL of benzodioxole as internal standard was added after concentration of the eluent.

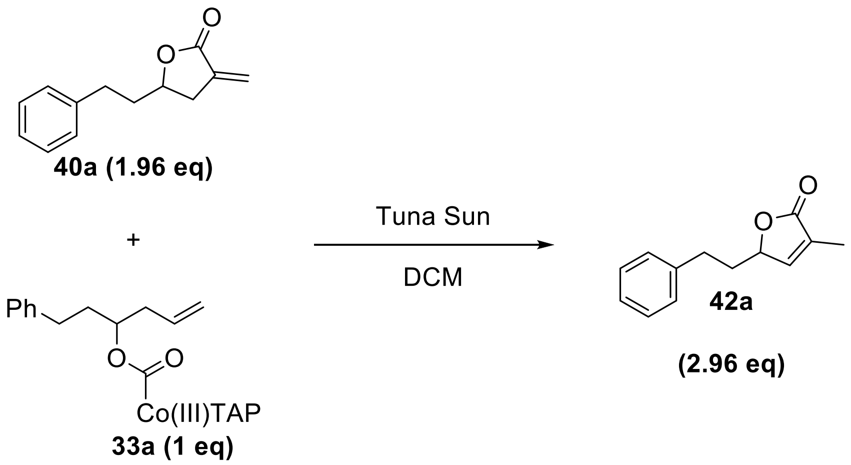

Mixed mechanism inquiry: To Co(III)TAP carbonylation adduct (**33b**) (0.0110 g, 0.33 eq) in DCM (1 mL, 0.0125 M) was added exocyclic lactone 40a (0.0041 g, 0.66 eq) to obtain ***exclusively*** endocyclic lactone **(42a)** (99%[xviii]) as observed by HNMR assay using 3 μL of benzodioxole as internal standard.

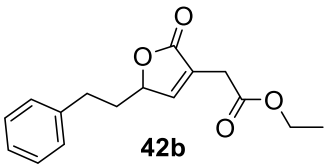

42b

±-Ethyl 2-(2-oxo-5-phenethyl-2,5-dihydrofuran-3-yl)acetate (42b).

Photolactonization: From Co(III)TAP carbonylation adduct (**33b**) (0.0750 g, 1 eq) in DCM (2.8 mL, 0.025 M) was obtained **(42b)** (0.0103 g, 54%) via procedure D as a colorless oil. ^{1}H NMR (600 MHz, $CDCl_3$) δ 7.35 (q, *J* = 1.5 Hz, 1H), 7.33 – 7.26 (m, 2H), 7.24 – 7.18 (m, 3H), 4.97 (ddt, *J* = 8.2, 4.3, 1.7 Hz, 1H), 4.18 (q, *J* = 7.1 Hz, 2H), 3.33 (s, 2H), 2.88 – 2.74 (m, 2H), 2.13 – 2.03 (m, 1H), 1.99 – 1.90 (m, 1H), 1.27 (t, *J* = 7.1 Hz, 3H). ^{13}C{^{1}H} NMR (151 MHz, $CDCl_3$) δ 173.1, 169.5, 151.5, 140.5, 128.8, 128.7, 127.2, 126.5, 81.0, 61.5, 35.2, 31.5, 30.6, 14.3. HRMS (FTMS + p ESI) m/z: $[M + H]^+$ Calculated for $C_{16}H_{19}O_4$ 275.1278; Found 275.1278

[xviii] Theoretical yield was calculated via the summation of the carbonylation adduct moles and the added exocyclic lactone moles. Thus a 66% of the product is accounted for through isomerization of **40a** and the other 33% is through photolactonization. It is interesting to note that with α-ß unsaturated carbonyl added in the beginning that the lactonization went from 68% to effectively quantitative.

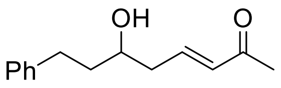

32c

(E)-6-hydroxy-8-phenyloct-3-en-2-one (32c) Homoallylic alcohol (**32a**) (1.2101 g, 6.68 mmol, 1 eq) was added to a round bottom flask equipped with a Teflon stirbar. The flask was flushed and backfilled with inert gas (N_2), then DCM (80 mL, 0.1 M) was added. Grubbs II catalyst (0.058 g, 0.06 mmol, 1 mol %) and Methyl Vinyl Ketone (2.8 mL, 5 eq, 34 mmol) were subsequently added, and the mixture was refluxed under an inert atmosphere for 16 hours. After completion, the reaction mixture was subjected to column chromatography, using a gradient from 5% to 20% ethyl acetate in hexanes, giving **32c** (1.3051 g, 87% yield) as a colorless oil. ^{1}H NMR (500 MHz, $CDCl_3$) δ 7.40 – 7.30 (m, 2H), 7.30 – 7.21 (m, 3H), 6.89 (dt, *J* = 15.7, 7.3 Hz, 1H), 6.20 (d, *J* = 15.9 Hz, 1H), 3.92 – 3.77 (m, 1H), 2.94 – 2.81 (m, 1H), 2.81 – 2.70 (m, 1H), 2.56 – 2.40 (m, 2H), 2.31 (s, 3H), 2.02 – 1.95 (m, 1H), 1.93 – 1.83 (m, 2H). ^{13}C NMR (126 MHz, $CDCl_3$) δ 198.6, 144.3, 141.6, 133.6, 128.6, 128.5, 126.1, 70.0, 40.7, 38.9, 32.1, 27.1. HRMS (FTMS + ESI) m/z: $[M+H]^+$ Calculated for $C_{14}H_{19}O_2$ 219.1380; Found 219.1380.

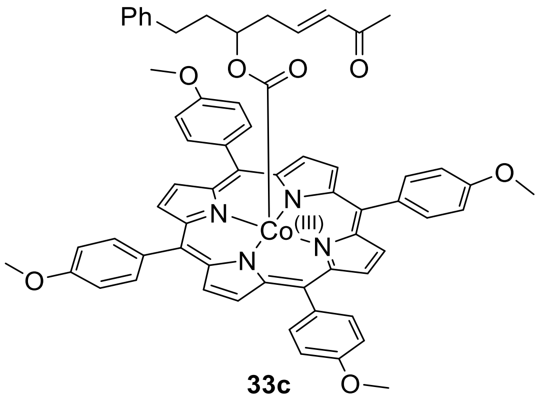

33c

((E)-6-hydroxy-8-phenyloct-3-en-2-one) carbonylcobalt meso-tetraphenylporphyrin (33c) From (**32c**) (0.70 g, 3.24 mmol, 2.5 eq), Co(II)TAP (1.0285 g, 1.3 mmol, 1 eq), and DDQ (0.29 g,

1.38 mmol, 1.1 eq) in DCM (80 mL, 0.0125 M) under 7 atm of CO (g) via General procedure A was obtained **33c** (0.5857 g, 43 %) as a deep red foam. ^{1}H NMR (500 MHz, $CDCl_3$) δ 8.94 (s, 1H), 8.96 – 8.91 (m, 8H), 8.02 (s, 8H), 7.23 (d, *J* = 8.2 Hz, 8H), 7.03 – 6.93 (m, 3H), 6.13 – 6.08 (m, 2H), 4.99 (d, *J* = 15.9 Hz, 1H), 4.50 (dt, *J* = 16.1, 7.2 Hz, 1H), 4.07 (s, 12H), 1.69 (s, 3H), 0.63 (ddd, *J* = 13.7, 9.4, 4.7 Hz, 1H), 0.49 (dddd, *J* = 14.6, 6.6, 4.7, 1.5 Hz, 1H), 0.41 – 0.28 (m, 1H), 0.19 (ddd, *J* = 13.3, 9.2, 7.3 Hz, 1H), 0.06 (dddd, *J* = 12.8, 9.9, 7.3, 3.0 Hz, 1H), -0.09 (dtd, *J* = 13.9, 9.4, 4.7 Hz, 1H). ^{13}C NMR (126 MHz, $CDCl_3$) δ 198.2, 159.5, 145.9, 141.6, 140.3, 134.8, 132.8, 128.1, 125.8, 121.8, 112.6, 73.2, 55.7, 36.7, 35.2, 34.9, 34.6, 29.4, 27.4, 26.4. HRMS (FTMS + p ESI) m/z: $[M]^+$ Calculated for $C_{63}H_{53}O_7N_4Co$ 1036.3241; Found 1036.3238.

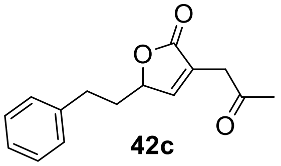

42c

3-(2-oxopropyl)-5-phenethylfuran-2(5H)-one (42c) From Co(III)TAP carbonylation adduct (**33c**) (0.1058 g, 1 eq) in DCM (6 mL, 0.0125 M) was obtained (**42c**) (0.0080 g, 32%) via procedure D as a colorless oil. In a separate experiment, 0.02360 g of **33c** was subjected to General Procedure D, and the crude product was analyzed by NMR using 3 µL of benzodioxole as an internal standard, giving a calculated yield of 44%. ^{1}H NMR (400 MHz, $CDCl_3$) δ 7.37 – 7.27 (m, 3H), 7.24 – 7.14 (m, 3H), 5.08 – 4.82 (m, 1H), 3.45 (s, 2H), 2.90 – 2.74 (m, 2H), 2.25 (s, 3H), 2.16 – 2.02 (m, 1H), 2.02 – 1.87 (m, 1H). ^{13}C NMR (101 MHz, $CDCl_3$) δ 203.1, 173.5, 152.2, 140.5, 128.8, 128.7, 126.9, 126.5, 81.1, 39.0, 35.2, 31.5, 30.4. HRMS (FTMS + p ESI) m/z: $[M+H]^+$ Calculated for $C_{15}H_{17}O_3$ 245.1172; Found 245.1169.

Limonoids section

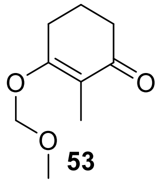

53

3-(Methoxymethoxy)-2-methylcyclohex-2-en-1-one (53). To a stirred solution of 2-methyl-1,3-cyclohexanedione (10.0176 g, 1 eq)[xix] in dry DMF (375 mL, 0.21 M) was added NaH 60% wt in mineral oil (3.3992 g, 1.05 eq) portionwise over 5 minutes (Caution! NaH DMF mixtures have been known to generate runaway exotherms at elevated temperatures[13]). The resultant mixture was stirred at room temperature for 30 minutes until homogenous. Reagent grade MOMCl (6.31 mL,1.05 eq) was added directly to the reaction mixture via long leuer lock needle and monitored via TLC[xx]. Once complete, the reaction was quenched with a volume of water[xxi] equal to the DMF used for the reaction and was extracted with ether, followed by EtOAc. The organic phase was then washed 3-4 times with saturated aqueous LiCl solution to remove the DMF. The organic phase was concentrated *in vacuo* and was purified via flash chromatography[xxii] (EtOAc:hexanes) to give **53** (11.9804 g, 89%) as a yellow oil. ^{1}H NMR (400 MHz, $CDCl_3$) δ 5.10 (s, 2H), 3.42 (s, 3H), 2.57 (tt, *J* = 6.2, 1.7 Hz, 2H), 2.33 (dd, *J* = 7.4, 6.0 Hz, 2H), 2.01 – 1.89 (m, 2H), 1.71 (t, *J* = 1.6 Hz, 3H). $^{13}C\{^1H\}$ NMR (101 MHz, $CDCl_3$) δ 199.3, 169.4, 117.1, 92.7, 56.5, 36.6, 25.1, 21.0, 7.5. HRMS (FTMS + p ESI) m/z: $[M + H]^+$ Calculated for $C_9H_{15}O_3$ 171.1016; Found 171.1014

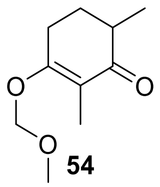

54

3-(methoxymethoxy)-2,6-dimethylcyclohex-2-en-1-one (54). To freshly prepared 1M LDA in THF (1.25 eq) was added a solution of compound (**53**) (5.0226 g, 1.0 eq) in THF (22 mL, 1.35 M with respect to **53**) dropwise via cannula at 0 °C. After 30 minutes dry DMPU[xxiii] (2.84 mL, 0.8 eq), and MeI (2.74 mL, 1.5 eq) were added at -78 °C. The reaction was then monitored via TLC and quenched with one reaction volume of water and extracted with ether, then ethyl acetate and dried

[xix] This starting material is water soluble and sticks in the aqueous phase during workup
[xx] Reaction was usually complete under an hour.
[xxi] If excess MOMCl is used then the addition of water was found to generate enough HCl to foster the deprotection of the product giving some starting material after quench that was not present prior to quench.
[xxii] Alternatively, kugelrohr distillation was able to be used to effectively get product.
[xxiii] Reaction yield is closely tied to DMPU purity especially dryness, thus DMPU, even freshly opened from commercial sources was stored over 4 angstrom molecular sieves before use. A quick test for DMPU dryness is to use a spatula tip of NaH and mix with neat DMPU, if gas evolves that indicates wet DMPU.

with sodium sulfate. The organic phase was concentrated *in vacuo* and was purified via flash chromatography (EtOAc:hexanes) to give **54** (4.1505 g, 76%) as a yellow oil. ^{1}H NMR (400 MHz, $CDCl_3$) δ 5.10 (s, 2H), 3.43 (s, 3H), 2.72 – 2.61 (m, 1H), 2.60 – 2.49 (m, 1H), 2.27 (dqd, *J* = 11.5, 6.9, 4.7 Hz, 1H), 2.05 (dq, *J* = 13.1, 4.8 Hz, 1H), 1.74 – 1.59 (m, 4H), 1.12 (d, *J* = 6.9 Hz, 3H). ^{13}C{^{1}H} NMR (101 MHz, $CDCl_3$) δ 201.6, 168.3, 116.3, 92.5, 56.5, 39.8, 29.0, 24.4, 15.7, 7.9. HRMS (FTMS + p ESI) m/z: $[M + H]^+$ Calculated for $C_{10}H_{17}O_3$ 185.1178; Found 185.1171

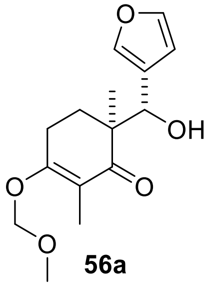

56a

±-(S)-6-((S)-furan-3-yl(hydroxy)methyl)-3-(methoxymethoxy)-2,6-dimethylcyclohex-2-en-1-one (56a). From freshly prepared 1.0 M LDA in THF (1.05 eq), vinylogous ester **54** (4.0884 g, 1 eq), 3-furfural (2.45 mL, 1.05 eq) via the general aldol procedure was obtained **56a** (5.0685 g, 81%) as a white solid. ^{1}H NMR (500 MHz, $CDCl_3$) δ 7.39 – 7.32 (m, 2H), 6.39 – 6.35 (m, 1H), 5.17 – 5.08 (m, 3H), 4.83 (bs, 1H), 3.45 (s, 3H), 2.64 (dddd, *J* = 18.1, 5.8, 3.2, 1.4 Hz, 1H), 2.59 – 2.47 (m, 1H), 1.76 – 1.67 (m, 4H), 1.51 (ddd, *J* = 13.4, 5.3, 3.1 Hz, 1H), 1.16 (s, 3H). ^{13}C{^{1}H} NMR (126 MHz, $CDCl_3$) δ 206.6, 169.5, 142.6, 140.7, 124.2, 115.0, 110.4, 92.7, 72.1, 56.7, 45.6, 29.9, 21.7, 15.0, 7.8. HRMS (FTMS + p ESI) m/z: $[M + H]^+$ Calculated for $C_{15}H_{21}O_5$ 281.1384; Found 281.1381

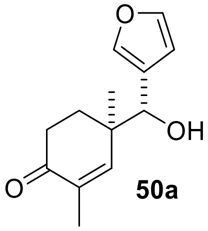

50a

±-(R)-4-((R)-furan-3-yl(hydroxy)methyl)-2,4-dimethylcyclohex-2-en-1-one (50a). From vinylogous ester 56a (1.2099 g, 1 eq) in toluene (9 mL, 0.5 M), DIBAL (2.29 mL, 3 eq) according to general Stork-Danheiser Procedure to give enone **50a** (0.6763 g, 71% >95:5 d.r.) as an opaque off white crystalline solid. ^{1}H NMR (500 MHz, $CDCl_3$) δ 7.40 (d, *J* = 1.4 Hz, 2H), 6.75 – 6.69 (m, 1H), 6.38 (t, *J* = 1.4 Hz, 1H), 4.61 (d, *J* = 3.4 Hz, 1H), 2.55 – 2.41 (m, 2H), 2.02 (ddd, *J* = 13.5, 10.1, 6.1 Hz, 1H), 1.88 (d, *J* = 3.6 Hz, 1H), 1.79 (d, *J* = 1.4 Hz, 3H), 1.68 (dtd, *J* = 13.2, 5.7, 1.5

Hz, 1H), 1.15 (s, 3H). $^{13}C\{^1H\}$ NMR (126 MHz, $CDCl_3$) δ 199.6, 150.7, 143.2, 140.3, 134.7, 125.9, 109.8, 74.0, 40.7, 34.2, 31.6, 20.4, 16.4. HRMS (FTMS + p ESI) m/z: $[M + H]^+$ Calculated for $C_{13}H_{17}O_3$ 221.1172; Found 221.1171. Pure crystals were obtained via recrystallization from EtOAc:heptane via slow evaporation MP: 95 – 97 °C.

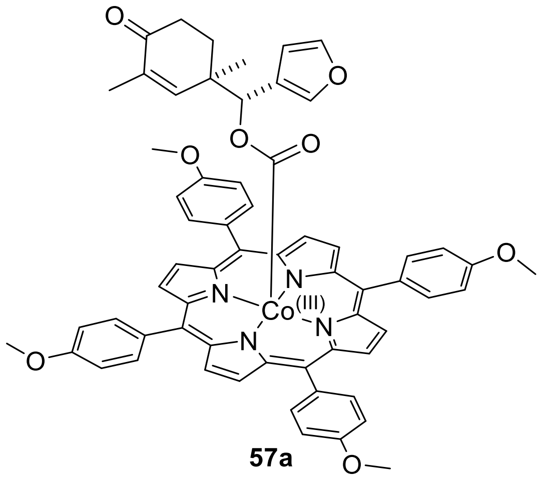

57a

±-(R)-4-((R)-furan-3-yl(oxycarbonylcobalt meso-tetraphenylporphyrin)methyl)-2,4-dimethylcyclohex-2-enone (57a). From **50a** (0.1040 g, 2.5 eq), Co(II)TAP (0.1503 g, 1 eq), and DDQ (0.0430 g, 1 eq) in DCM (15 mL, 0.0125 M) under 7 atm of CO (g) via general carbonylation procedure[xxiv] was obtained **57a** (0.0096 g, 5%) as a deep red-purple solid. ^1H NMR (600 MHz, $CDCl_3$) δ 8.95 (s, 8H), 8.01 (d, *J* = 44.9 Hz, 8H), 7.28 – 7.24 (m, 8H), 6.74 (s, 1H), 5.03 (s, 1H), 4.44 (s, 1H), 4.19 (s, 1H), 4.08 (s, 12H), 3.07 (s, 1H), 1.80 (dd, *J* = 11.9, 4.9 Hz, 1H), 1.73 (dt, *J* = 11.8, 5.4 Hz, 1H), 1.31 (s, 3H), 0.41 (td, *J* = 12.8, 12.1, 4.8 Hz, 1H), 0.30 – 0.23 (m, 1H), -0.52 (s, 3H). $^{13}C\{^1H\}$ NMR (151 MHz, $CDCl_3$) δ 198.7, 159.6, 148.4, 146.0, 146.0, 141.6, 138.5, 134.8, 134.0, 133.6, 133.0, 121.8, 120.5, 112.6, 108.4, 75.6, 57.1, 55.7, 53.6, 38.5, 33.0, 29.8, 29.1, 18.5, 16.0. HRMS (FTMS + p ESI) m/z: $[M]^+$ Calculated for $C_{62}H_{51}O_8N_4Co$ 1038.3033; Found 1038.3029

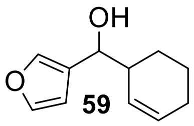

59

[xxiv] The only alteration is that this sample was purified on a neutral alumina prep plate

±-Cyclohex-2-en-1-yl(furan-3-yl)methanol (**59**). From 3-furfuraldehyde (1.1615 g, 1 eq), 3-bromo cyclohexene (1.67 mL, 1.2 eq), zinc dust (0.87 g, 1.1 eq) and NH_4Cl (aq) (24 ml) in THF (12 mL, 1 M) via General procedure B was obtained **59** (1.4527 g, 67% 74:26 d.r) as a yellow oil. ^{1}H NMR (500 MHz, $CDCl_3$) δ 7.44 – 7.36 (m, 2H), 6.42 – 6.36 (m, 1H), 5.91 – 5.80 (m, 1H), 5.79 – 5.46 (m, 1H), 4.59 – 4.47 (m, 1H), 2.47 (m, 1H), 1.99 (m, 2H), 1.84 – 1.62 (m, 3H), 1.60 – 1.33 (m, 2H). ^{13}C{^{1}H} NMR (151 MHz, $CDCl_3$) δ 143.4, 143.3, 139.9, 139.6, 130.5, 130.5, 128.2, 127.8, 127.4, 126.7, 109.0, 108.7, 70.9, 70.4, 42.1, 41.9, 26.0, 25.3, 24.3, 21.6, 21.3. HRMS (GC QTOF) m/z: $[M]^+$ Calculated for $C_{11}H_{14}O_2$ 178.0988; Found 178.0973.

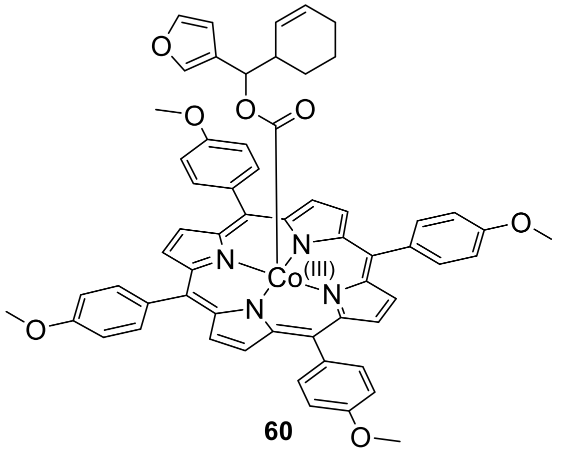

60

±-(3S,3aR)-3-(alkoxycarbonylcobaltmeso-tetraphenylporphyrin) cyclopentyl)-3a,7-dimethyl-3,3a,4,5-tetrahydroisobenzofuran-1,6-dione (60). From (**59**) (0.2813 g, 2.5 eq), Co(II)TAP (0.5000 g, 1 eq), and DDQ (0.1434 g, 1.01 eq) in DCM (50 mL, 0.0125 M) under 7 atm of CO (g) via General procedure A was obtained (**60**) (0.2032 g, 32%) as a deep red-purple foam. ^{1}H NMR (500 MHz, $CDCl_3$) δ 8.97 – 8.88 (m, 8H), 8.02 (bs, 8H), 7.25 (d, *J* = 7.4 Hz, 8H), 6.84 – 6.77 (m, 1H), 5.42 (d, *J* = 3.4 Hz, 1H), 5.32 – 5.24 (m, 2H), 5.18 – 5.13 (m, 1H), 4.62 (d, *J* = 3.3 Hz, 1H), 4.06 (s, 12H), 3.95 (d, *J* = 10.1 Hz, 1H), 3.03 (dd, *J* = 7.8, 3.6 Hz, 1H), 0.98 – 0.92 (m, 1H), 0.83 (s, 1H), 0.56 – 0.51 (m, 1H), -0.06 – -0.12 (m, 1H), -0.82 (d, *J* = 11.0 Hz, 1H). ^{13}C{^{1}H} NMR (126 MHz, $CDCl_3$) δ 159.4, 145.9, 145.9, 145.9, 141.7, 138.5, 134.7, 134.4, 132.8, 122.5, 121.5, 112.5, 107.9, 73.4, 55.7, 55.7, 39.1, 24.7, 22.8, 20.2. HRMS (FTMS + p ESI) m/z: $[M]^+$ calculated for $C_{60}H_{49}O_7N_4Co$ 996.2928; found 996.2918

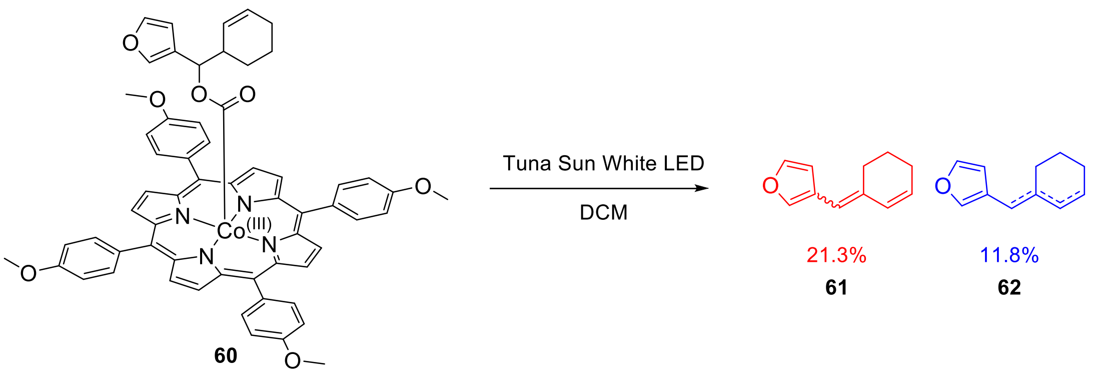

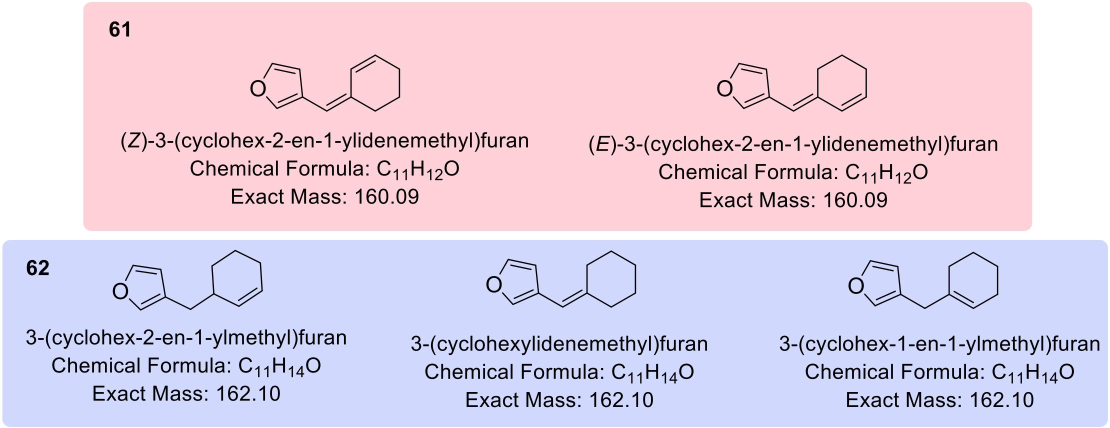

Mixture of Dienes (61-62). Co(III)TAP carbonylation adduct (**60**) (0.1089 g, 1 eq) was mixed in DCM (8.7 mL, 0.0125 M) and photoirradiated for 16 hours via general procedure D to obtain **61-62** as a colorless low boiling oil (5.9 mg, 33%) as a colorless oil. The mixture was analyzed by GC-HRMS and had relative abundances assigned based on peak area and mass.

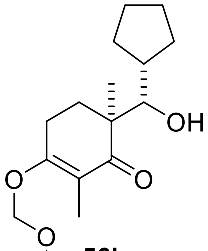

56b

±-(S)-6-((S)-cyclopentyl(hydroxy)methyl)-3-(methoxymethoxy)-2,6-dimethylcyclohex-2-en-1-one (56b). From freshly prepared 1M LDA in THF (1.02 eq), vinylogous ester **54** (2.0063 g, 1.0 eq), cyclohexyl carboxaldehyde (1.2 mL, 1.01 eq), via the general aldol procedure was obtained

56b (2.9315 g, 95% 84:16 d.r[xxv]) as a colorless oil. ^{1}H NMR (600 MHz, CDCl$_3$) δ 5.14 (q, *J* = 7.0 Hz, 2H), 4.21 (bs, 1H), 3.79 (dd, *J* = 4.3, 1.9 Hz, 1H), 3.46 (s, 3H), 2.71 – 2.56 (m, 2H), 1.96 (dt, *J* = 8.8, 4.4 Hz, 1H), 1.88 – 1.78 (m, 2H), 1.71 (s, 3H), 1.68 – 1.56 (m, 5H), 1.55 – 1.40 (m, 3H), 1.15 (s, 3H). ^{13}C{^{1}H} NMR (126 MHz, CDCl$_3$) δ 206.5, 168.5, 114.9, 92.6, 77.1, 56.7, 46.6, 41.3, 31.7, 29.8, 27.2, 26.1, 25.4, 21.8, 16.8, 7.9. HRMS (FTMS + p ESI) m/z: [M + H]$^+$ Calculated for $C_{16}H_{27}O_4$ 283.1909; Found 283.1899

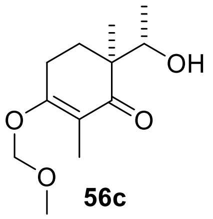

56c

±-(S)-6-((S)-1-hydroxyethyl)-3-(methoxymethoxy)-2,6-dimethylcyclohex-2-enone (56c). From freshly prepared 1M LDA in THF (1.02 eq), vinylogous ester **54** (2.0066 g, 1.0 eq)**,** acetylaldehyde[xxvi] (2.0020 g, 4 eq), via the general aldol procedure was obtained **56c** (2.2460 g, 90% 81:19 d.r) as a colorless oil. ^{1}H NMR (400 MHz, CDCl$_3$) δ 6.61 (s, 1H), 3.74 – 3.67 (m, 1H), 2.49 (dd, *J* = 8.4, 5.4 Hz, 1H), 2.19 – 1.93 (m, 1H), 1.78 (d, *J* = 1.4 Hz, 3H), 1.72 (dtd, *J* = 13.3, 5.4, 1.6 Hz, 1H), 1.51 – 1.38 (m, 1H), 1.21 (d, *J* = 6.4 Hz, 3H), 1.12 (s, 3H). ^{13}C{^{1}H} NMR (101 MHz, CDCl$_3$) δ 207.1, **204.9**, 169.0, **168.6**, **115.8**, 114.9, 92.6, **92.6**, **71.9**, 71.4, 56.7, **56.7**, **47.5**, 45.4, 29.8, **26.8**, **21.8**, 21.7, **19.7**, **17.0**, 16.4, **14.2**, 14.1, **7.8**, 7.8.[xxvii] HRMS (FTMS + p ESI) m/z: [M]$^+$ Calculated for $C_{12}H_{20}O_4$ 228.1367; Found 228.1361

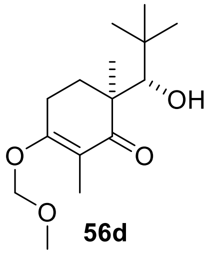

56d

±-(S)-6-((S)-1-hydroxy-2,2-dimethylpropyl)-3-(methoxymethoxy)-2,6-dimethylcyclohex-2-enone (56d). From freshly prepared 1M LDA in THF (1.02 eq), vinylogous ester **54** (1.0631 g, 1.0

[xxv] Diastereomers could be separated by skimming fractions from a gradient column going from 0% EtOAc in hexanes to 30% EtOAc very slowly. The minor diastereomer eluted first.

[xxvi] Due to acetaldehyde's low boiling point, it was transferred into a tared 7 ml vial equipped with as septum cap via glass pipet, and was cannulated into the reaction mixture from its own pressure.

[xxvii] All signals in bold are associated with the minor diastereomer

eq), pivaldehyde (0.78 mL, 1.05 eq), via the general aldol procedure was obtained **56d** (0.4866 g, 30% >95:5 d.r) as a colorless oil. ^{1}H NMR (400 MHz, $CDCl_3$) δ 5.13 (s, 2H), 3.71 (d, *J* = 4.1 Hz, 1H), 3.46 (s, 3H), 3.39 (d, *J* = 4.2 Hz, 1H), 2.68 (dtt, *J* = 17.9, 4.1, 1.3 Hz, 1H), 2.63 – 2.50 (m, 1H), 2.09 (ddd, *J* = 13.2, 10.5, 5.5 Hz, 1H), 1.96 (ddd, *J* = 13.3, 5.1, 4.1 Hz, 1H), 1.72 (dd, *J* = 1.9, 1.3 Hz, 3H), 1.21 (s, 3H), 1.00 (s, 9H). $^{13}C\{^{1}H\}$ NMR (101 MHz, $CDCl_3$) δ 205.0, 167.7, 114.7, 92.6, 80.5, 56.7, 48.6, 36.9, 29.6, 28.7, 22.0, 19.0, 8.1. HRMS (GC QTOF) m/z: $[M]^+$ Calculated for $C_{15}H_{26}O_4$ 270.1830; Found 270.1824

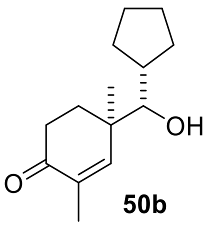

50b

±-(R)-4-((S)-cyclopentyl(hydroxy)methyl)-2,4-dimethylcyclohex-2-en-1-one (50b). From vinylogous ester **56b** (0.9734 g, 1 eq) in toluene (7mL, 0.5 M), DIBAL (2.45 mL, 4 eq) according to general Stork-Danheiser Procedure to give enone **50b** (0.4941 g, 64% >95:5 d.r) as a colorless oil. ^{1}H NMR (500 MHz, $CDCl_3$) δ 6.58 (s, 1H), 3.46 (d, *J* = 5.3 Hz, 1H), 2.56 – 2.42 (m, 2H), 2.14 – 2.02 (m, 2H), 1.84 – 1.67 (m, 3H), 1.76 (s, 3H), 1.67 – 1.37 (m, 6H), 1.35 – 1.24 (m, 1H), 1.17 (s, 3H). $^{13}C\{^{1}H\}$ NMR (126 MHz, $CDCl_3$) δ 199.9, 152.2, 133.6, 81.6, 42.6, 41.4, 34.4, 31.9, 31.0, 28.4, 25.5, 25.5, 21.8, 16.4. HRMS (FTMS + p ESI) m/z: $[M + H]^+$ Calculated for $C_{14}H_{23}O_2$ 223.1698; Found 223.1691

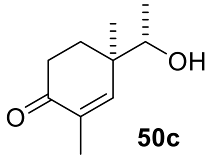

50c

±-(R)-4-((S)-1-hydroxyethyl)-2,4-dimethylcyclohex-2-enone (50c).

From vinylogous ester **56c** (0.9985 g, 1eq) in toluene (9 mL, 0.5 M), DIBAL (2.34 mL, 3 eq) according to general Stork-Danheiser Procedure to give enone **50c** (0.4941 g, 64% >95:5 d.r) as a colorless oil. ^{1}H NMR (400 MHz, $CDCl_3$) δ 6.61 (s, 1H), 3.74 – 3.67 (m, 1H), 2.49 (dd, *J* = 8.4, 5.4 Hz, 1H), 2.19 – 1.93 (m, 1H), 1.78 (d, *J* = 1.4 Hz, 3H), 1.72 (dtd, *J* = 13.3, 5.4, 1.6 Hz, 1H), 1.51 – 1.38 (m, 1H), 1.21 (d, *J* = 6.4 Hz, 3H), 1.12 (s, 3H). (^{13}CNMR of a different sample with d.r. 5:1) $^{13}C\{^{1}H\}$ NMR (101 MHz, $CDCl_3$) δ 199.8, **151.8**, 151.1, 135.2, **134.4**, 77.5, 77.2, 76.8, 74.1,

73.2, **41.0**, 40.5, 34.2, **34.1**, 30.7, **29.3**, **21.2**, 19.8, 18.4, **17.9**, 16.4, **14.3**.[xxviii] HRMS (FTMS + p ESI) m/z: $[M + H]^+$ Calculated for $C_{10}H_{17}O_2$ 169.1223; Found 169.1222

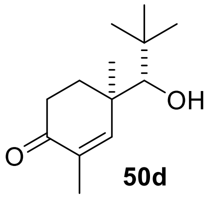

50d

±-(R)-4-((S)-1-hydroxy-2,2-dimethylpropyl)-2,4-dimethylcyclohex-2-enone (50d). From vinylogous ester **56d** (0.9985 g, 1eq) in toluene (9 mL, 0.5 M), DIBAL (2.34 mL, 3 eq) according to general Stork-Danheiser Procedure to give enone **50d** (0.3681 g, 91% >95:5 d.r) as a colorless crystalline solid. ^{1}H NMR (500 MHz, $CDCl_3$) δ 6.66 (s, 1H), 3.26 (d, *J* = 5.6 Hz, 1H), 2.53 – 2.45 (m, 2H), 2.37 – 2.25 (m, 1H), 1.88 (dtd, *J* = 13.4, 4.9, 1.7 Hz, 1H), 1.76 (d, *J* = 1.4 Hz, 3H), 1.65 (d, *J* = 6.1 Hz, 1H), 1.27 (s, 3H), 1.04 (s, 9H). $^{13}C\{^1H\}$ NMR (101 MHz, CDCl3) δ 199.9, 153.8, 132.1, 85.0, 42.8, 37.6, 34.6, 30.9, 28.8, 23.8, 16.3. HRMS (FTMS + p ESI) m/z: $[M + H]^+$ Calculated for $C_{13}H_{23}O_2$ 211.1693; Found 211.1692 Pure crystals were obtained via recrystallization from EtOAc:heptane via slow evaporation MP: 55 – 57 °C.

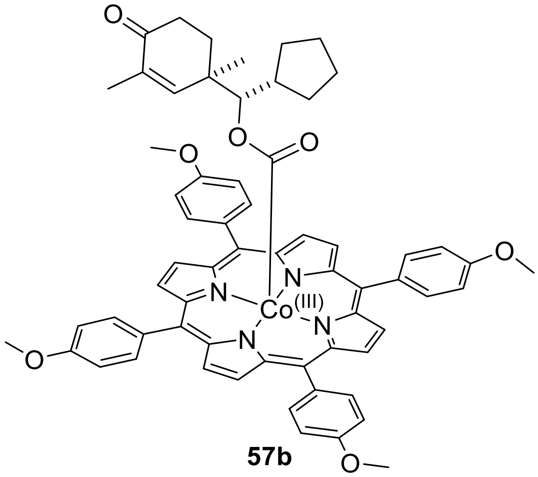

57b

±-(R)-4-((S)-cyclopentyl(oxycarbonylcobalt meso-tetraphenylporphyrin)methyl)-2,4-dimethylcyclohex-2-enone (57b). From **50b** (0.2106 g, 2.5 eq), Co(II)TAP (0.3149 g, 1 eq), and DDQ (0.0948 g, 1 eq) in DCM (30 mL, 0.0125 M) under 7 atm of CO (g) via the general carbonylation procedure was obtained **57b** (0.0548 g, 15%) as a deep red-purple foam. ^{1}H NMR (500 MHz, $CDCl_3$) δ 8.94 (s, 8H), 8.04 (s, 8H), 7.29 – 7.23 (m, 8H), 4.27 (s, 1H), 4.06 (s, 12H),

[xxviii] All peaks in bold are associated with minor diastereomer

2.41 (d, J = 6.0 Hz, 1H), 1.94 – 1.81 (m, 2H), 1.20 (s, 3H), 1.01 (d, J = 6.7 Hz, 1H), 0.79 (ddt, J = 15.7, 7.5, 4.3 Hz, 4H), 0.61 (ddd, J = 13.1, 10.8, 6.5 Hz, 1H), 0.44 – 0.38 (m, 1H), 0.30 (td, J = 10.5, 6.7 Hz, 1H), 0.01 (dt, J = 13.3, 4.8 Hz, 1H), -0.48 – -0.56 (m, 1H), -0.90 (s, 3H), -1.28 – -1.37 (m, 1H). $^{13}C\{^1H\}$ NMR (101 MHz, $CDCl_3$) δ 199.2, 162.4, 159.4, 149.9, 149.7, 146.1, 142.4, 134.3, 132.8, 132.8, 132.3, 121.7, 116.0, 112.4, 83.3, 57.1, 55.7, 40.2, 39.1, 33.2, 29.9, 29.7, 28.4, 26.4, 24.1, 23.7, 19.5, 15.8. HRMS (FTMS + p ESI) m/z: $[M]^+$ Calculated for $C_{63}H_{57}O_7N_4Co$ 1040.3554; Found 1040.3553

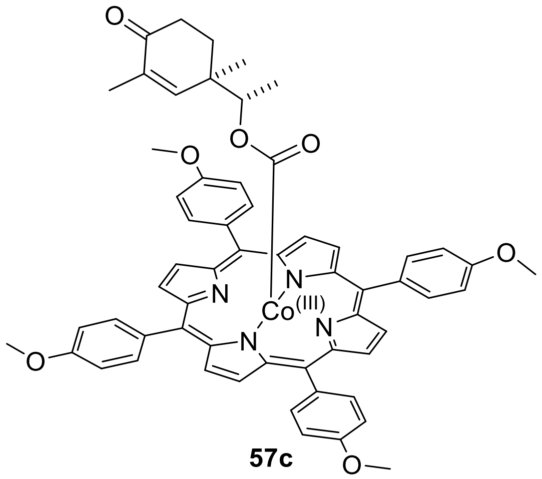

57c

±-(R)-4-((S)-1-(alkoxycarbonylcobaltmeso-tetraphenylporphyrin)ethyl)-2,4-dimethylcyclohex-2-enone (57c). From **50c** (0.2685 g, 2.5 eq), Co(II)TAP (0.5055 g, 1 eq), and DDQ (0.1449 g, 1 eq) in DCM (50 mL, 0.0125 M) under 10 atm of CO (g) via general carbonylation procedure was obtained **57c** (0.2168 g, 34%) as a deep red-purple foam. ^1H NMR (400 MHz, $CDCl_3$) δ 8.91 (s, 8H), 8.02 (d, J = 7.3 Hz, 8H), 7.26 (d, J = 8.9 Hz, 8H), 4.08 (s, 12H), 3.98 (s, 1H), 2.30 – 2.19 (m, 1H), 1.90 – 1.70 (m, 2H), 1.25 (d, J = 1.3 Hz, 3H), 0.50 – 0.39 (m, 1H), 0.38 – 0.28 (m, 1H), -0.55 (s, 3H), -1.02 (d, J = 6.3 Hz, 3H). $^{13}C\{^1H\}$ NMR (126 MHz, $CDCl_3$) δ 198.8, 159.4, 149.0, 145.7, 134.8, 134.0, 133.0, 133.0, 133.0, 121.3, 112.6, 76.5, 55.7, 53.6, 37.8, 33.0, 29.8, 29.1, 17.8, 15.8, 12.9. HRMS (FTMS + p ESI) m/z: $[M]^+$ Calculated for $C_{59}H_{51}O_7N_4Co$ 986.3084; Found 986.3084

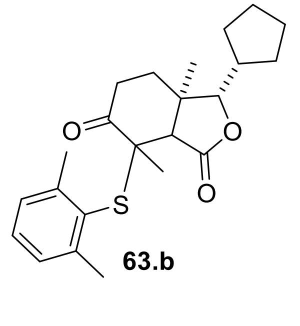

63.b

±-(3S,3aR)-3-cyclopentyl-7-((2,6-dimethylphenyl)thio)-3a,7-dimethylhexahydroisobenzofuran-1,6-dione (63b). Co(III)TAP carbonylation adduct (**57b**) (0.0.0548 g, 1 eq) and 2,6-dimethylthiophenol (0.014 mL, 2 eq) was mixed in DCM (2.9 mL, 0.0125 M) and photoirradiated for 16 hours via general procedure D to obtain **63b** as a colorless oil (2.9 mg, 14%). ^{1}H NMR (500 MHz, $CDCl_3$) δ 7.17 (t, 1H), 7.13 – 7.08 (m, 2H), 3.96 (d, J = 8.1 Hz, 1H), 3.28 – 3.21 (m, 1H), 3.18 (s, 1H), 2.51 – 2.40 (m, 6H), 2.19 – 2.07 (m, 1H), 2.01 – 1.89 (m, 2H), 1.87 – 1.78 (m, 1H), 1.86 – 1.51 (m, 5H), 1.46 (s, 3H), 1.42 – 1.32 (m, 1H), 1.34 – 1.19 (m, 2H), 1.24 (s, 3H). ^{13}C{^{1}H} NMR (101 MHz, $CDCl_3$) δ 204.7, 173.5, 146.2, 130.2, 128.7, 128.5, 88.1, 60.4, 55.5, 42.1, 41.0, 33.8, 32.1, 30.6, 29.6, 25.0, 23.6, 18.2. HRMS (FTMS + p ESI) m/z: $[M+H]^+$ Calculated for $C_{23}H_{31}O_3S$ 387.1988; Found 387.1990.

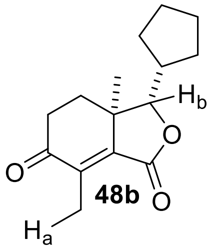

±-(3S,3aR)-3-cyclopentyl-3a,7-dimethyl-3,3a,4,5-tetrahydroisobenzofuran-1,6-dione (48b) 1 mL of DBU was added to Sulfide **63b** (0.0029 g, 1 eq) and was allowed to react overnight. The reaction mixture was partitioned between NH_4Cl and DCM and was extracted 3 times from the NH_4Cl layer with DCM. The eluent was concentrated *in vacuo*, then taken up in 1 ml of DCM and ran through a silica plug in a pipet. This was also concentrated and gave material that was examined without further purification. The mass was too small to measure on our balances. ^{1}HNMR evidence included the upfield shift of the doublet for lactone H_b from 3.9 ppm in **63b** to 3.7 ppm for **48b**. The evidence for the double bond assignment was the methyl singlet for H_a that moved downfield from 1.5 ppm in **63b** to 2.2 ppm for **48b.** HRMS (GC QTOF) m/z: $[M]^+$ Calculated for $C_{15}H_{20}O_3$ 248.1413; Found 248.1408.

References

[1] S. Blumberg, S. F. Martin, *Tetrahedron Letters* **2015**, *56*, 3674-3678.

[2] D. R. Chambers, A. Juneau, C. T. Ludwig, M. Frenette, D. B. C. Martin, *Organometallics* **2019**, *38*, 4570-4577.

[3] A. Theodorou, I. Triandafillidi, C. G. Kokotos, *European Journal of Organic Chemistry* **2017**, *2017*, 1502-1509.

[4] S.-i. Sumida, M. Ohga, J. Mitani, J. Nokami, *Journal of the American Chemical Society* **2000**, *122*, 1310-1313.

[5] F. Fu, K. L. M. Hoang, T.-P. Loh, *Organic Letters* **2008**, *10*, 3437-3439.

[6] Z. Shi, C. Shen, K. Dong, *Chemistry – A European Journal* **2021**, *27*, 18039-18042.

[7] A. Fürstner, N. Shi, *Journal of the American Chemical Society* **1996**, *118*, 2533-2534.

[8] T. Neveselý, J. J. Molloy, C. McLaughlin, L. Brüss, C. G. Daniliuc, R. Gilmour, *Angewandte Chemie International Edition* **2022**, *61*.

[9] I. Triandafillidi, M. G. Kokotou, C. G. Kokotos, *Organic Letters* **2018**, *20*, 36-39.

[10] T. R. Hoye, P. R. Hanson, *The Journal of Organic Chemistry* **1991**, *56*, 5092-5095.

[11] Y. Tanabe, N. Ohno, *The Journal of Organic Chemistry* **1988**, *53*, 1560-1563.

[12] S. W. M. Crossley, F. Barabé, R. A. Shenvi, *Journal of the American Chemical Society* **2014**, *136*, 16788-16791.

[13] Q. Yang, M. Sheng, J. J. Henkelis, S. Tu, E. Wiensch, H. Zhang, Y. Zhang, C. Tucker, D. E. Ejeh, *Org. Process Res. Dev.* **2019**, *23*, 2210-2217.

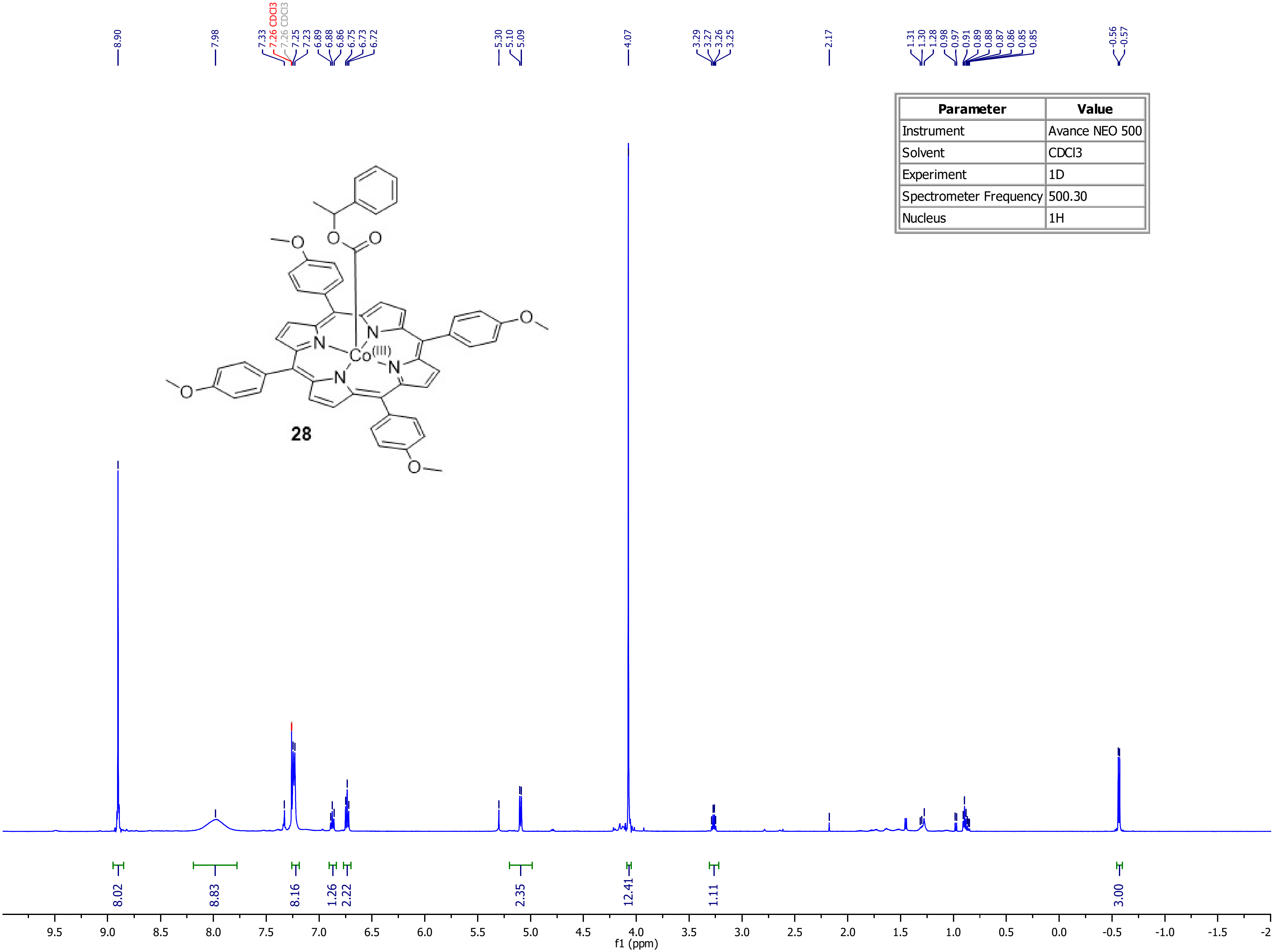

Parameter	Value
Instrument	Avance NEO 500
Solvent	CDCl3
Experiment	1D
Spectrometer Frequency	500.30
Nucleus	1H

JNH_04_206.2.fid — 2-phenyl ethanol TAP carbonylation for Dana verification

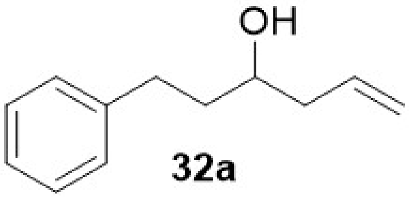

32a

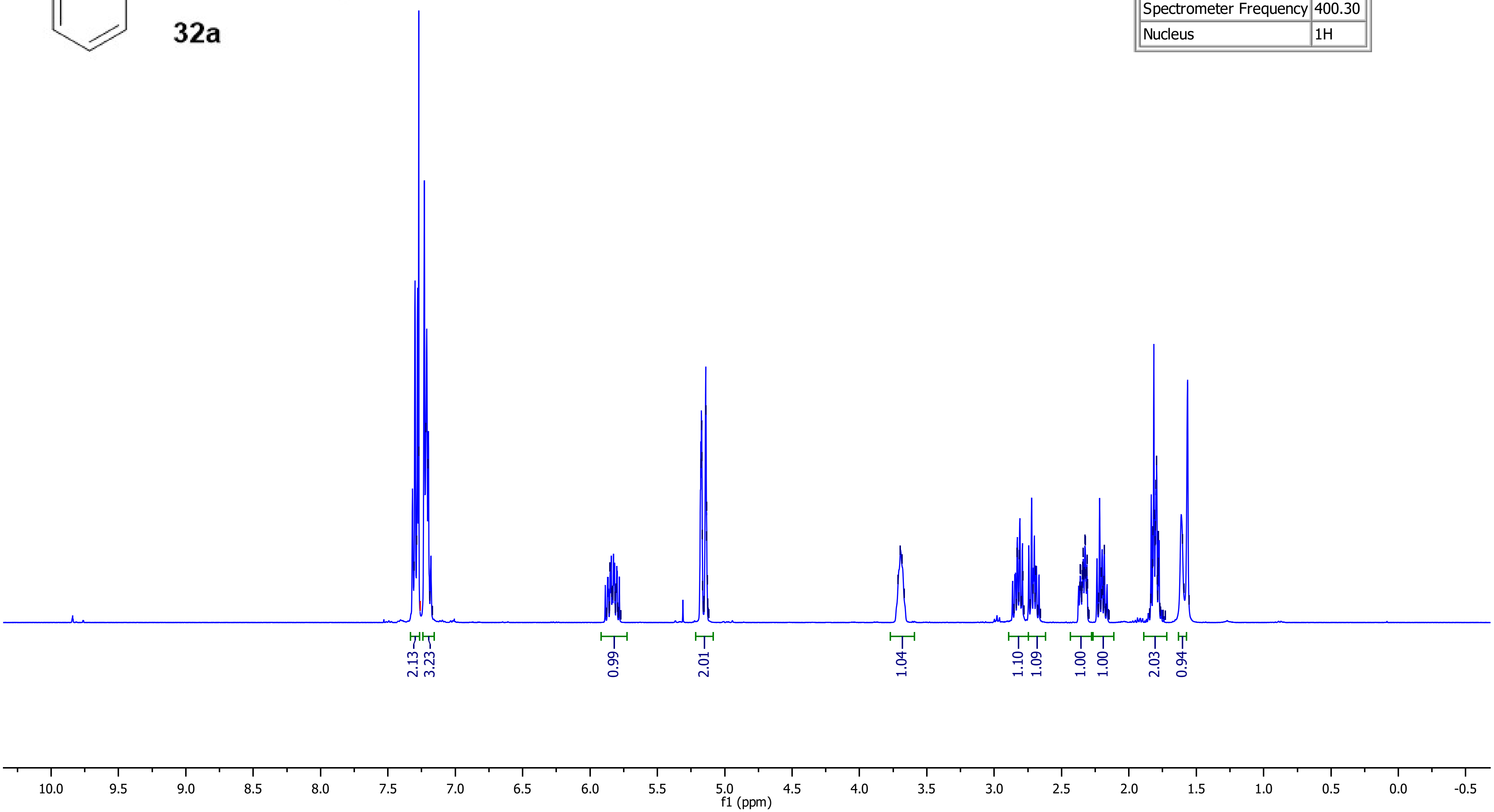

Parameter	Value
Instrument	Avance
Solvent	CDCl3
Experiment	1D
Spectrometer Frequency	400.30
Nucleus	1H

TKB_01_35.2.fid — Barbeir crude raction mixture after silica plug and high vac 8/12/2024

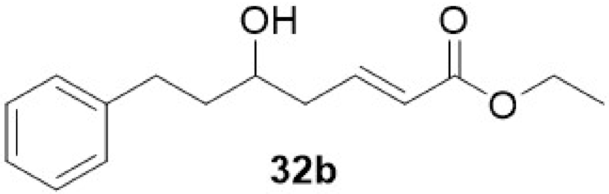

32b

Parameter	Value
Instrument	spect
Solvent	CDCl3
Experiment	1D
Spectrometer Frequency	600.21
Nucleus	1H

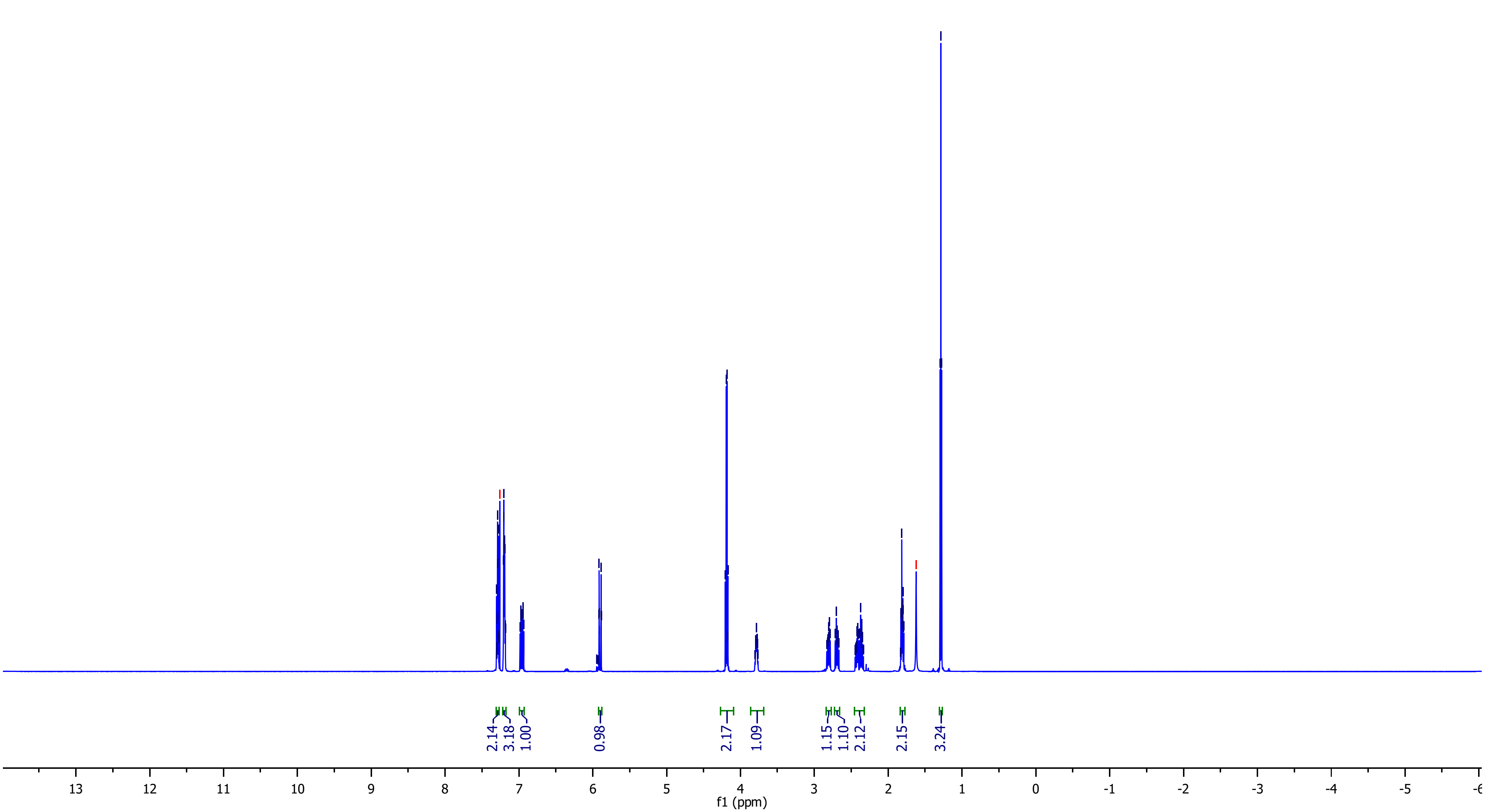

JNH_04_30.2.fid — Ethyl acrylate grubbs product logan

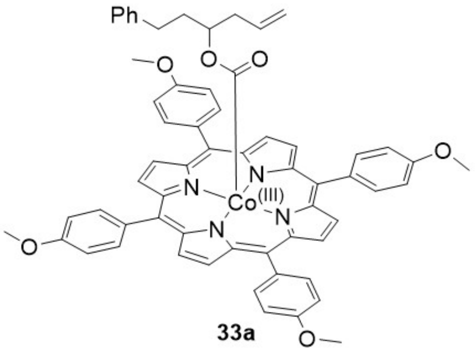

33a

Parameter	Value
Instrument	Avance
Solvent	CDCl3
Experiment	1D
Spectrometer Frequency	400.30
Nucleus	1H

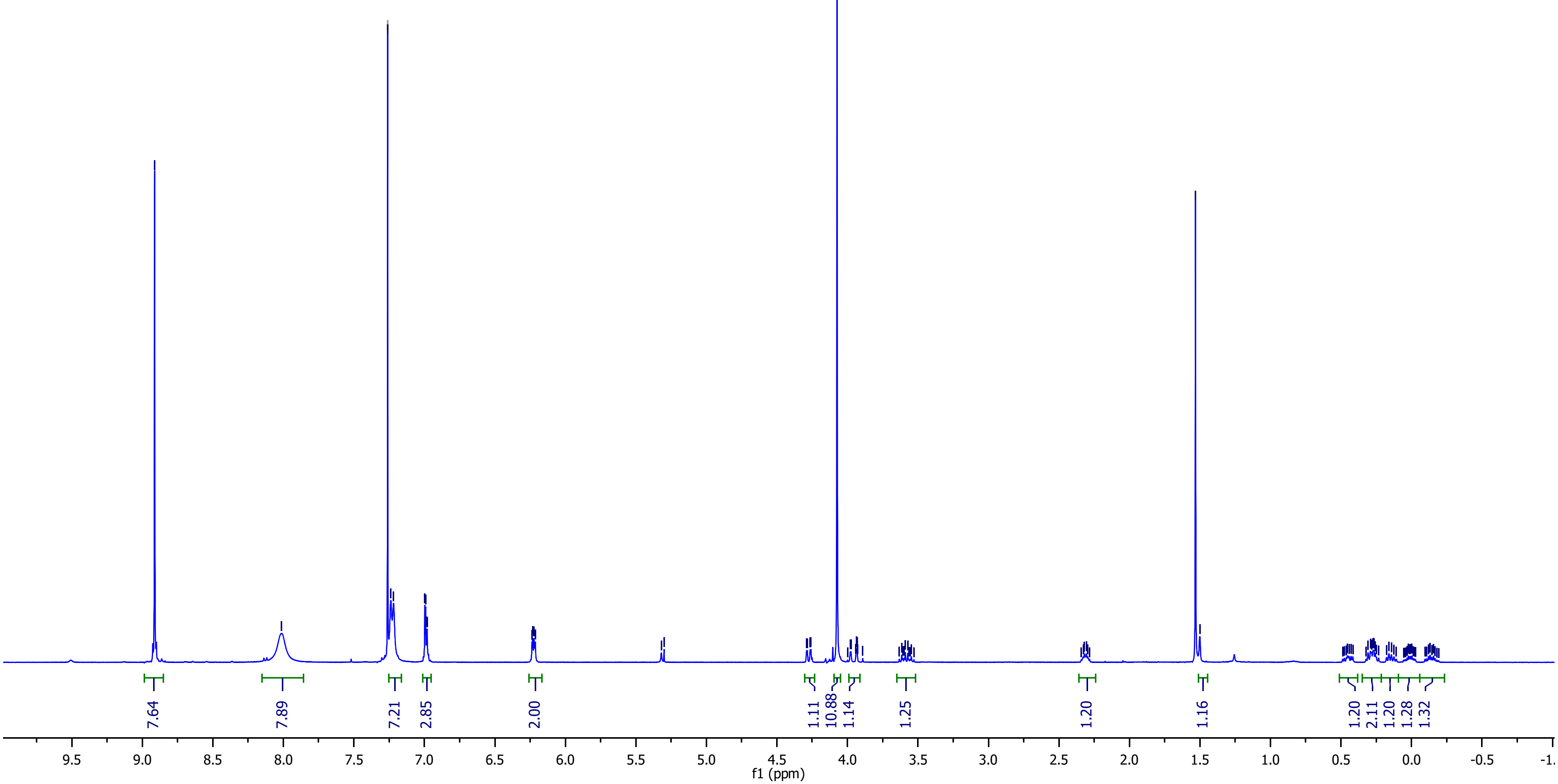

JNH_04_84.1.fid — Cory carbonylation terminal alkene model

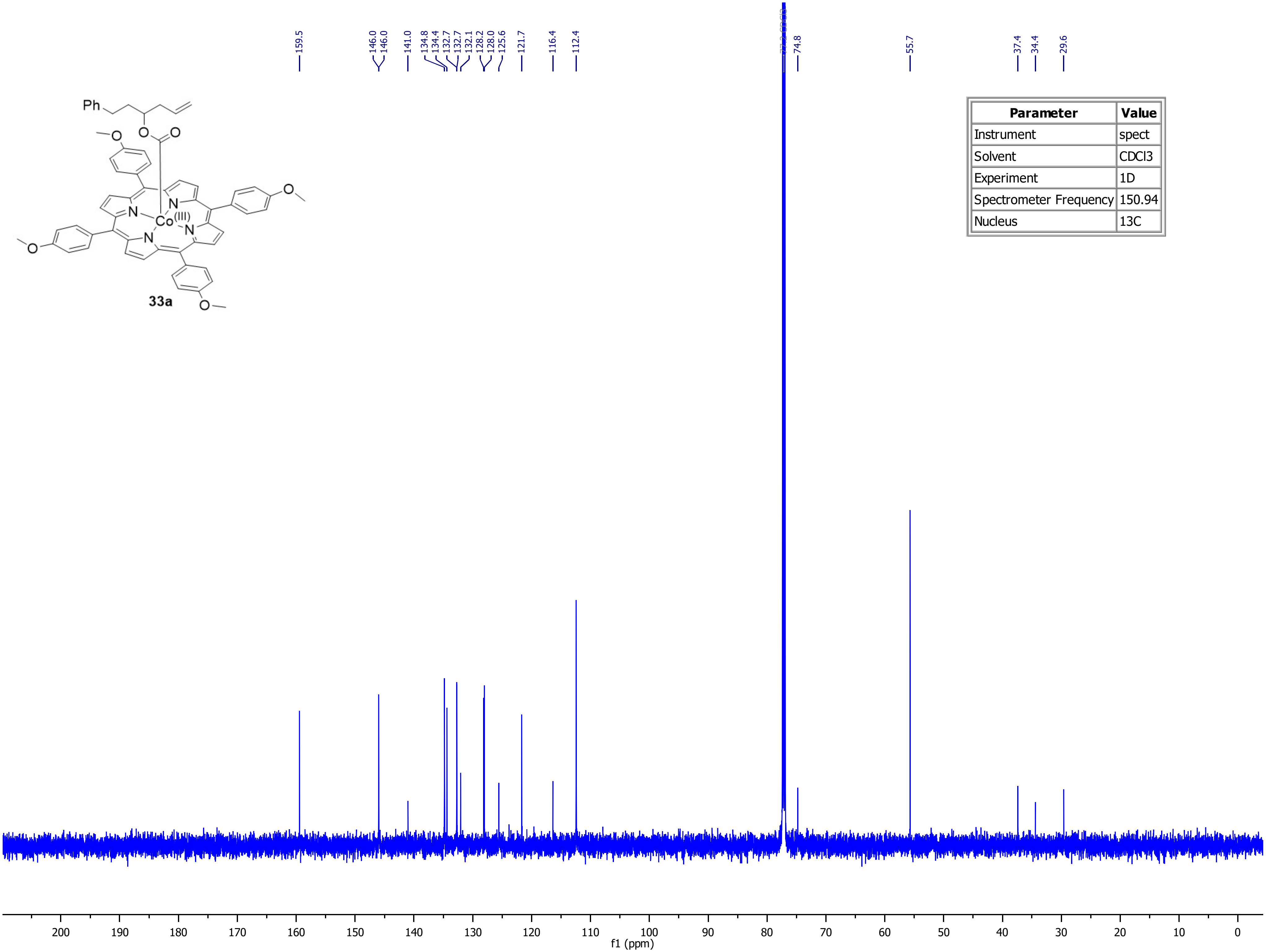

Parameter	Value
Instrument	spect
Solvent	CDCl3
Experiment	1D
Spectrometer Frequency	150.94
Nucleus	13C

JNH_04_84 13C.1.fid — Cory terminal alkene carbonylation product

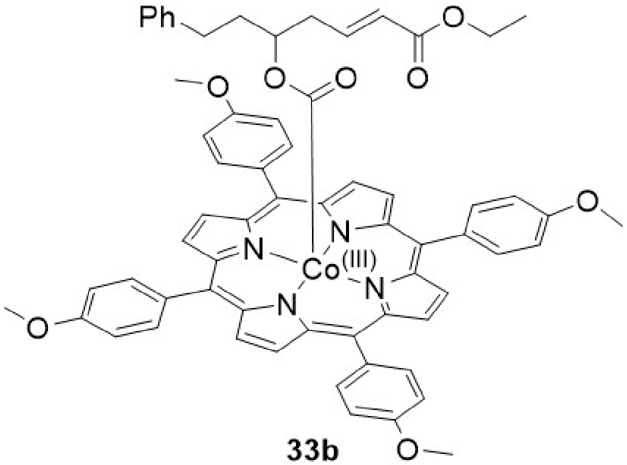

33b

Parameter	Value
Instrument	Avance
Solvent	CDCl3
Experiment	1D
Spectrometer Frequency	400.30
Nucleus	1H

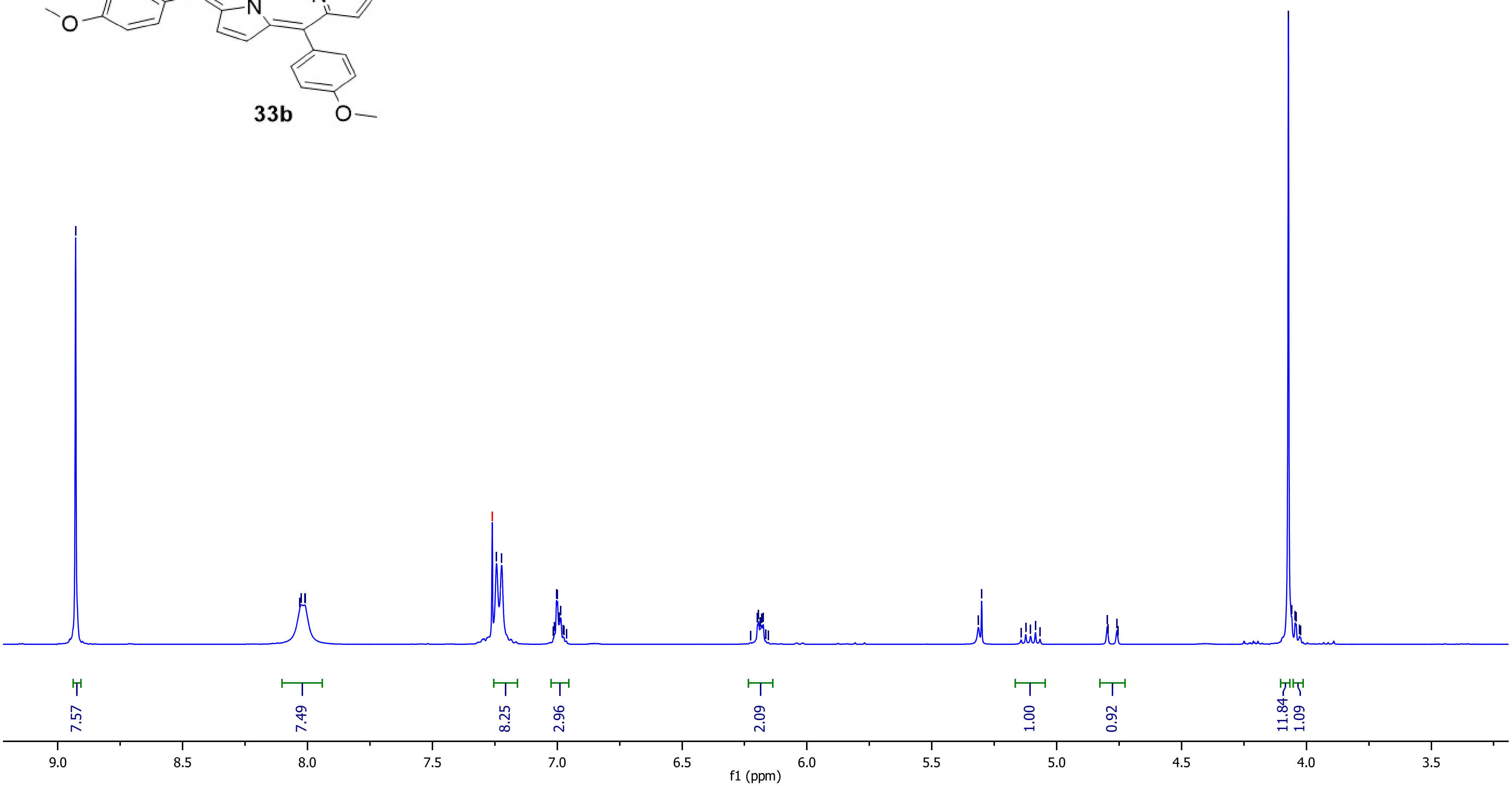

JNH_04_83.2.fid — Giese Model carbonylation Co (III) TAP (0.55) more concentrated

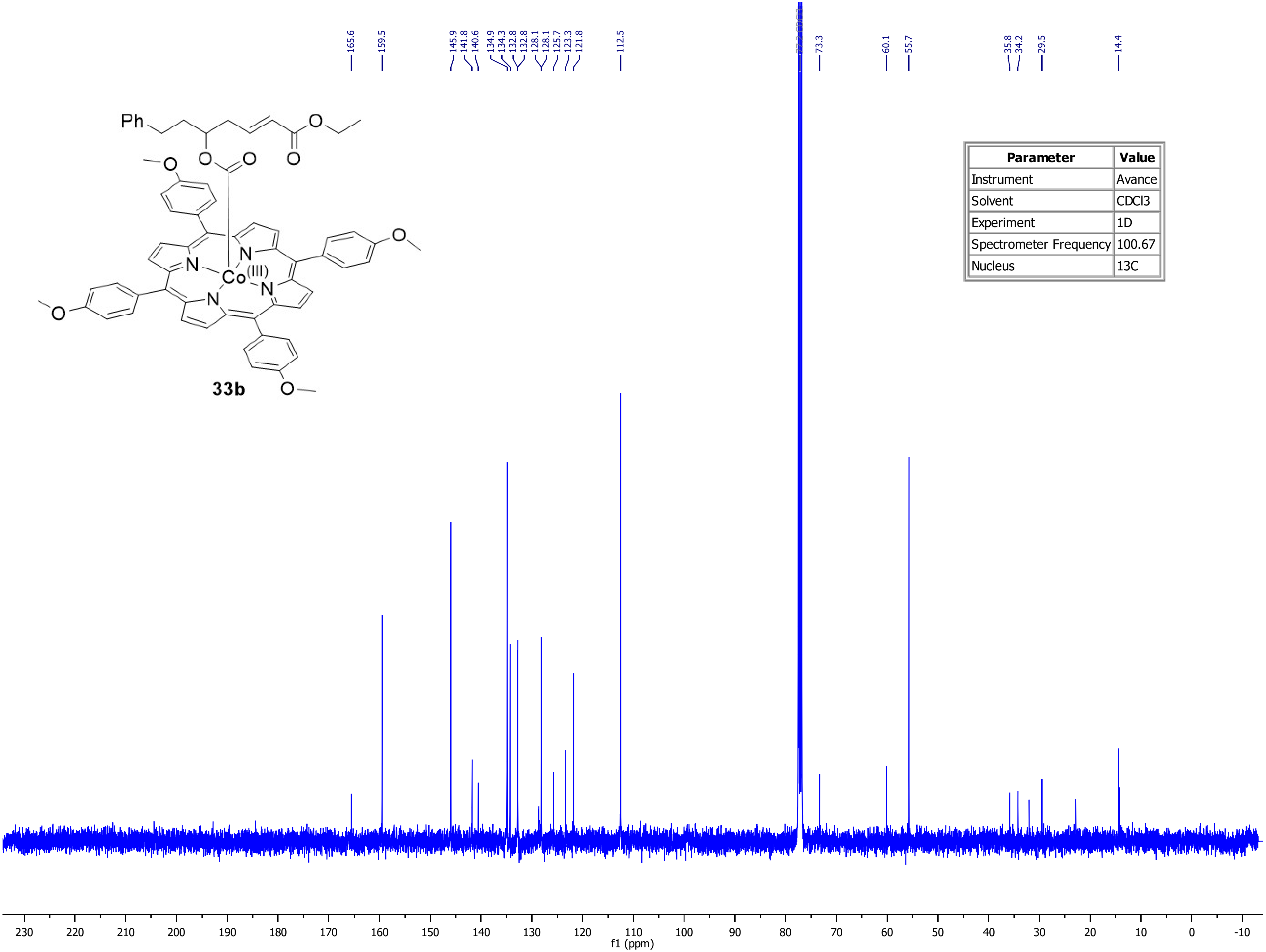

Parameter	Value
Instrument	Avance
Solvent	CDCl3
Experiment	1D
Spectrometer Frequency	100.67
Nucleus	13C

JNH_04_83 13C.2.fid — Giese Model carbonylation Co (III) TAP (0.55 g) more concentrated

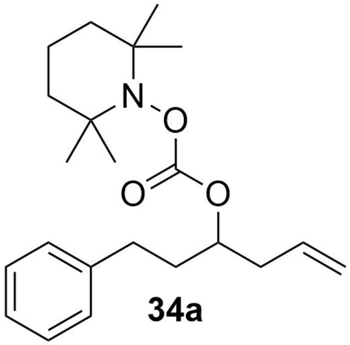

34a

Parameter	Value
Instrument	Avance
Solvent	CDCl3
Experiment	1D
Spectrometer Frequency	400.30
Nucleus	1H

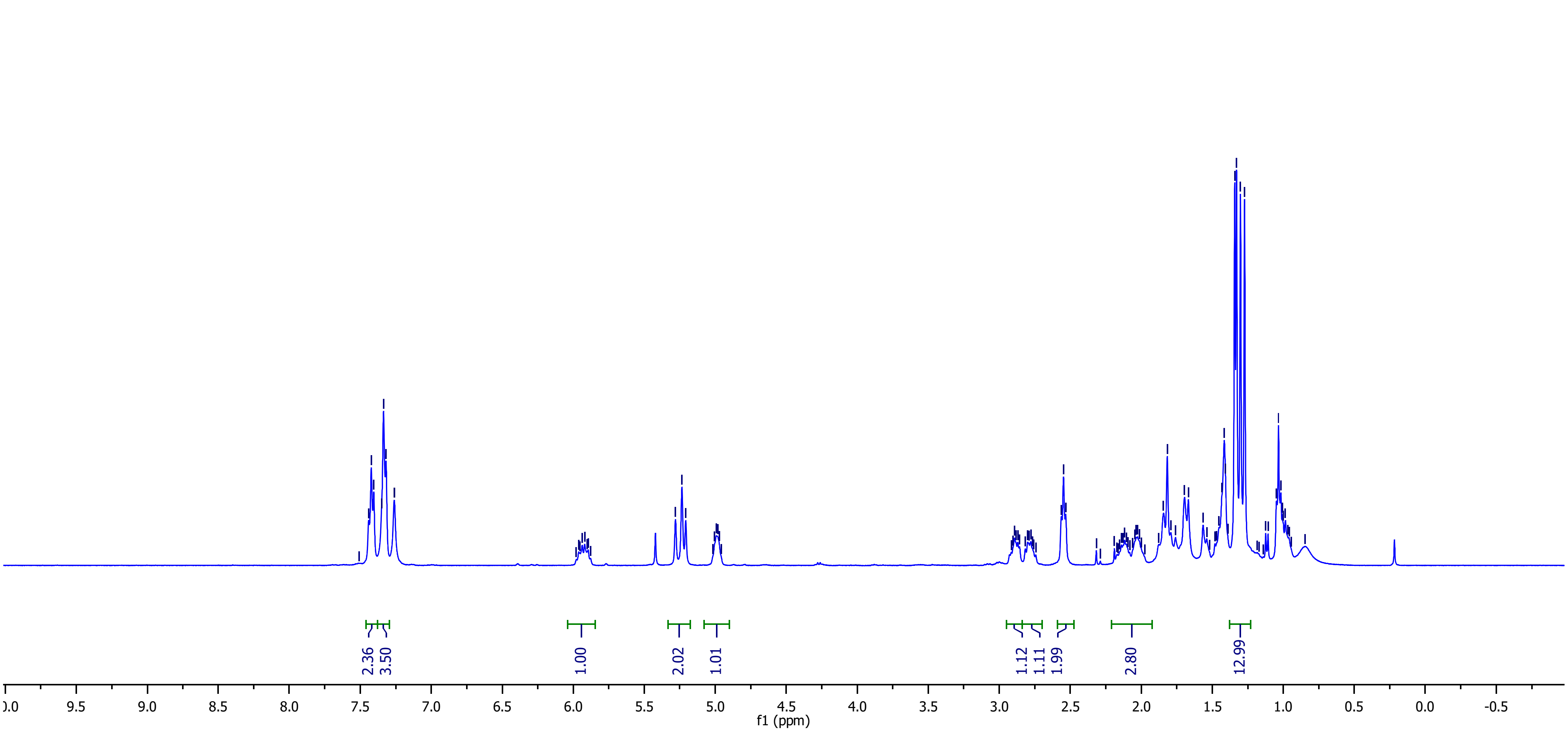

JNH_04_160.1.fid — Terminal carbonylated Co(III)TAP TEMPO trap major UV active spot (11.8 mg)

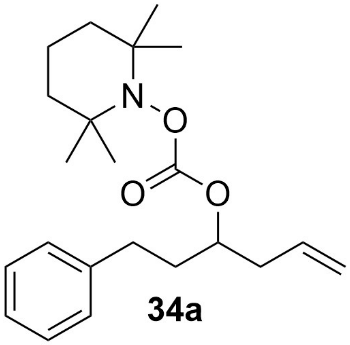

34a

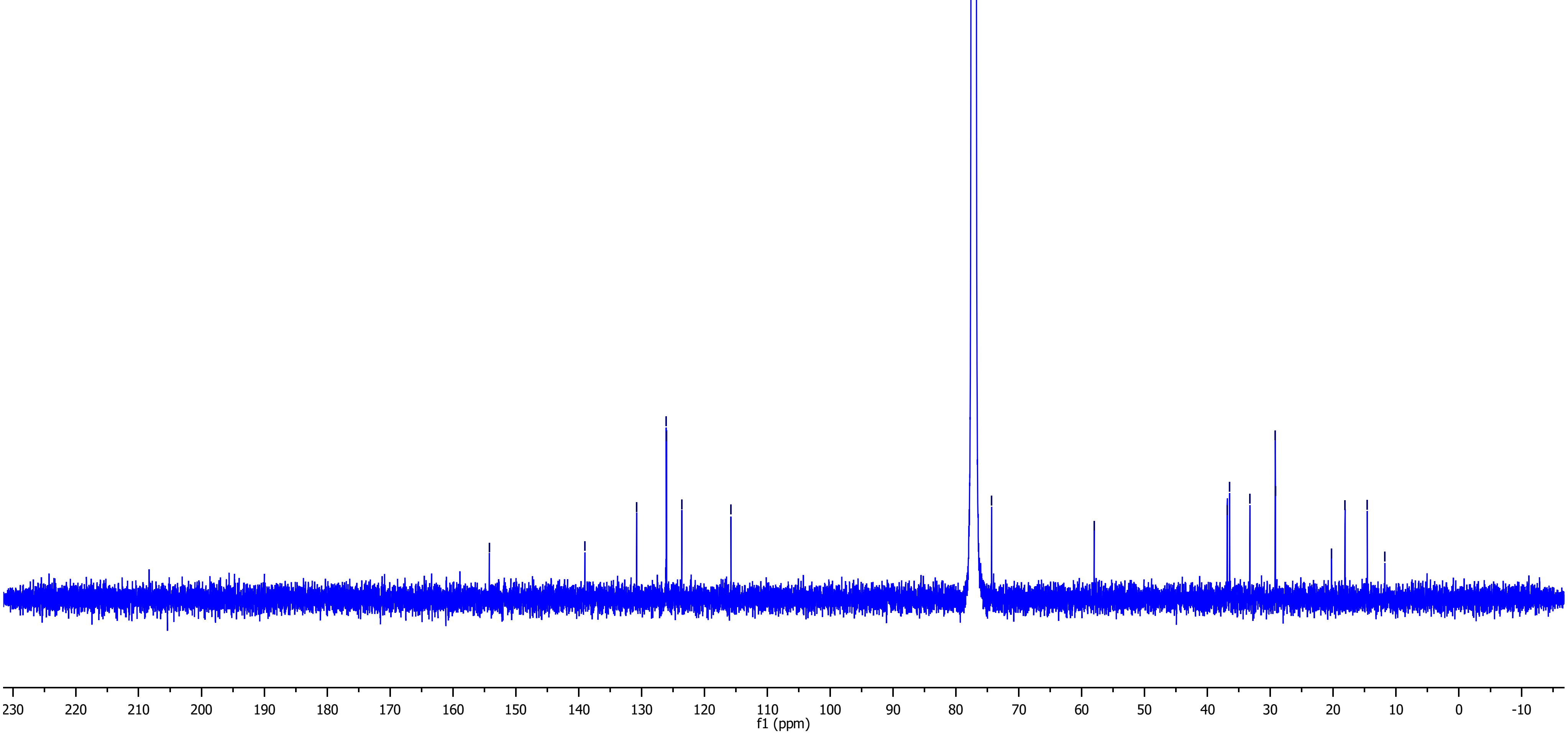

Parameter	Value
Instrument	Avance
Solvent	CDCl3
Experiment	1D
Spectrometer Frequency	100.67
Nucleus	13C

JNH_04_160 13C.1.fid — Terminal carbonylated Co(III)TAP TEMPO trap major UV active spot (11.8 mg)

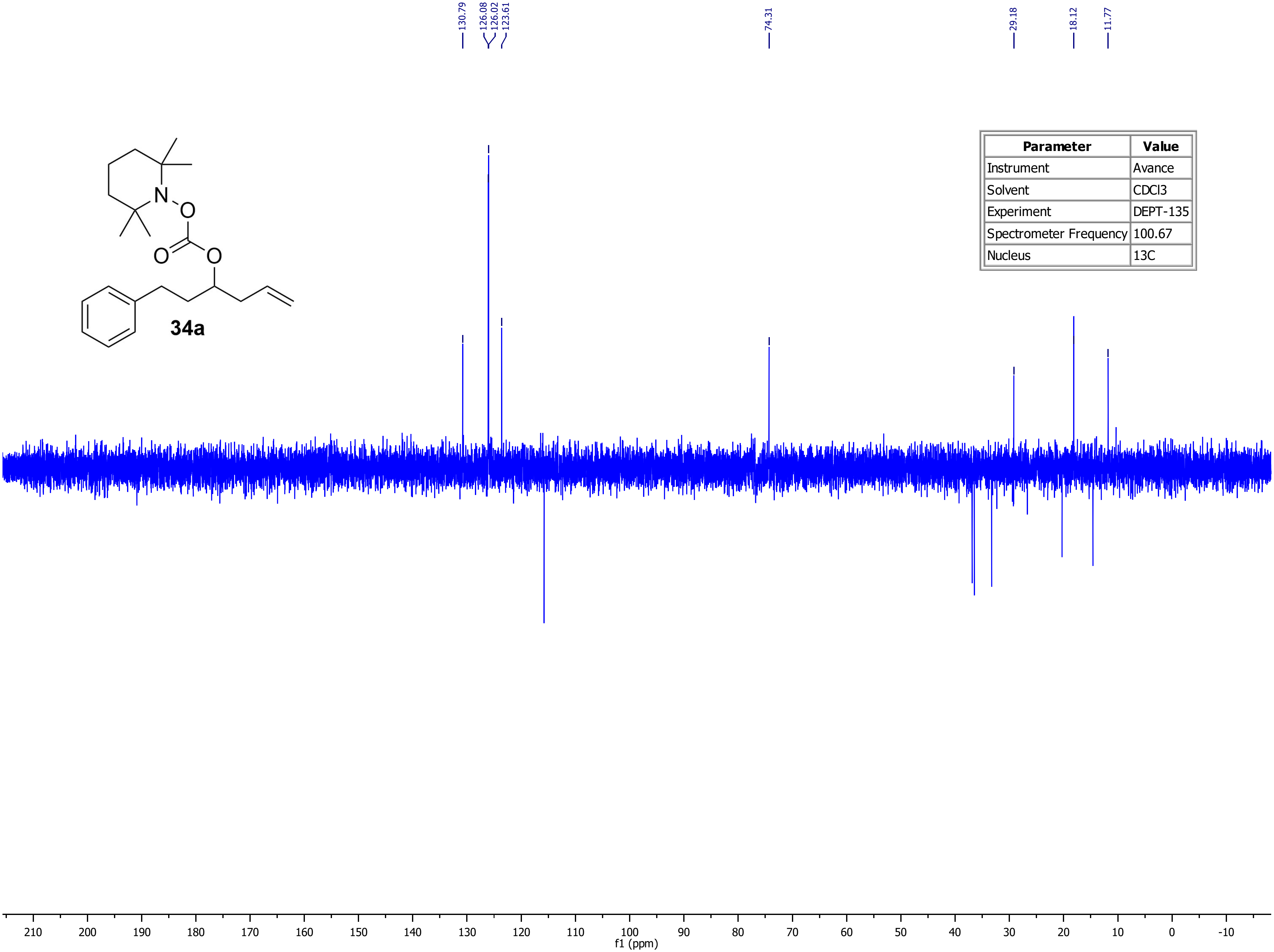

Parameter	Value
Instrument	Avance
Solvent	CDCl3
Experiment	DEPT-135
Spectrometer Frequency	100.67
Nucleus	13C

JNH_04_160 13C-DEPT135.1.fid — DEPT 135 Terminal carbonylated Co(III)TAP TEMPO trap major UV active spot (11.8 mg)

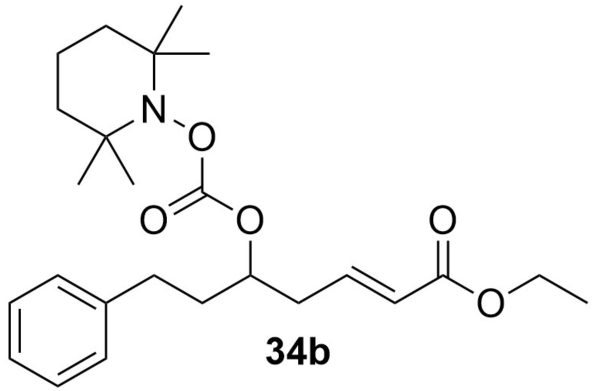

34b

Parameter	Value
Instrument	Avance
Solvent	CDCl3
Experiment	1D
Spectrometer Frequency	400.30
Nucleus	1H

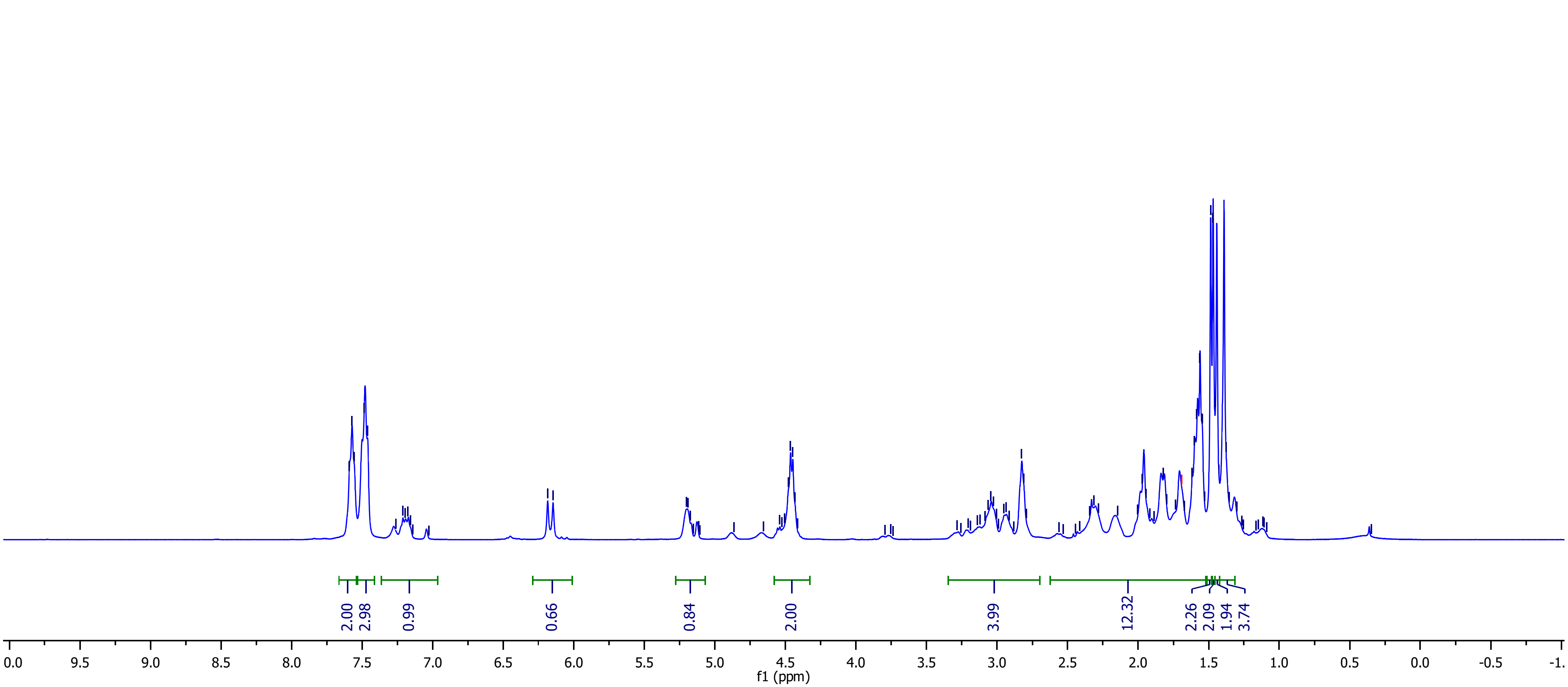

JNH_04_161.1.fid — giese carbonylated Co(III)TAP TEMPO trap major UV active spot (35.2 mg)

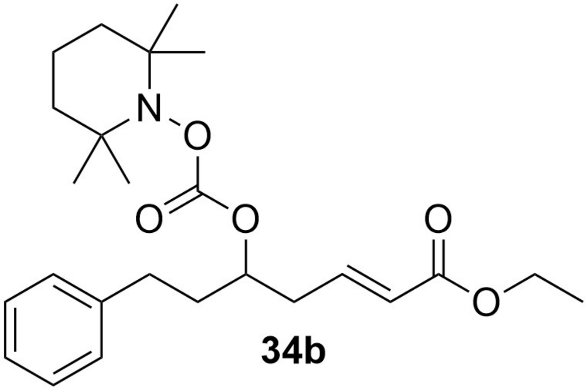

34b

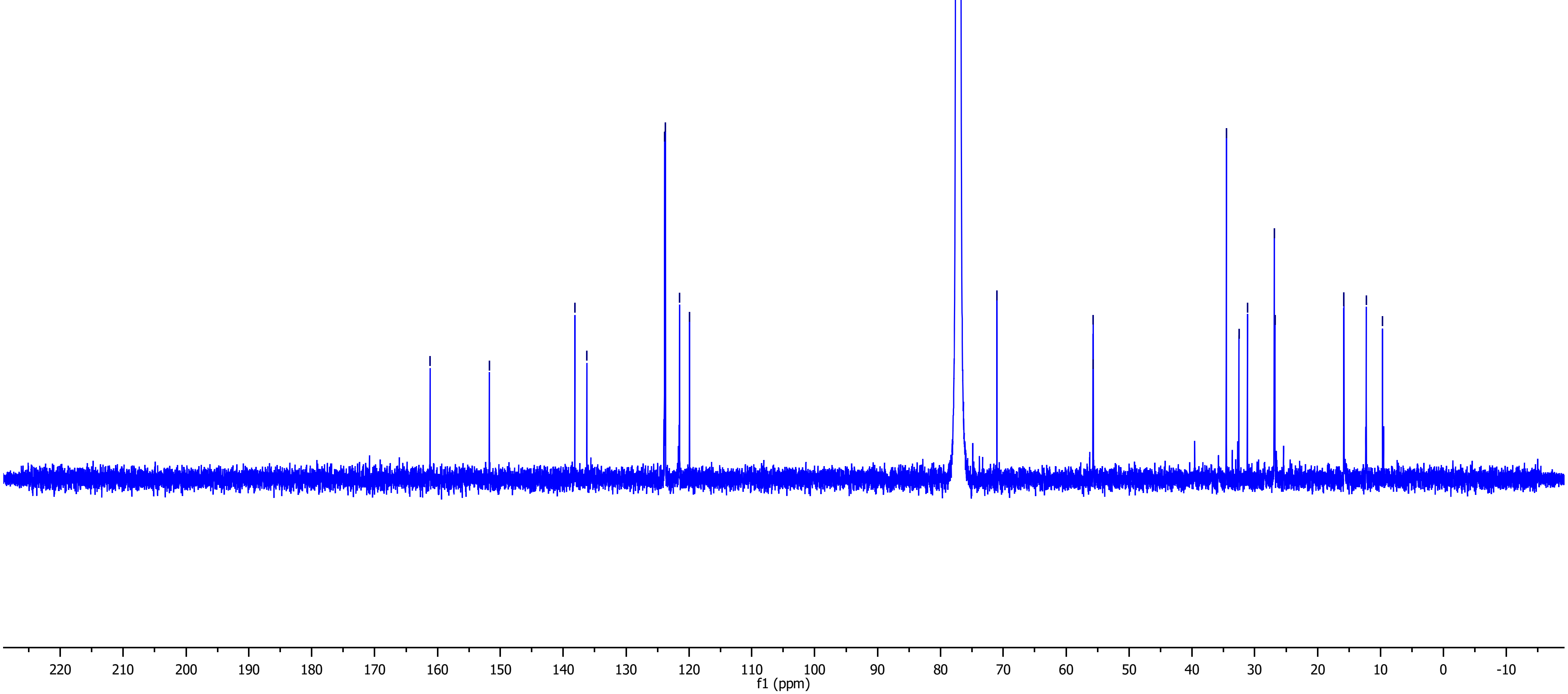

Parameter	Value
Instrument	Avance
Solvent	CDCl3
Experiment	1D
Spectrometer Frequency	100.67
Nucleus	13C

JNH_04_161 13C.1.fid — giese carbonylated Co(III)TAP TEMPO trap major UV active spot (35.2 mg)

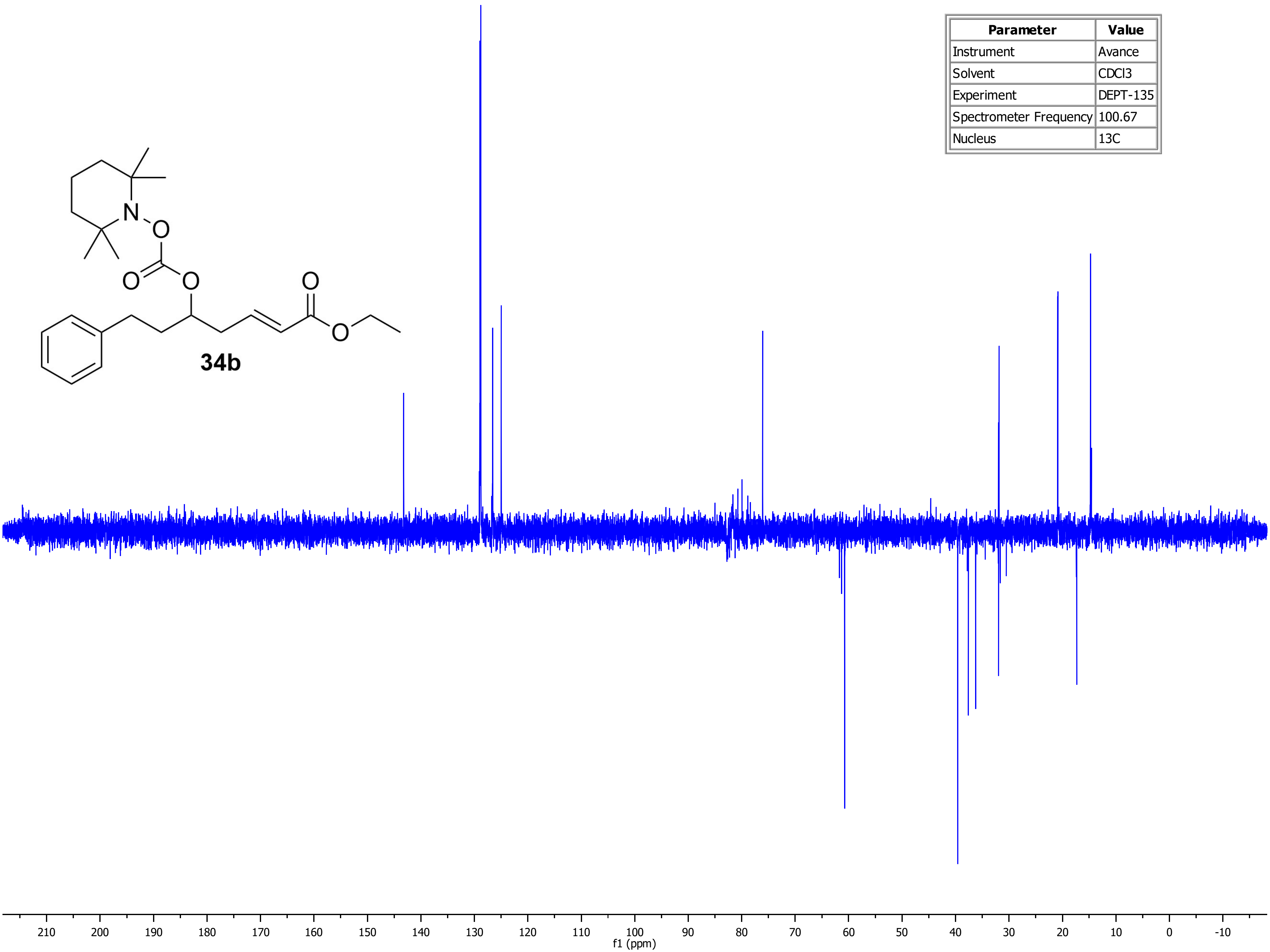

Parameter	Value
Instrument	Avance
Solvent	CDCl3
Experiment	DEPT-135
Spectrometer Frequency	100.67
Nucleus	13C

JNH_04_161 13C-DEPT135.1.fid — DEPT 135 giese carbonylated Co(III)TAP TEMPO trap major UV active spot (35.2 mg)

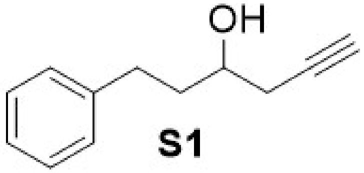

S1

Parameter	Value
Instrument	Avance NEO 500
Solvent	CDCl3
Experiment	1D
Spectrometer Frequency	500.30
Nucleus	1H

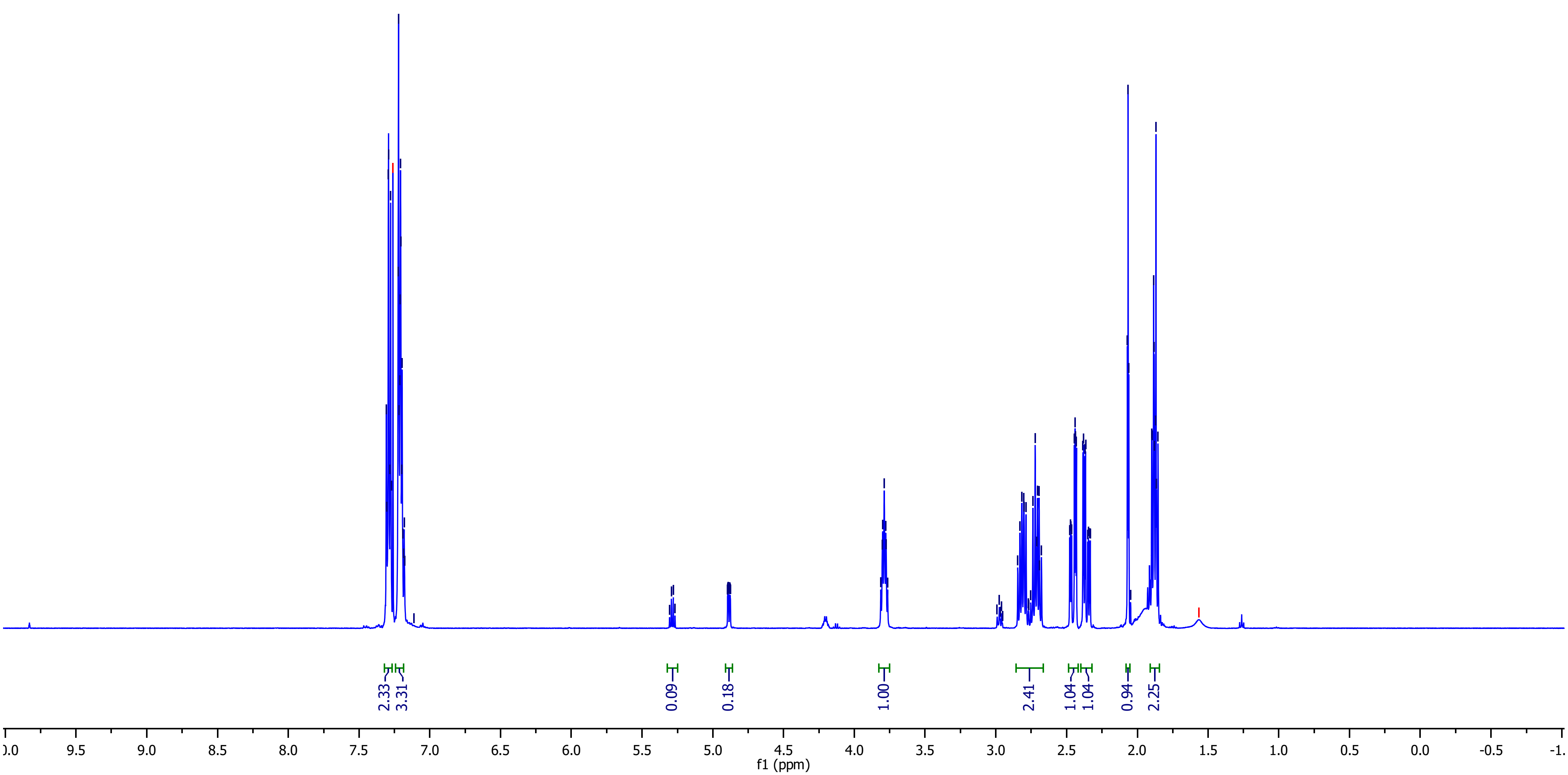

JNH_04_60.2.fid — dihydrocinnamaldehyde barbier propargylation after Yamadzen Column (1.4787g semipure)

Parameter	Value
Instrument	Avance NEO 500
Solvent	CDCl3
Experiment	1D
Spectrometer Frequency	500.30
Nucleus	1H

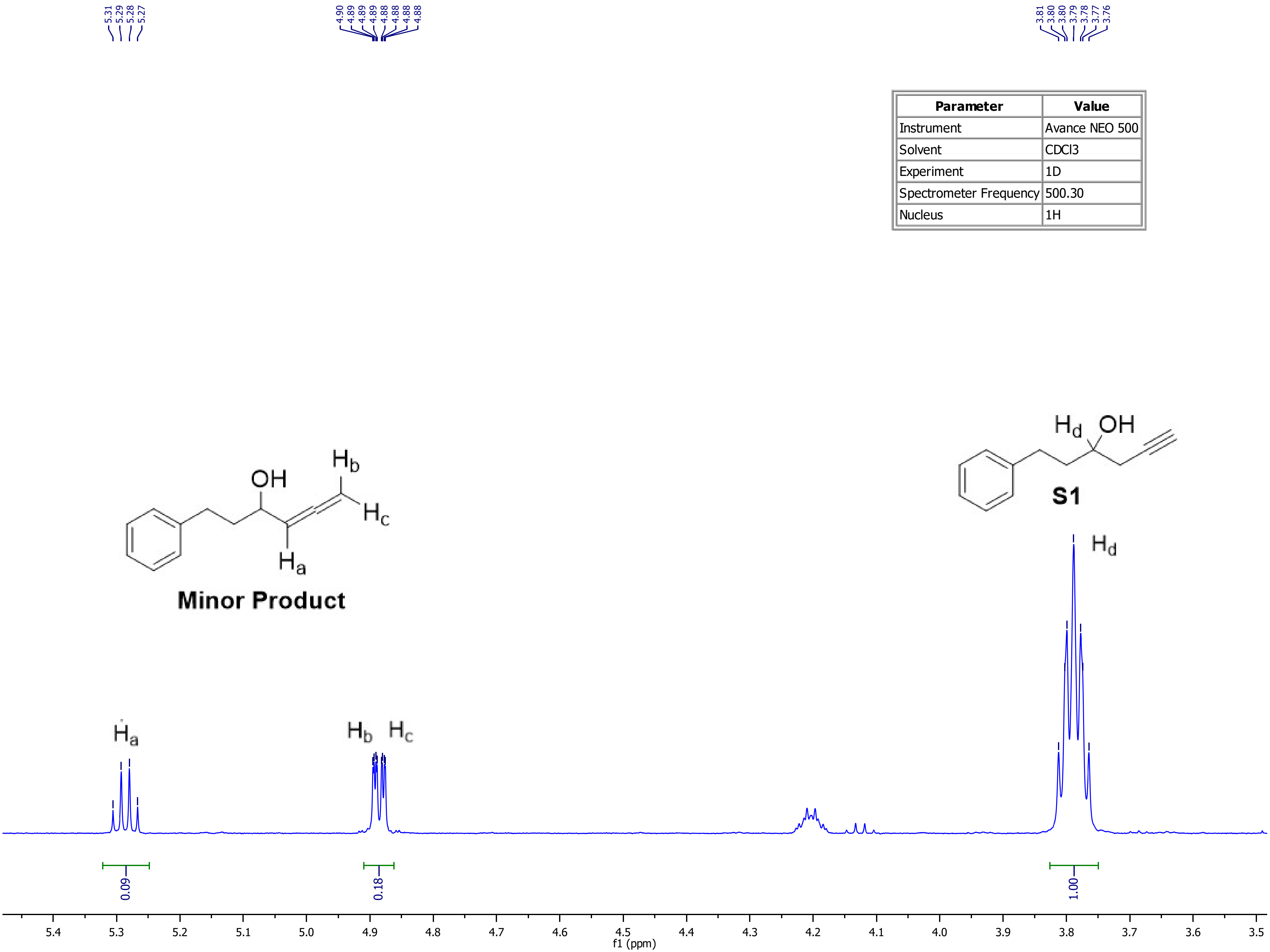

JNH_04_60.2.fid — dihydrocinnamaldehyde barbier propargylation after Yamadzen Column (1.4787g semipure)

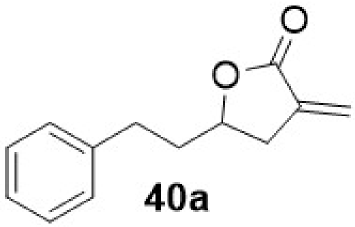

40a

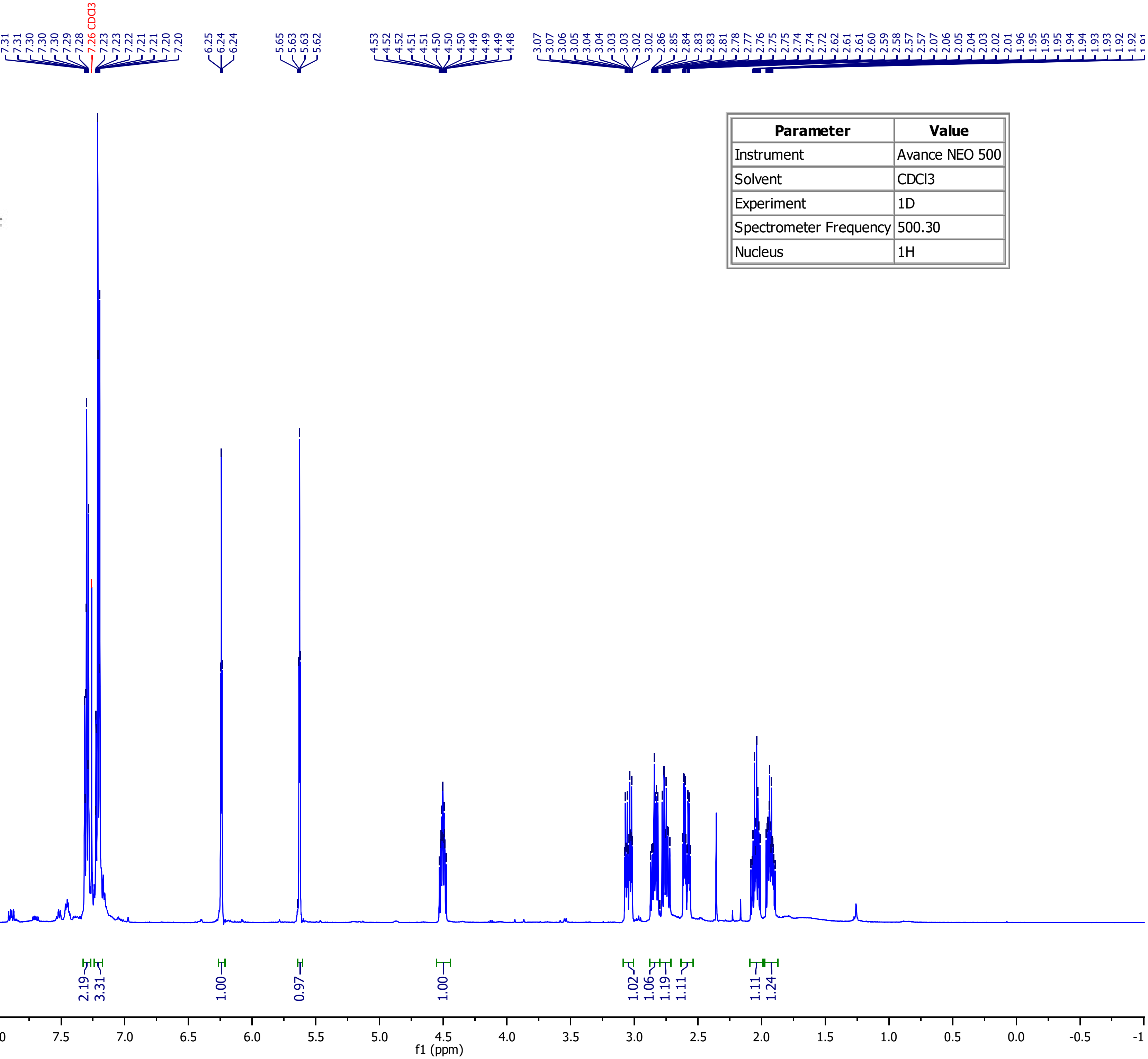

Parameter	Value
Instrument	Avance NEO 500
Solvent	CDCl3
Experiment	1D
Spectrometer Frequency	500.30
Nucleus	1H

JNH_04_91.1.fid — Pd(OAc)2 carbonylation

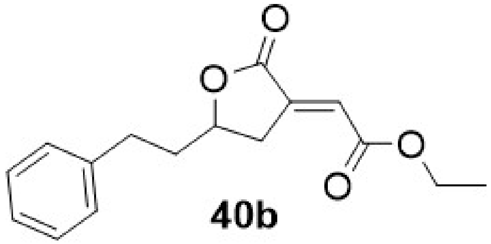

40b

Parameter	Value
Instrument	spect
Solvent	CDCl3
Experiment	1D
Spectrometer Frequency	600.21
Nucleus	1H

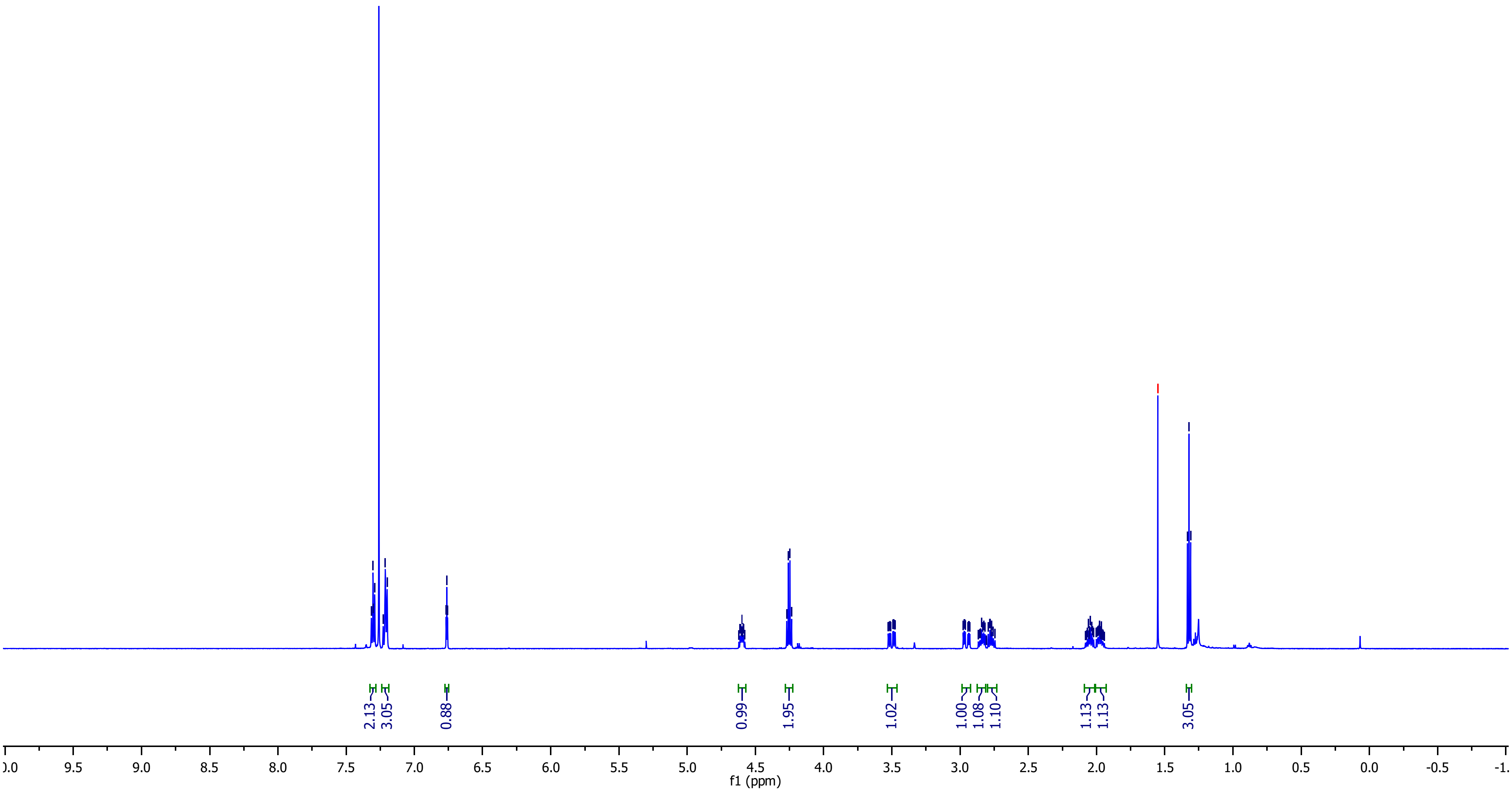

JNH_04_97.5.fid — Benzimidazole + Giese acceptor tuna sun 2nd top spot prep TLC

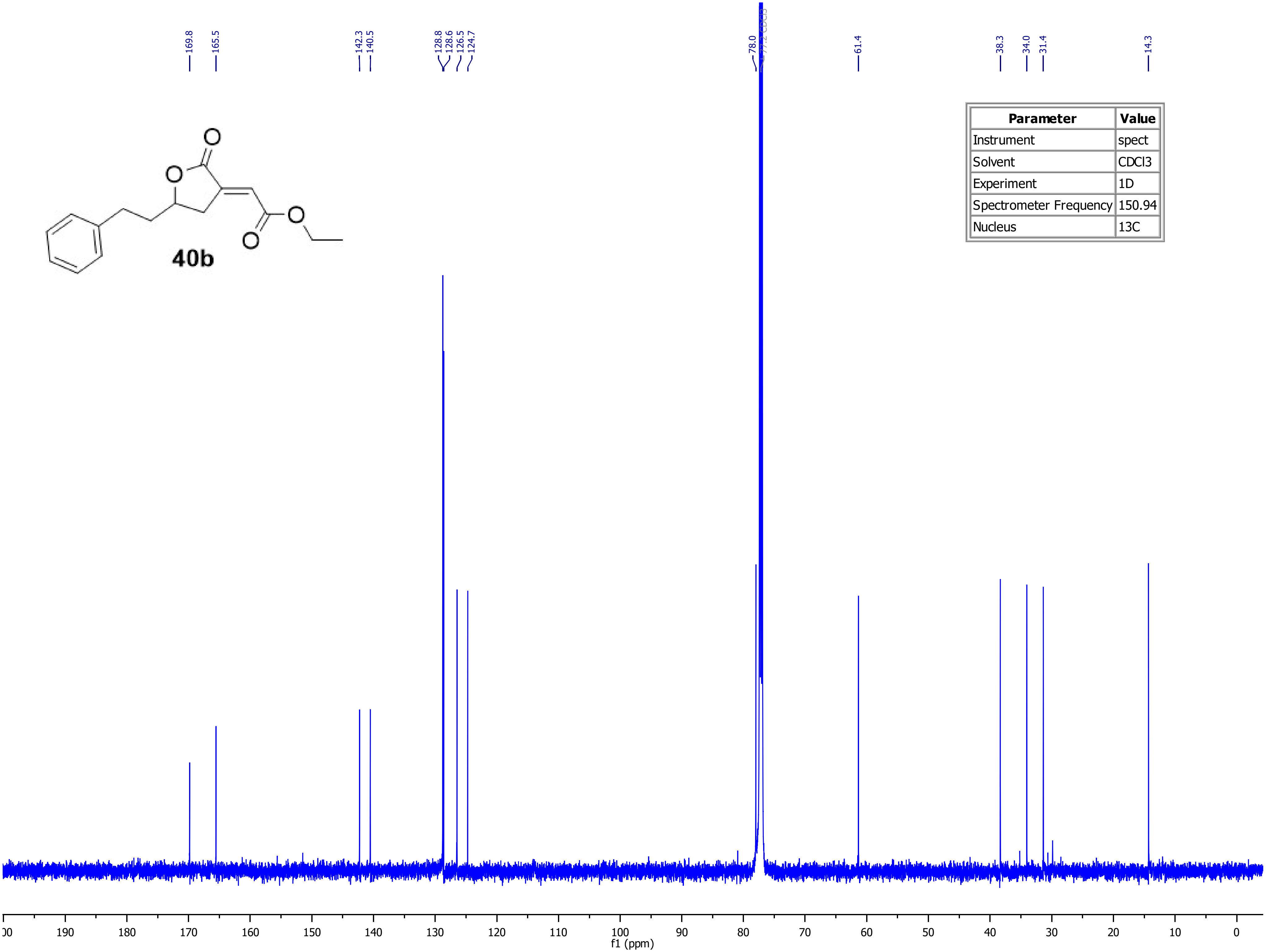

Parameter	Value
Instrument	spect
Solvent	CDCl3
Experiment	1D
Spectrometer Frequency	150.94
Nucleus	13C

JNH_04_97 13C.5.fid — Endocyclic giese product

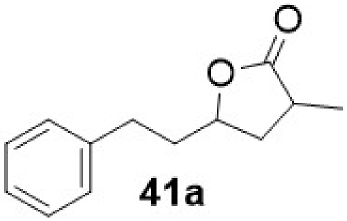

41a

Parameter	Value
Instrument	Avance
Solvent	CDCl3
Experiment	1D
Spectrometer Frequency	400.30
Nucleus	1H

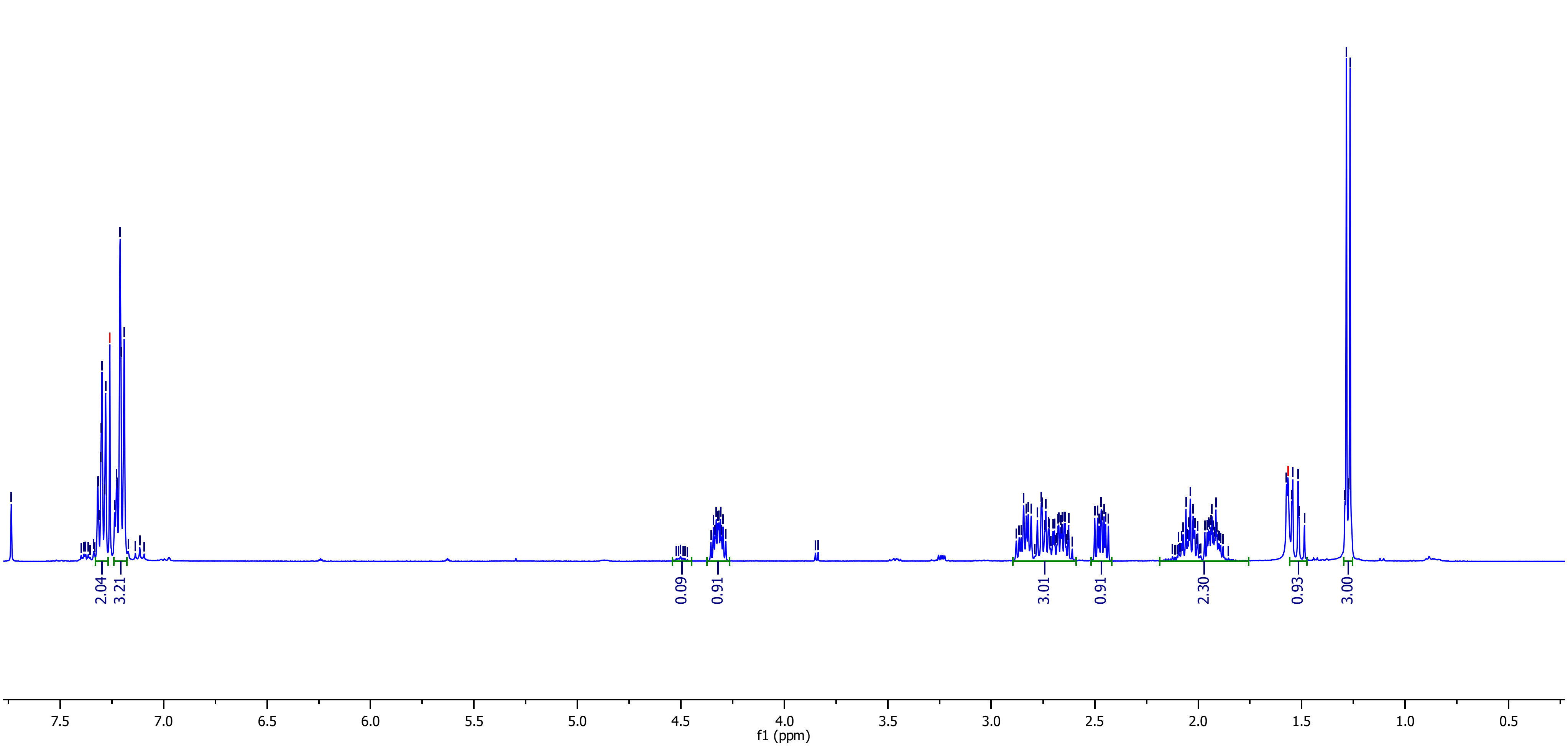

TKB_01_PdC purification.2.fid — Pd/C autocolumn late fraction "yellow top"

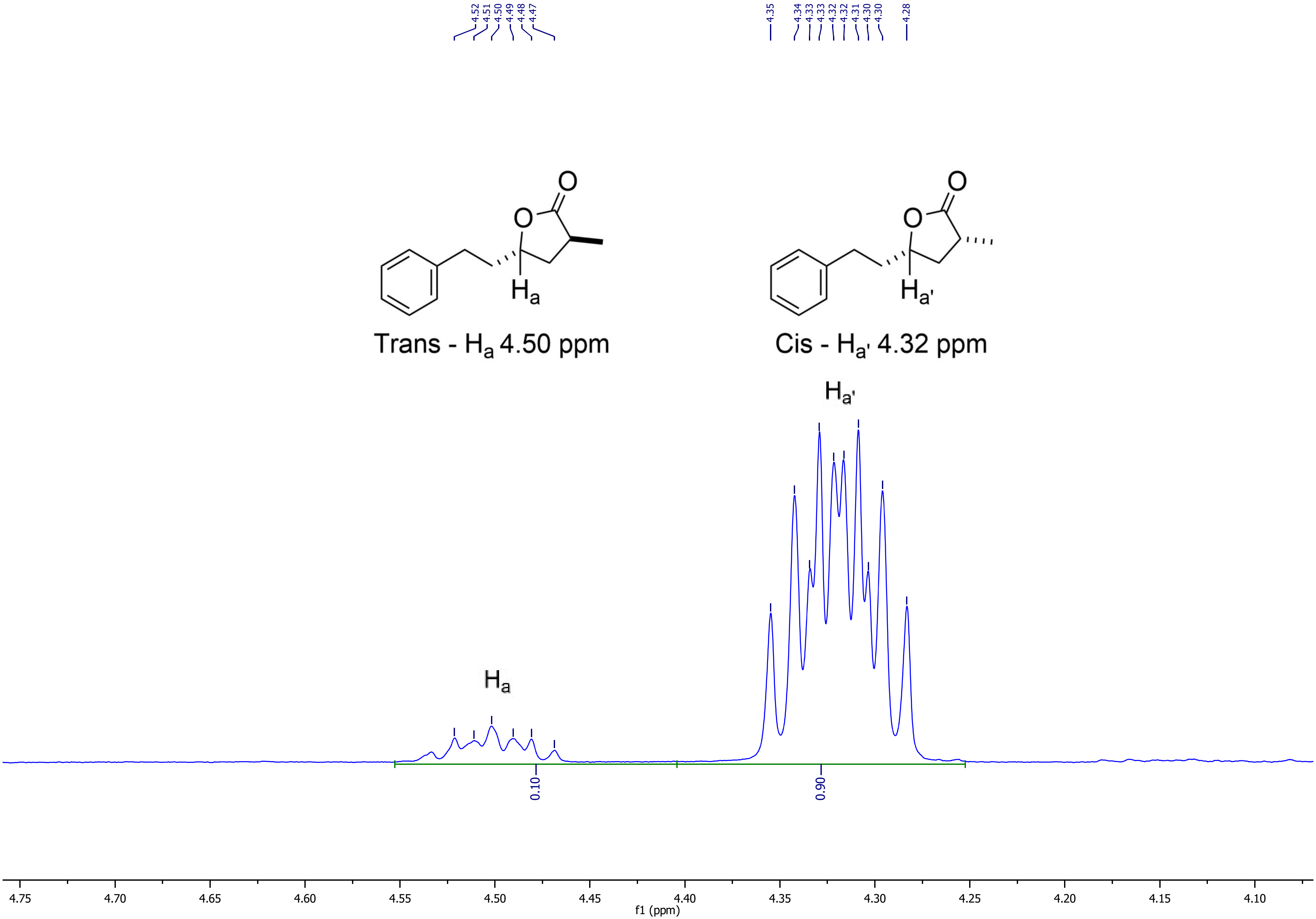

TKB_01_PdC purification.2.fid — Pd/C autocolumn late fraction "yellow top"

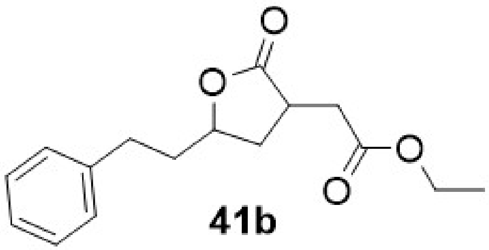

41b

Parameter	Value
Instrument	Avance NEO 500
Solvent	CDCl3
Experiment	1D
Spectrometer Frequency	500.30
Nucleus	1H

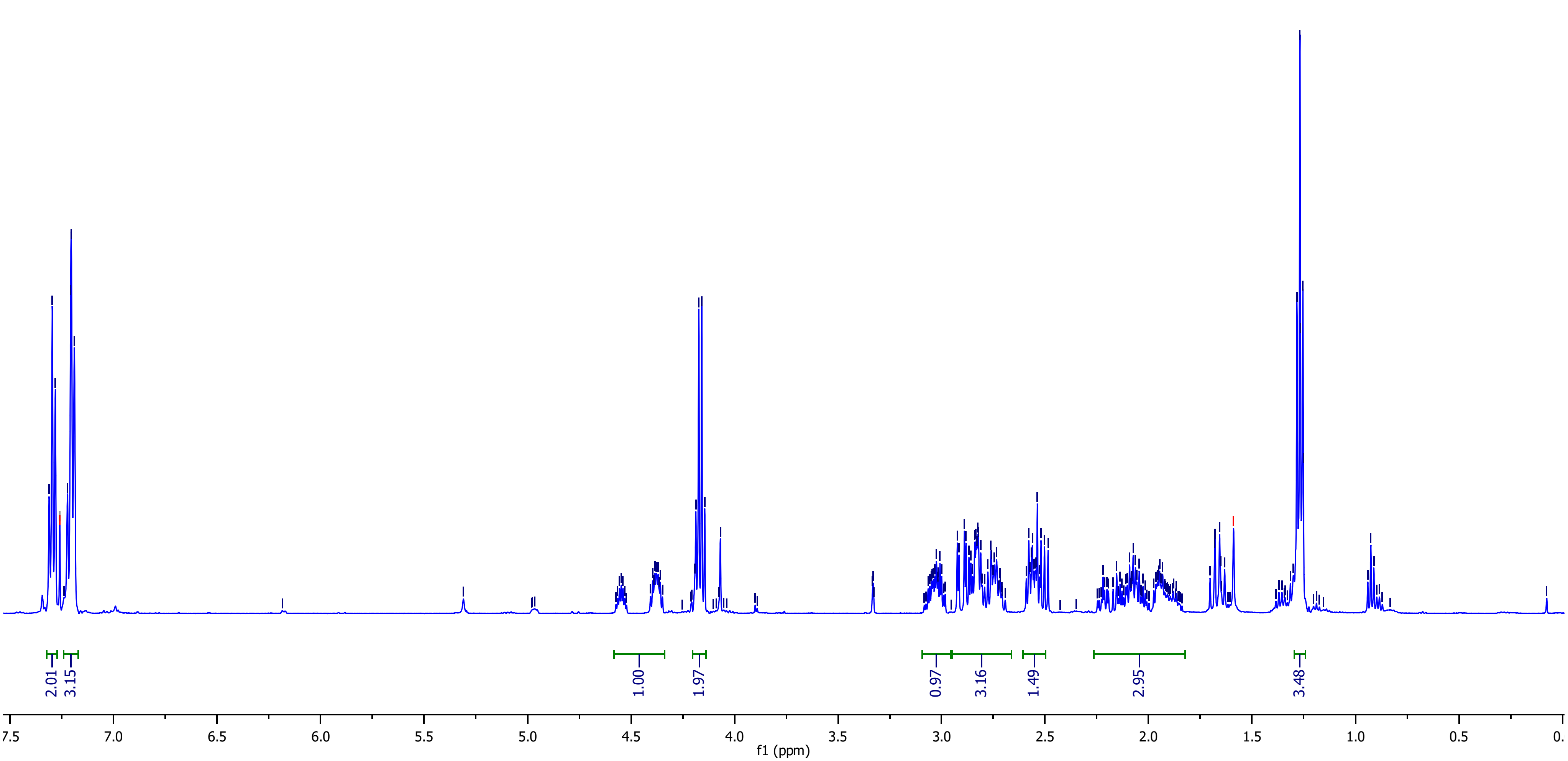

JNH_04_212.8.fid — tributyl tin hydride reduction of giese lactone frac 20-23

Parameter	Value
Instrument	Avance NEO 500
Solvent	CDCl3
Experiment	1D
Spectrometer Frequency	500.30
Nucleus	1H

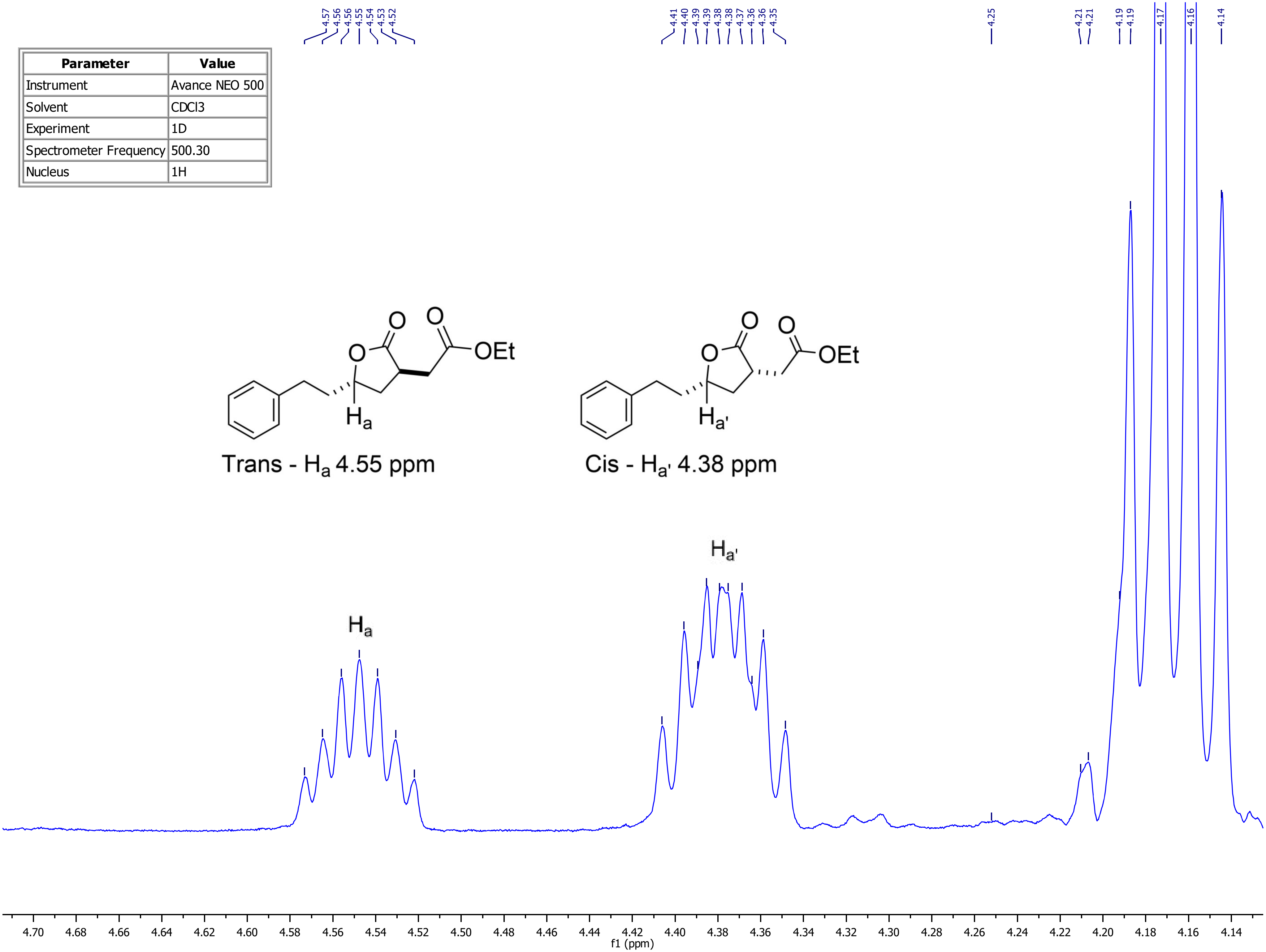

JNH_04_212.8.fid — tributyl tin hydride reduction of giese lactone frac 20-23

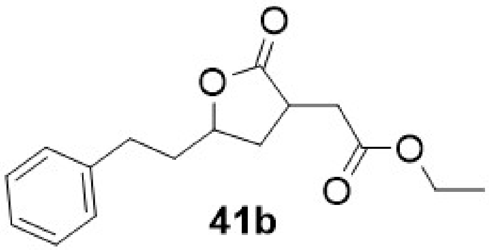

41b

Parameter	Value
Instrument	Avance NEO 500
Solvent	CDCl3
Experiment	1D
Spectrometer Frequency	125.82
Nucleus	13C

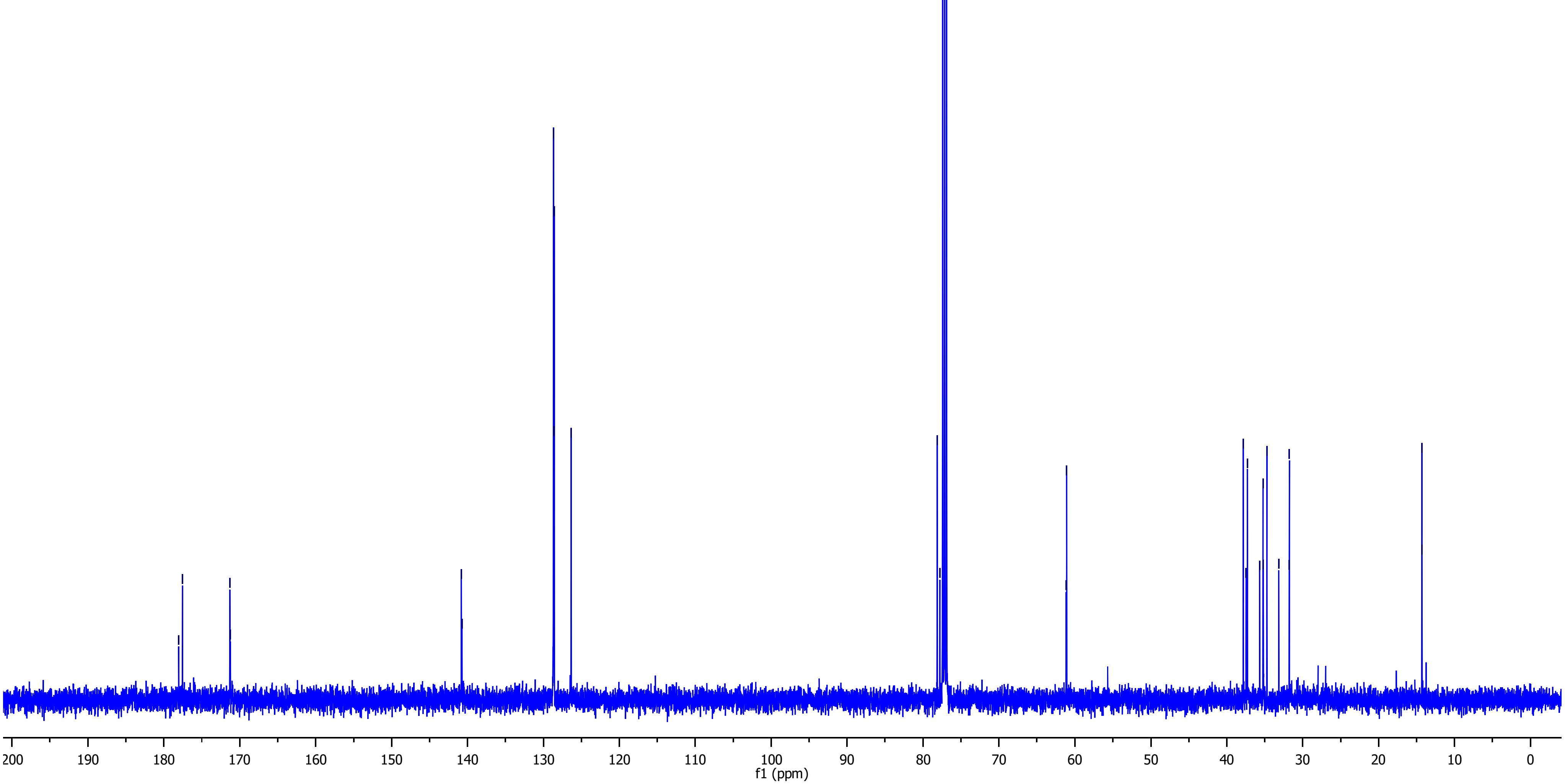

JNH_04_212 13C.8.fid — tributyl tin hydride reduction of giese lactone frac 20-23

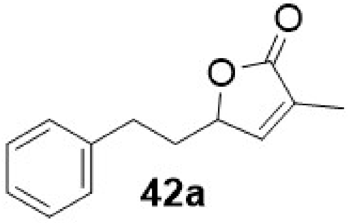

42a

Parameter	Value
Instrument	Avance
Solvent	CDCl3
Experiment	1D
Spectrometer Frequency	400.30
Nucleus	1H

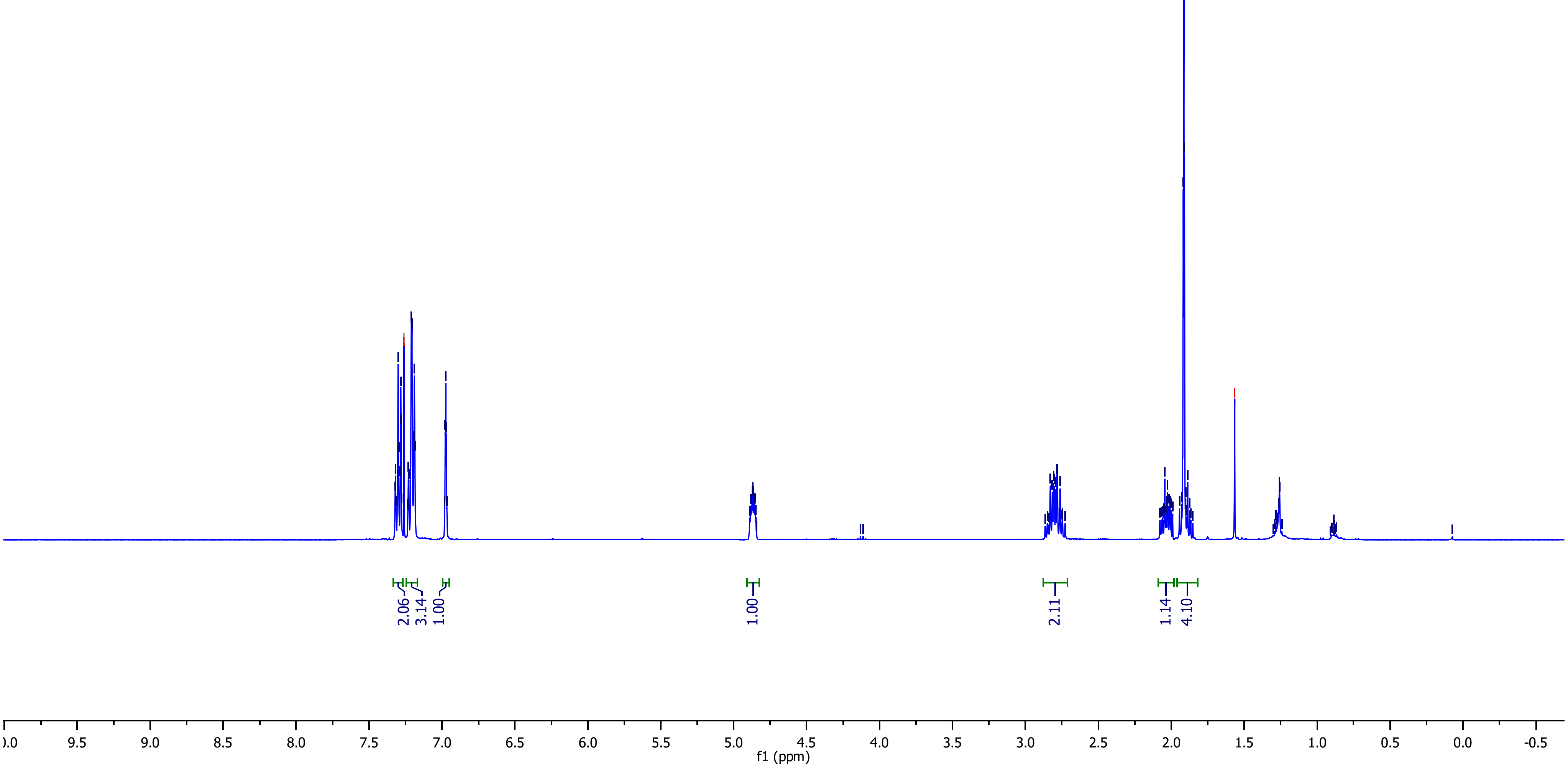

TKB_01_18_pure.3.fid — Last fraction from autocolumn 7/11/24

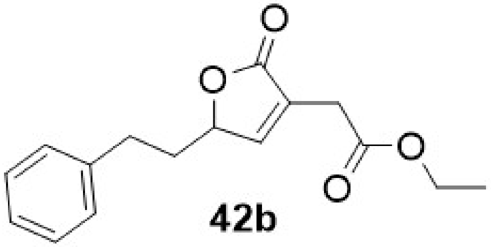

42b

Parameter	Value
Instrument	spect
Solvent	CDCl3
Experiment	1D
Spectrometer Frequency	600.21
Nucleus	1H

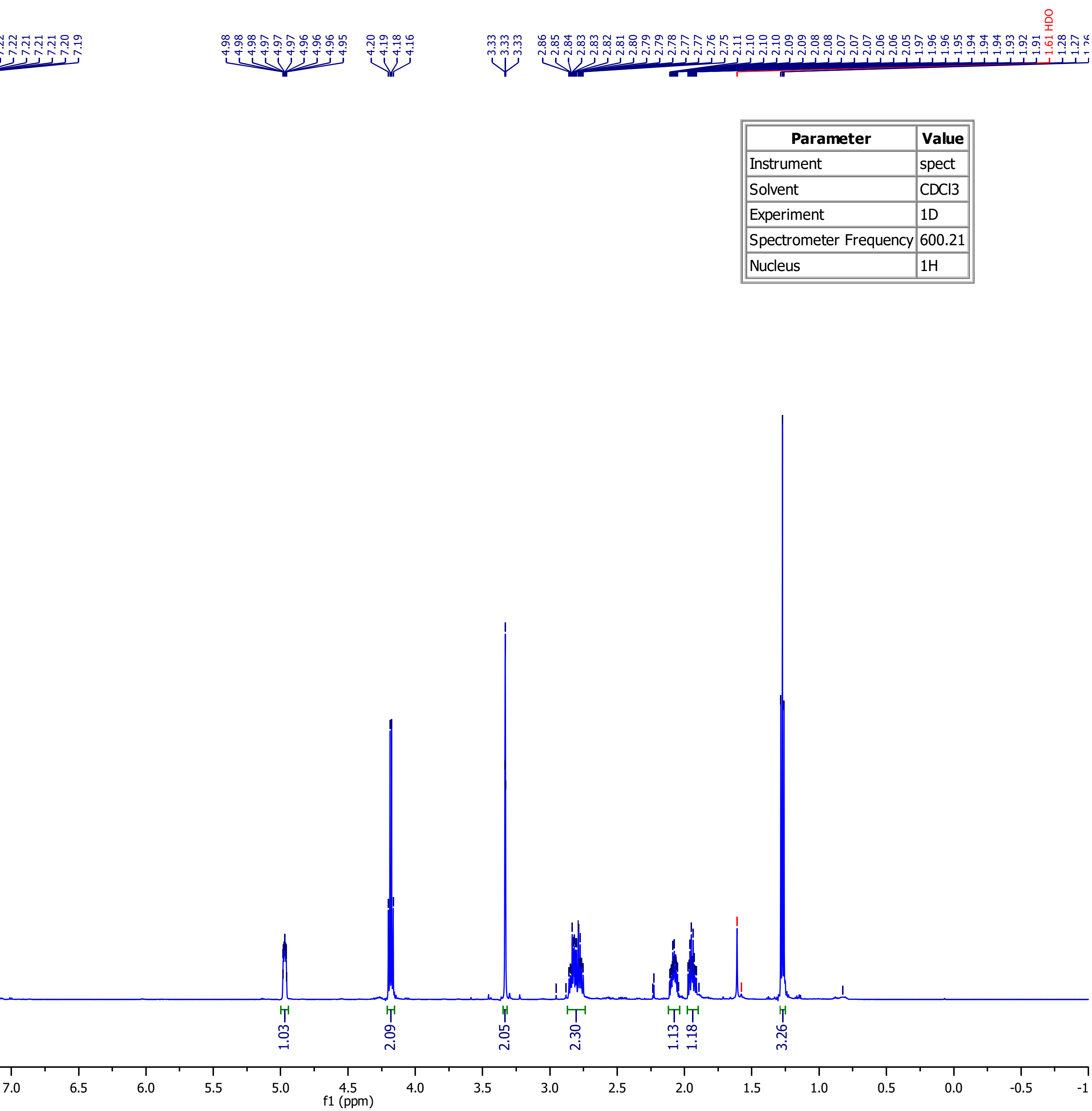

JNH_04_85.1.fid — rxn 1.) 10mg conserved product across all 3 reactions reacts with KMnO4

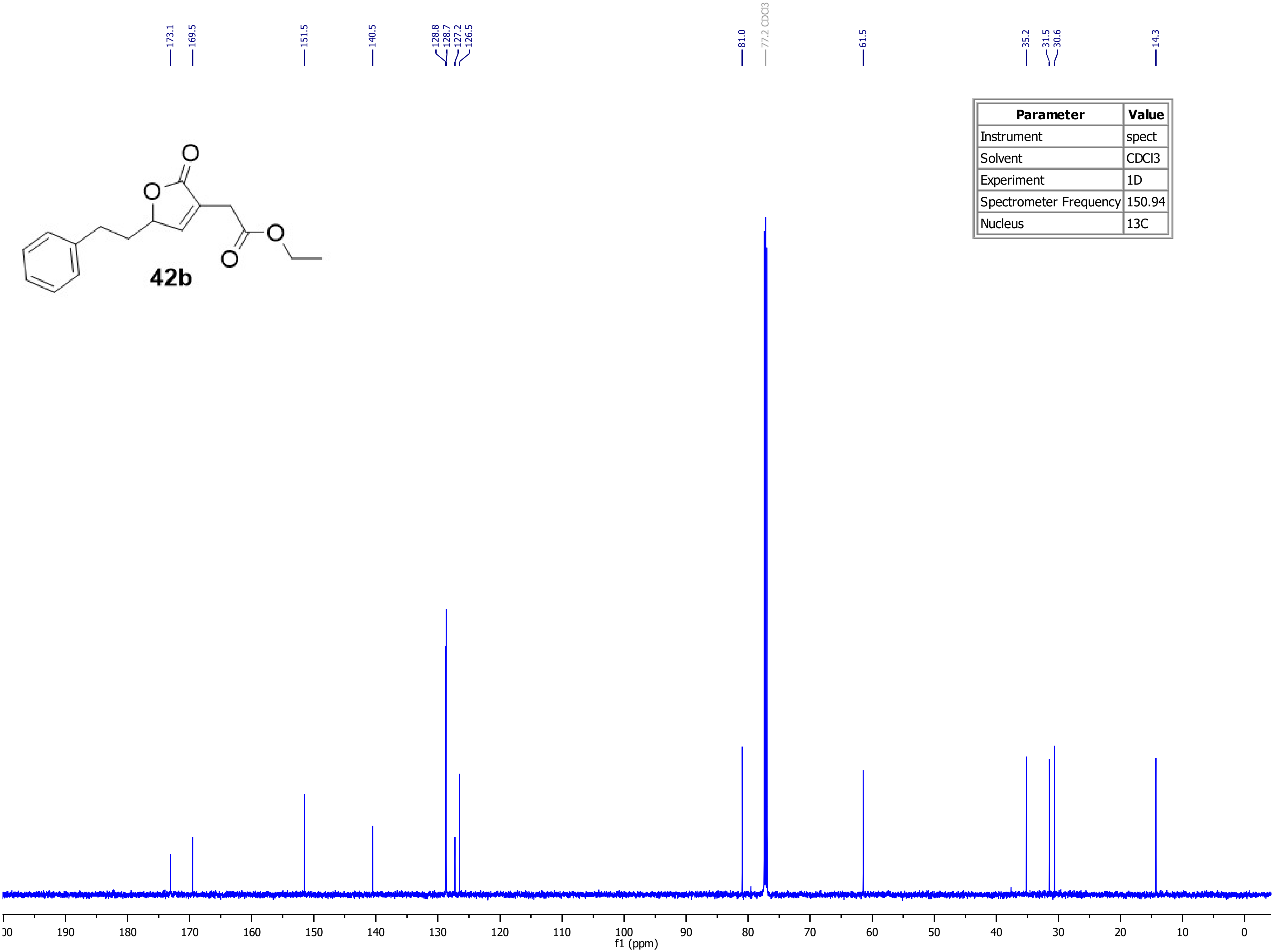

Parameter	Value
Instrument	spect
Solvent	CDCl3
Experiment	1D
Spectrometer Frequency	150.94
Nucleus	13C

JNH_04_85 13C.1.fid — rxn 1.) 10mg conserved product across all 3 reactions reacts with KMnO4

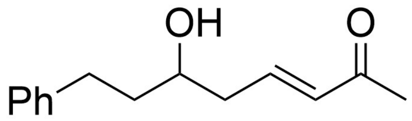

32c

Parameter	Value
Instrument	Avance NEO 500
Solvent	CDCl3
Experiment	1D
Spectrometer Frequency	500.30
Nucleus	1H

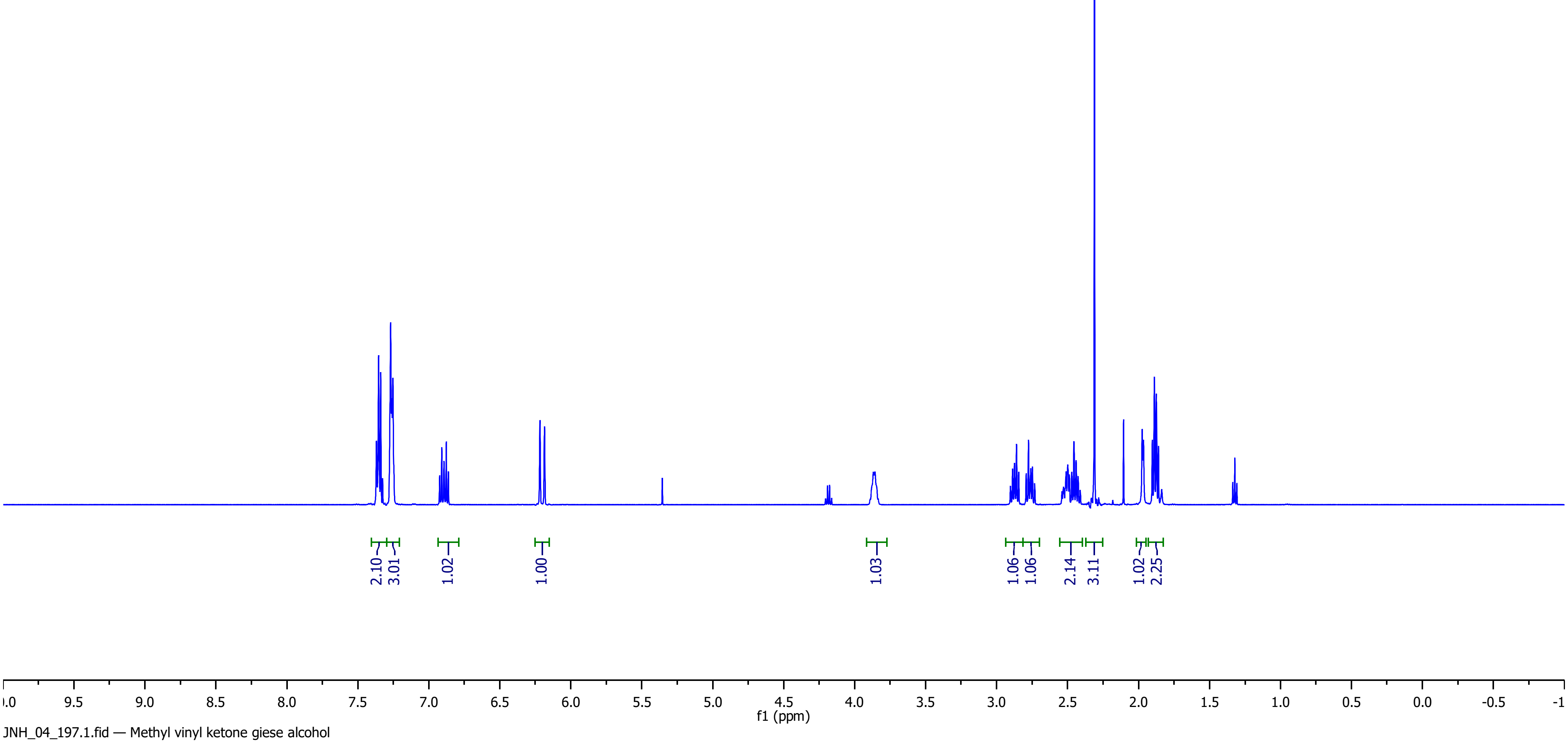

JNH_04_197.1.fid — Methyl vinyl ketone giese alcohol

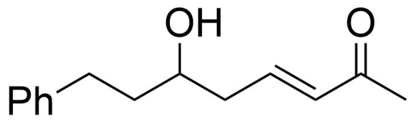

32c

Parameter	Value
Instrument	Avance NEO 500
Solvent	CDCl3
Experiment	1D
Spectrometer Frequency	125.82
Nucleus	13C

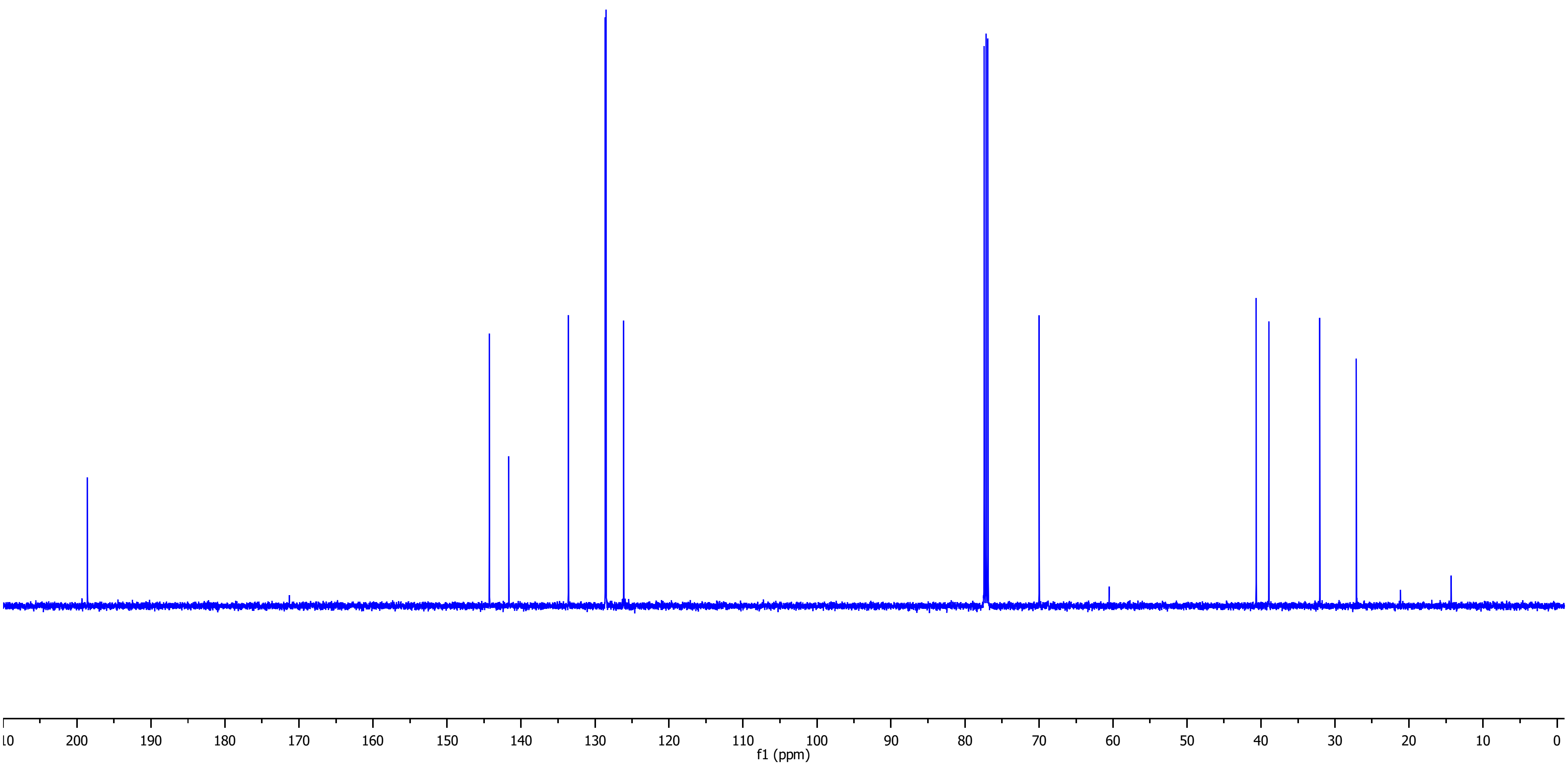

JNH_04_197 13C.1.fid — Methyl vinyl ketone giese alcohol

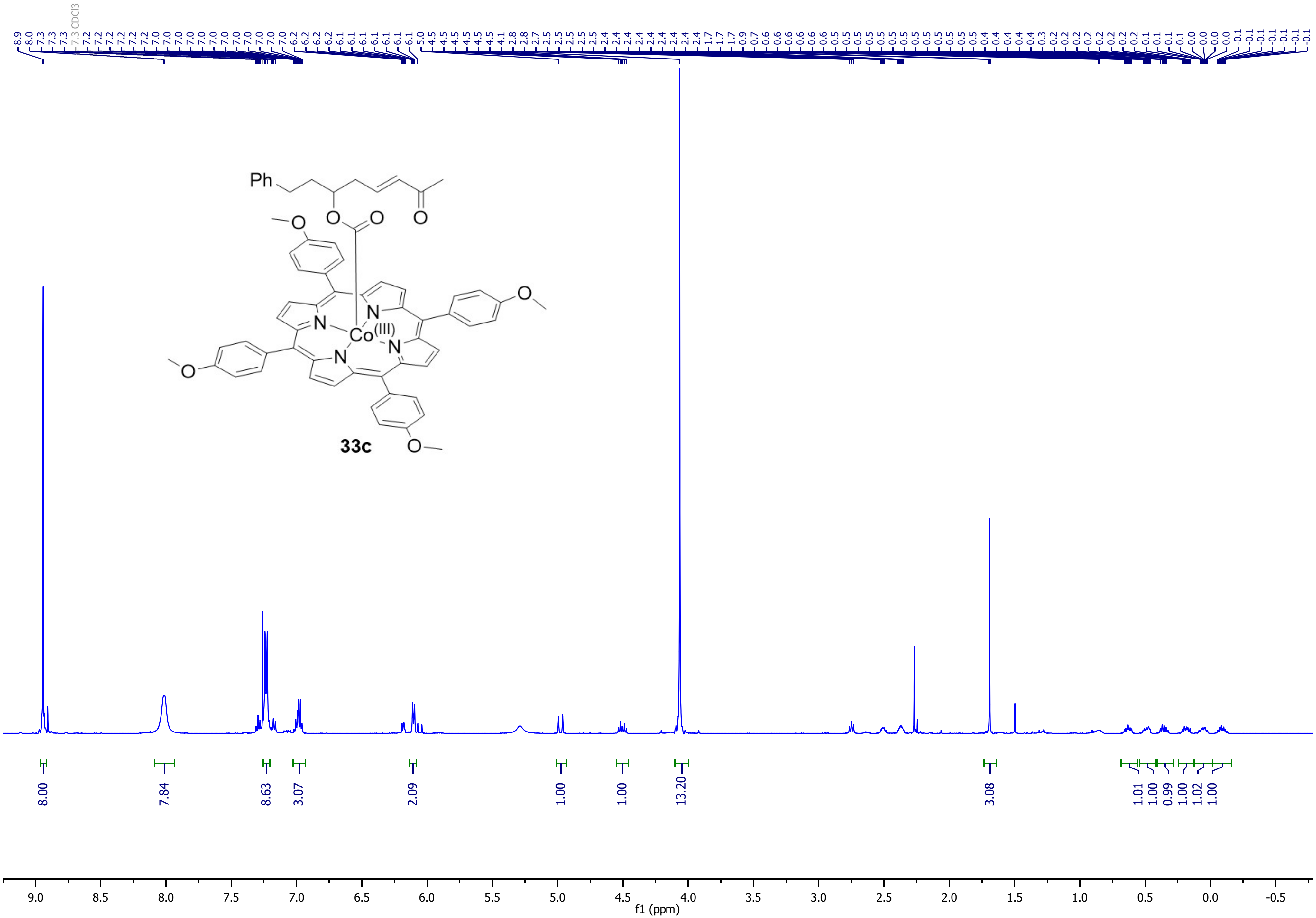

JNH_04_200.2.fid — carbonylated methyl vinyl ketone alcohol post 2nd purification

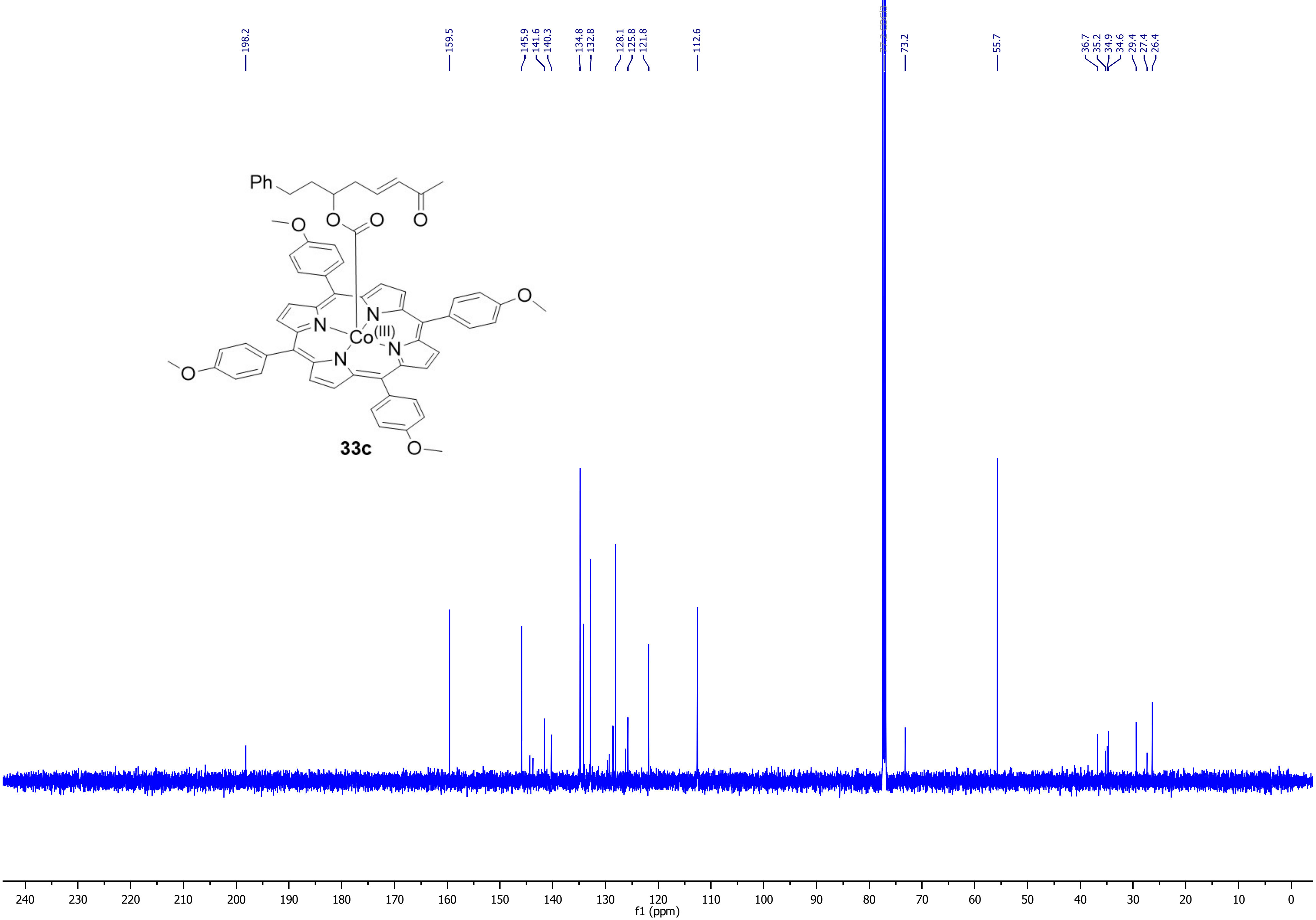

JNH_04_200 13C.2.fid — carbonylated methyl vinyl ketone alcohol. post second purification

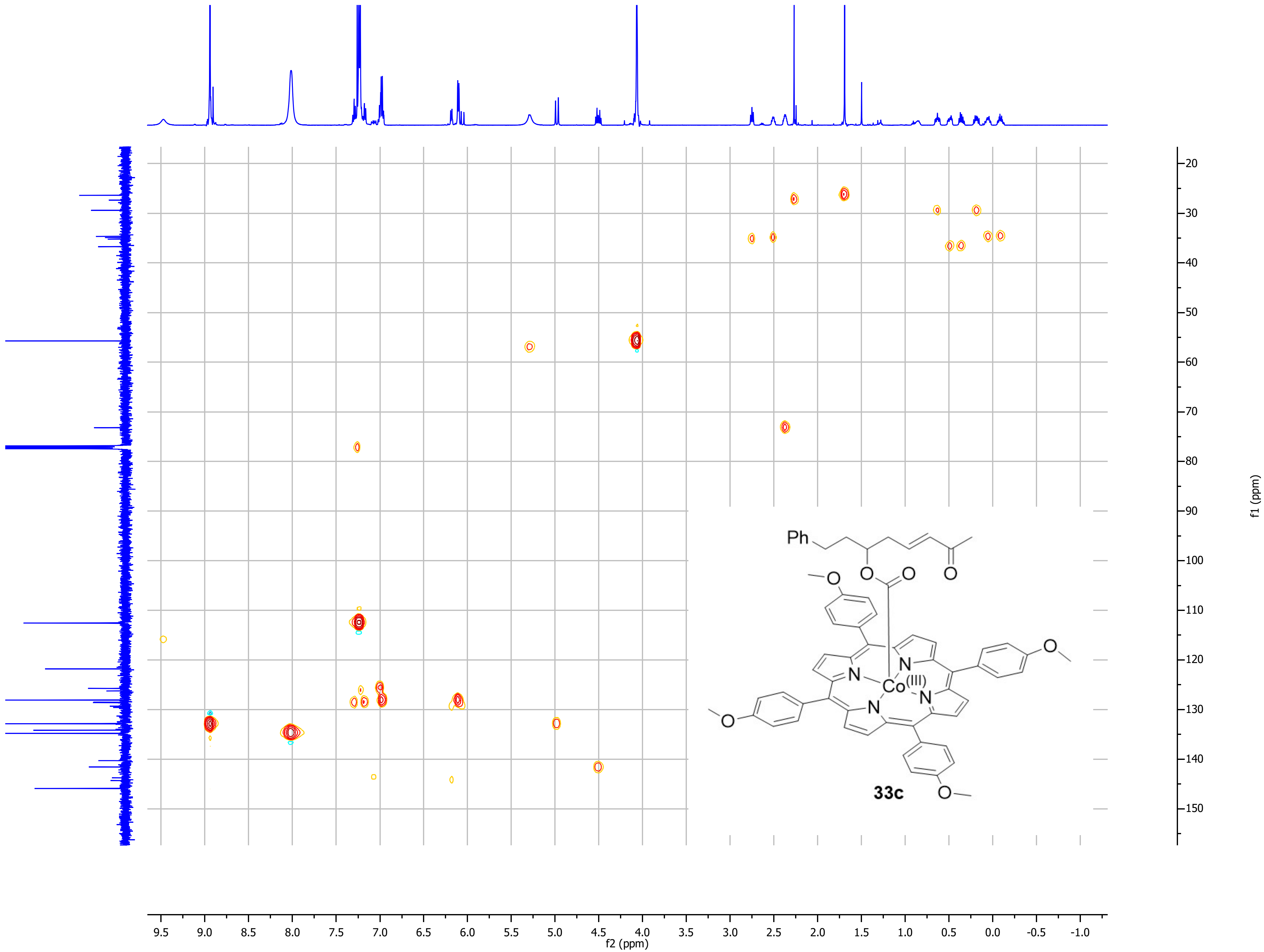

JNH_04_200 C13_FASTHSQC.2.ser — MVK carbonylation product

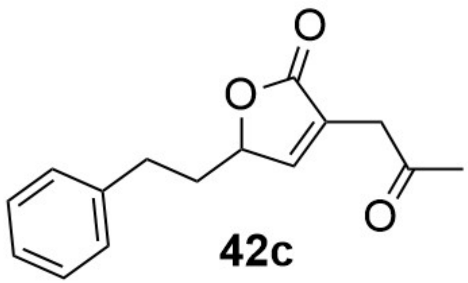

42c

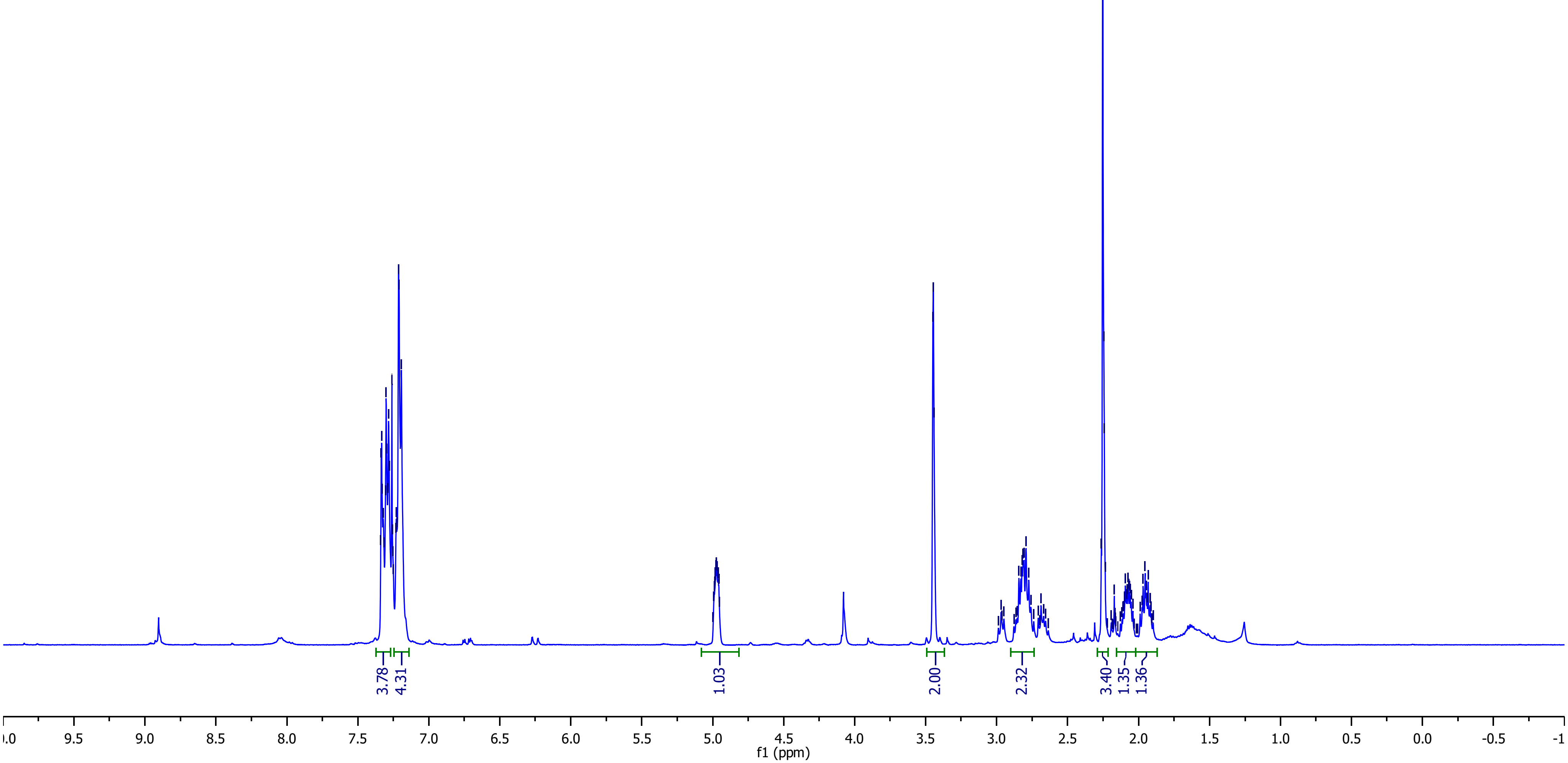

Parameter	Value
Instrument	Avance
Solvent	CDCl3
Experiment	1D
Spectrometer Frequency	400.30
Nucleus	1H

JNH_04_201.6.fid — methyl vinyl ketone tuna sun frac 6 acetone hexane 1:3 column 2 . Rf 0.3

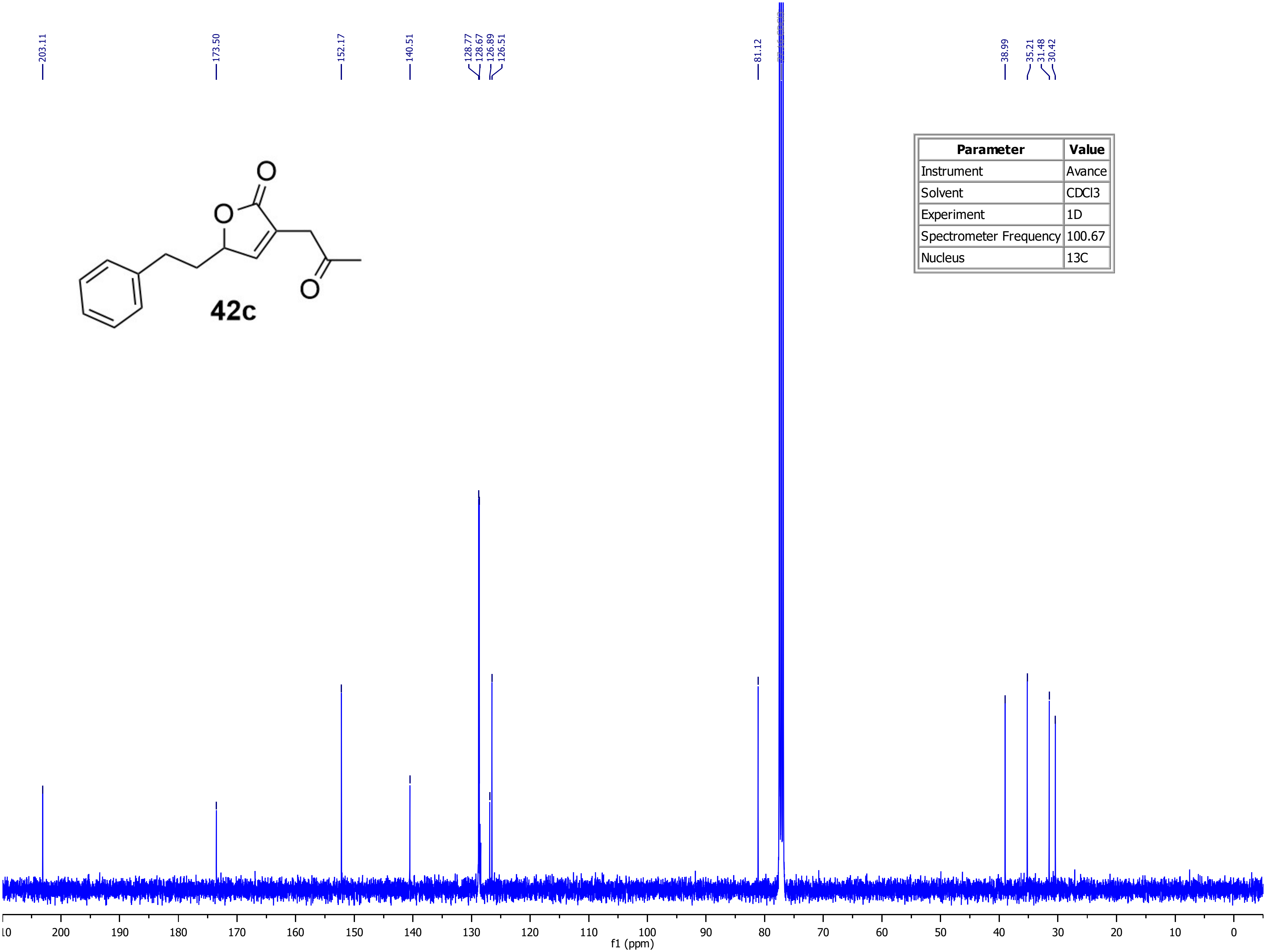

Parameter	Value
Instrument	Avance
Solvent	CDCl3
Experiment	1D
Spectrometer Frequency	100.67
Nucleus	13C

JNH_04_201 13C.6.fid — methyl vinyl ketone tuna sun frac 6 acetone hexane 1:3 column 2 . Rf 0.3

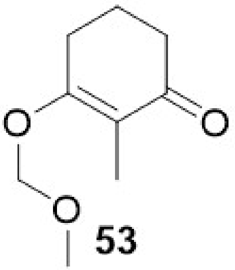

53

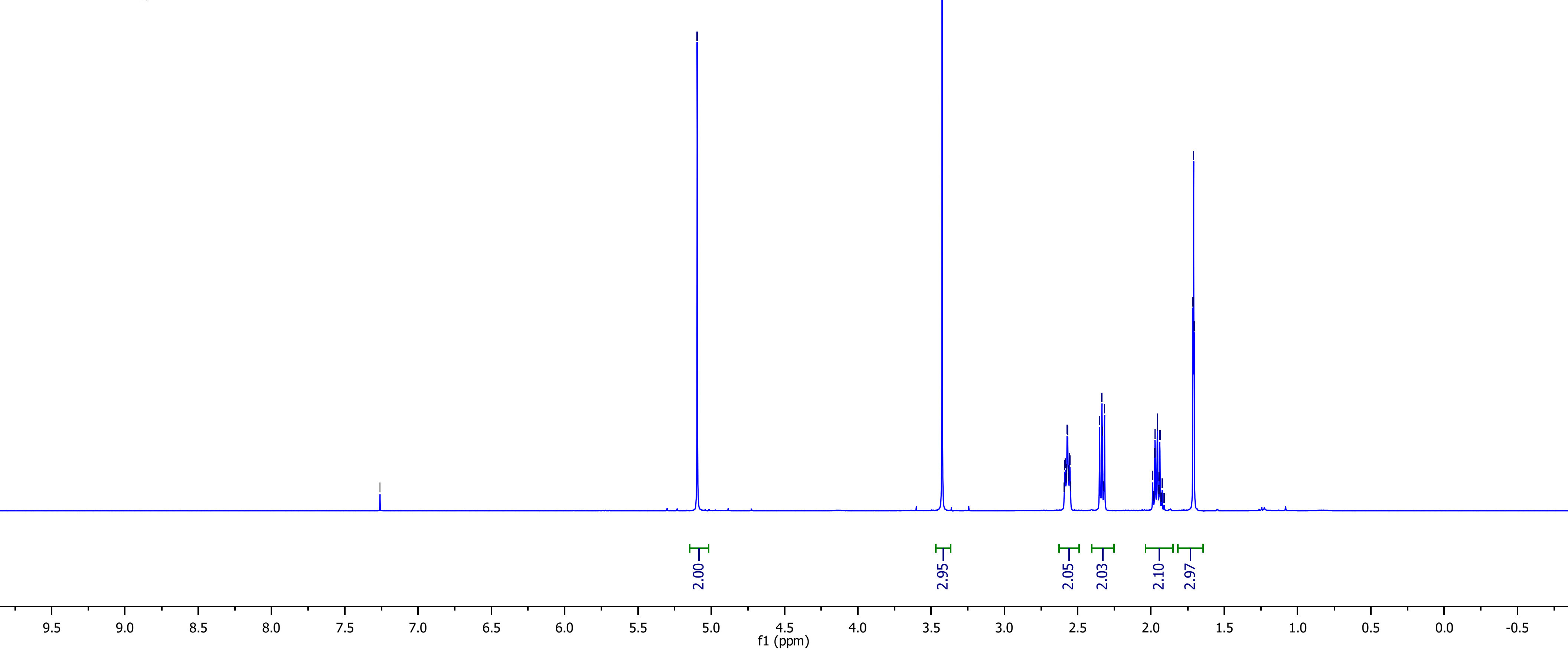

Parameter	Value
Instrument	Avance
Solvent	CDCl3
Experiment	1D
Spectrometer Frequency	400.30
Nucleus	1H

_04_72.1.fid — MOM Protected methyl diketone (vinylogous ester) after autocolumn (9.3457 g)

53

Parameter	Value
Instrument	Avance
Solvent	CDCl3
Experiment	1D
Spectrometer Frequency	100.67
Nucleus	13C

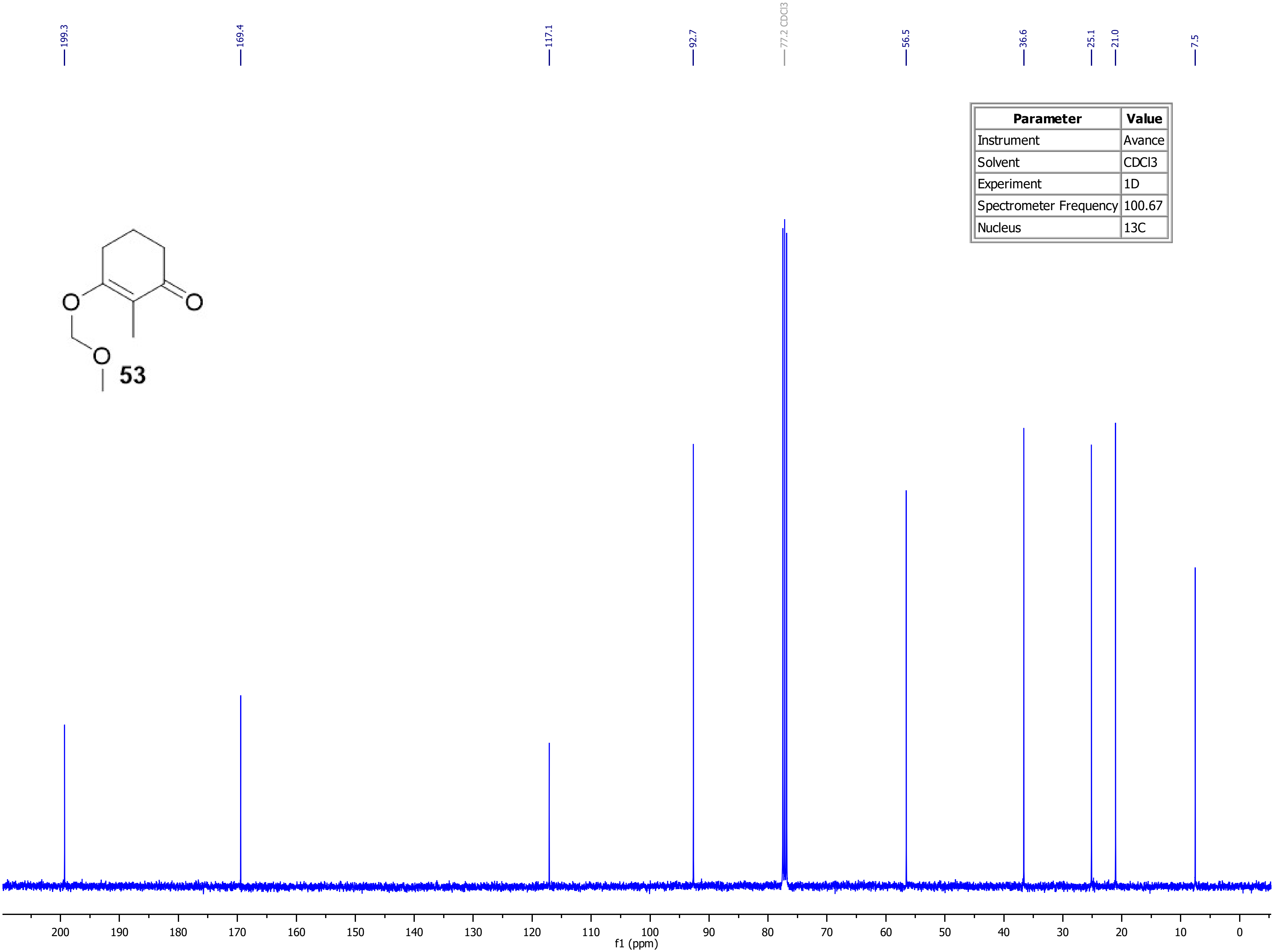

JNH_04_72 13C.1.fid — JNH_04_72 13C MOM protected methyl diketone (vinylogous ester) 9.3457 g

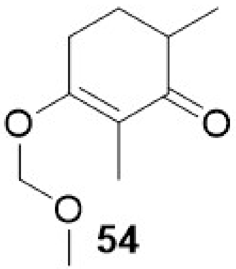

54

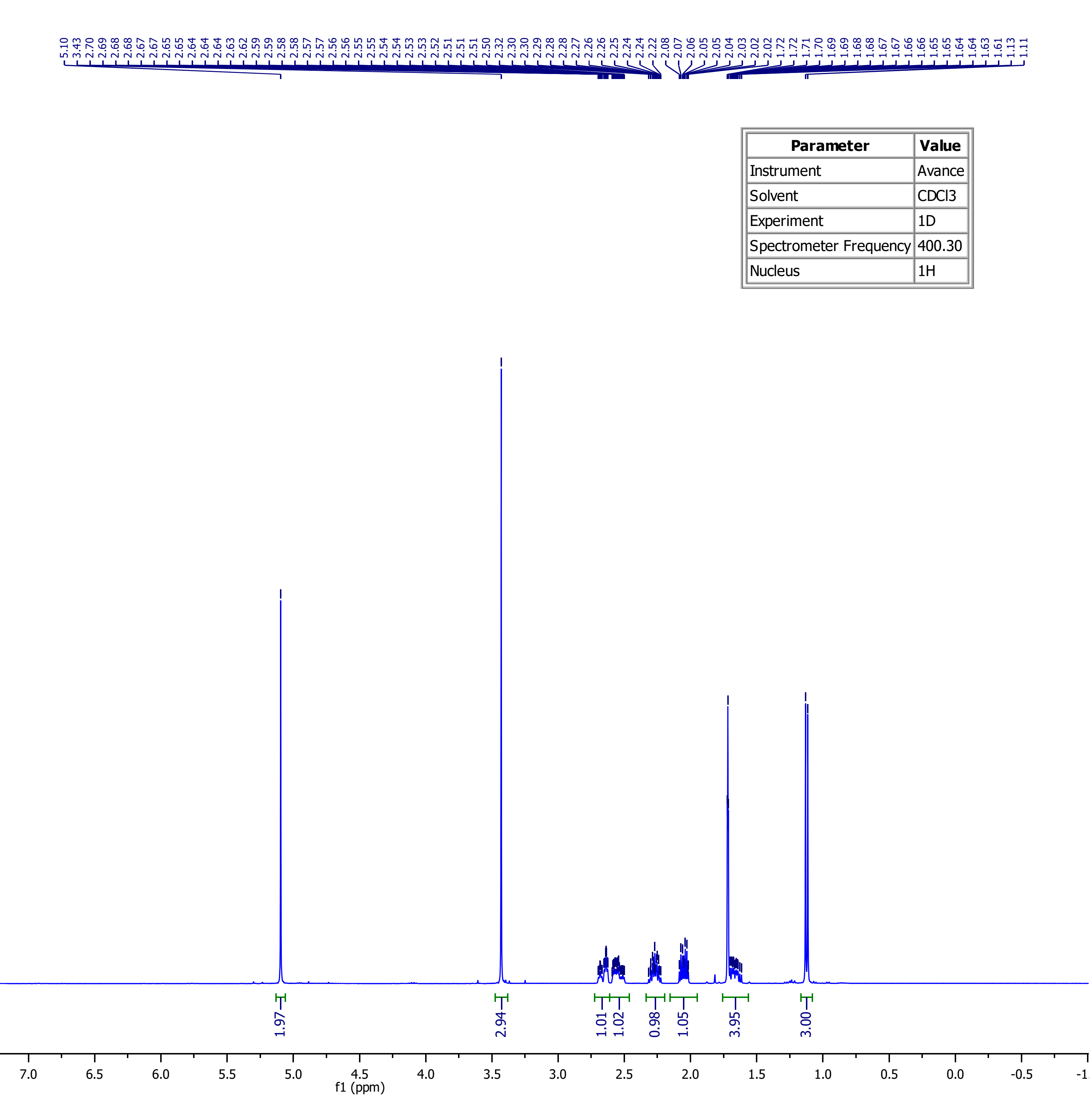

Parameter	Value
Instrument	Avance
Solvent	CDCl3
Experiment	1D
Spectrometer Frequency	400.30
Nucleus	1H

JNH_04_73.1.fid — Methyl Mom protected cyclic vinylogous ester

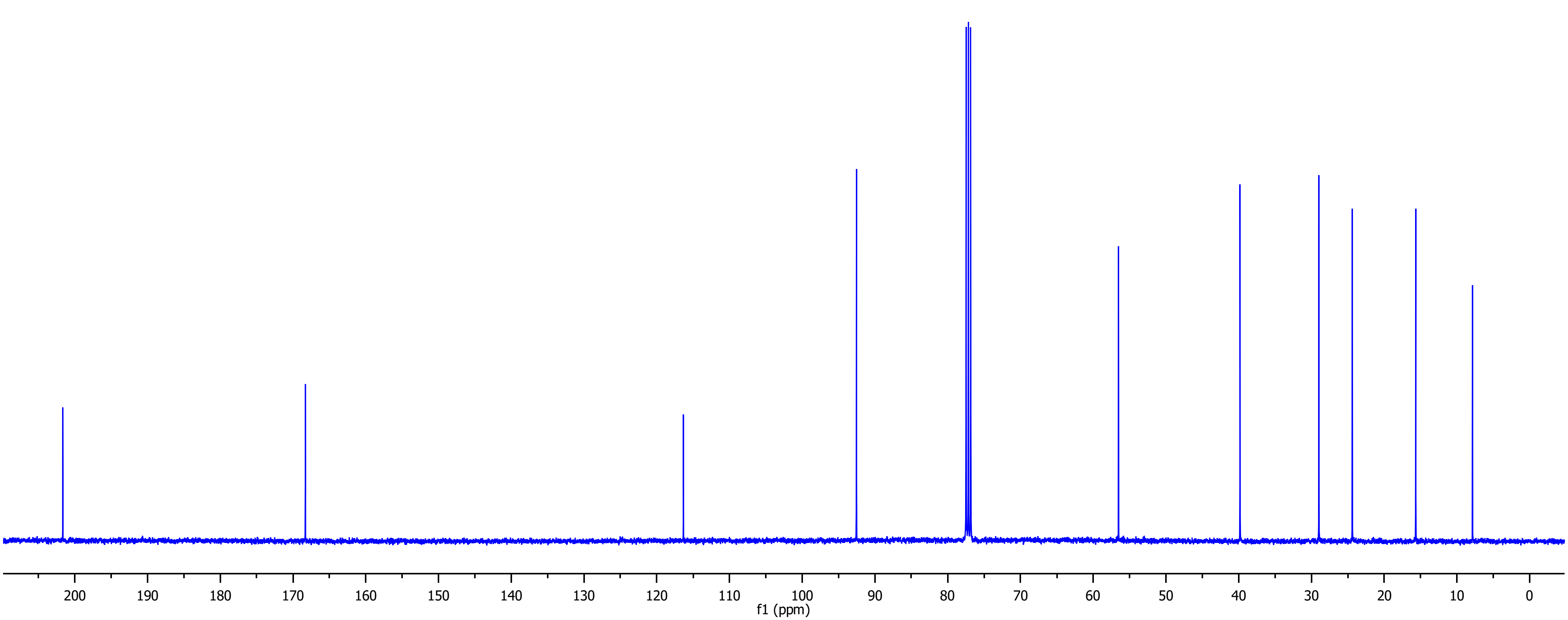

Parameter	Value
Instrument	Avance
Solvent	CDCl3
Experiment	1D
Spectrometer Frequency	100.67
Nucleus	13C

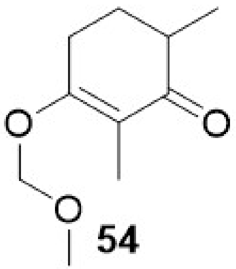

54

JNH_04_73 13C.1.fid — Methyl Mom protected cyclic vinylogous ester

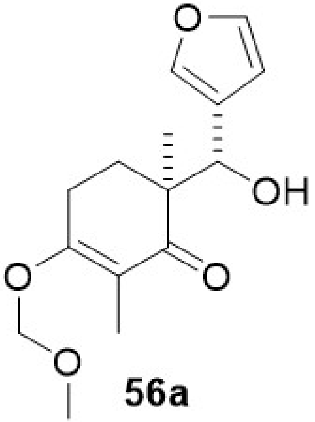

56a

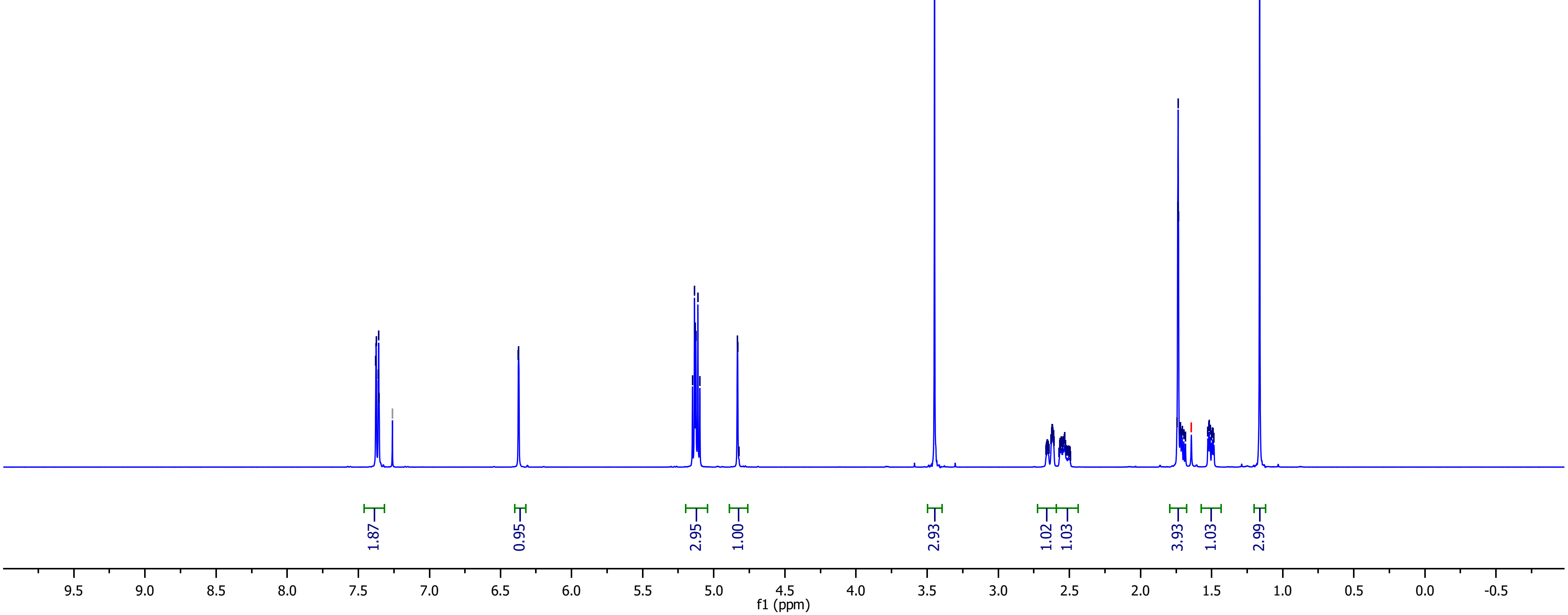

Parameter	Value
Instrument	Avance NEO 500
Solvent	CDCl3
Experiment	1D
Spectrometer Frequency	500.30
Nucleus	1H

JNH_04_76.4.fid — 3 furyl recry mom protected

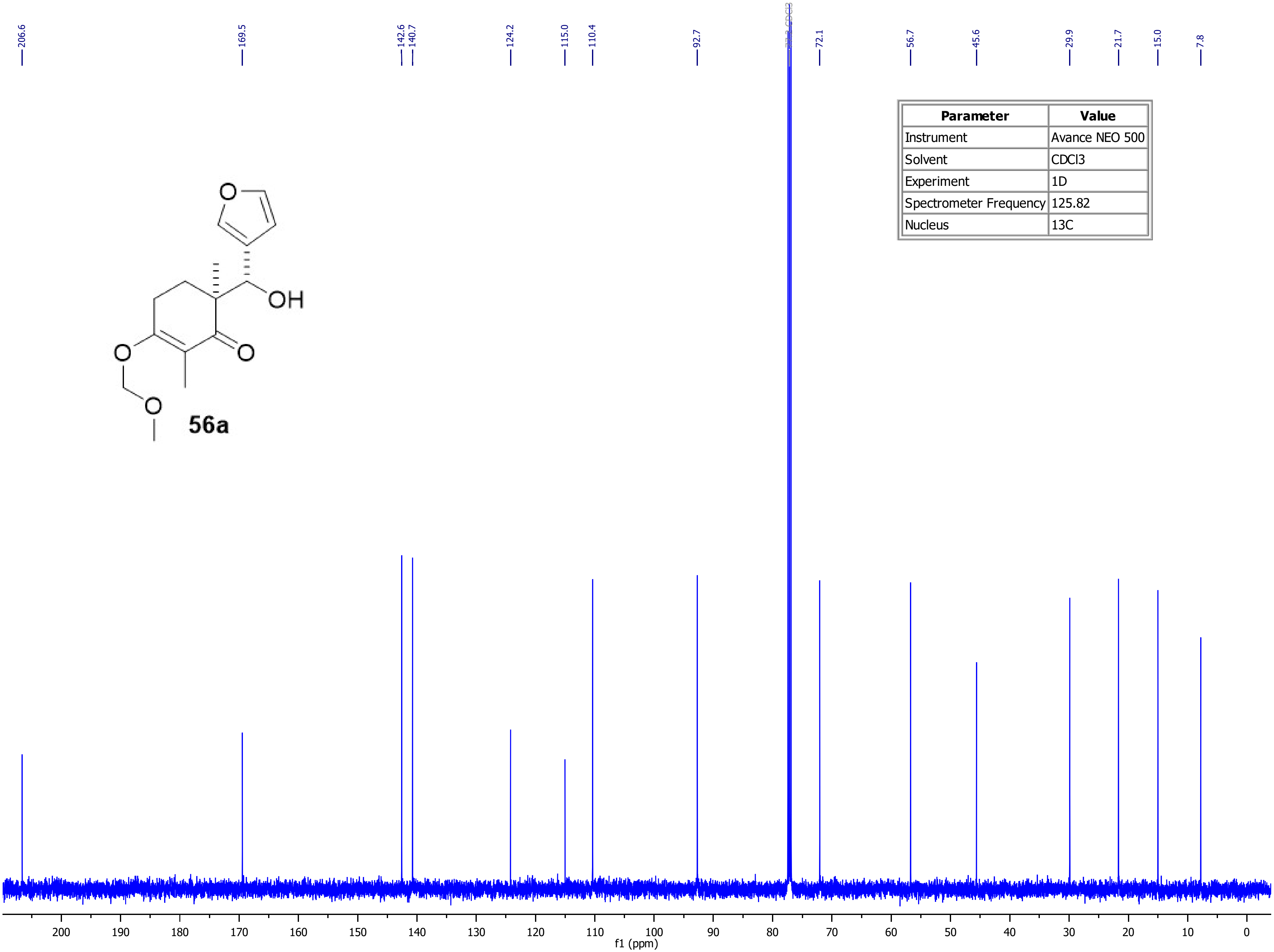

56a

Parameter	Value
Instrument	Avance NEO 500
Solvent	CDCl3
Experiment	1D
Spectrometer Frequency	125.82
Nucleus	13C

JNH_04_76 13C.4.fid — 3 furyl recry mom protected

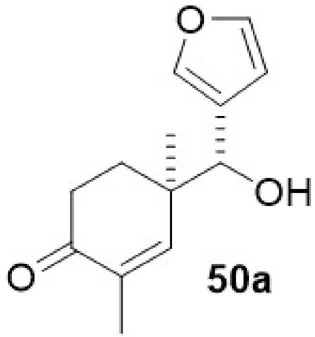

50a

Parameter	Value
Instrument	Avance NEO 500
Solvent	CDCl3
Experiment	1D
Spectrometer Frequency	500.30
Nucleus	1H

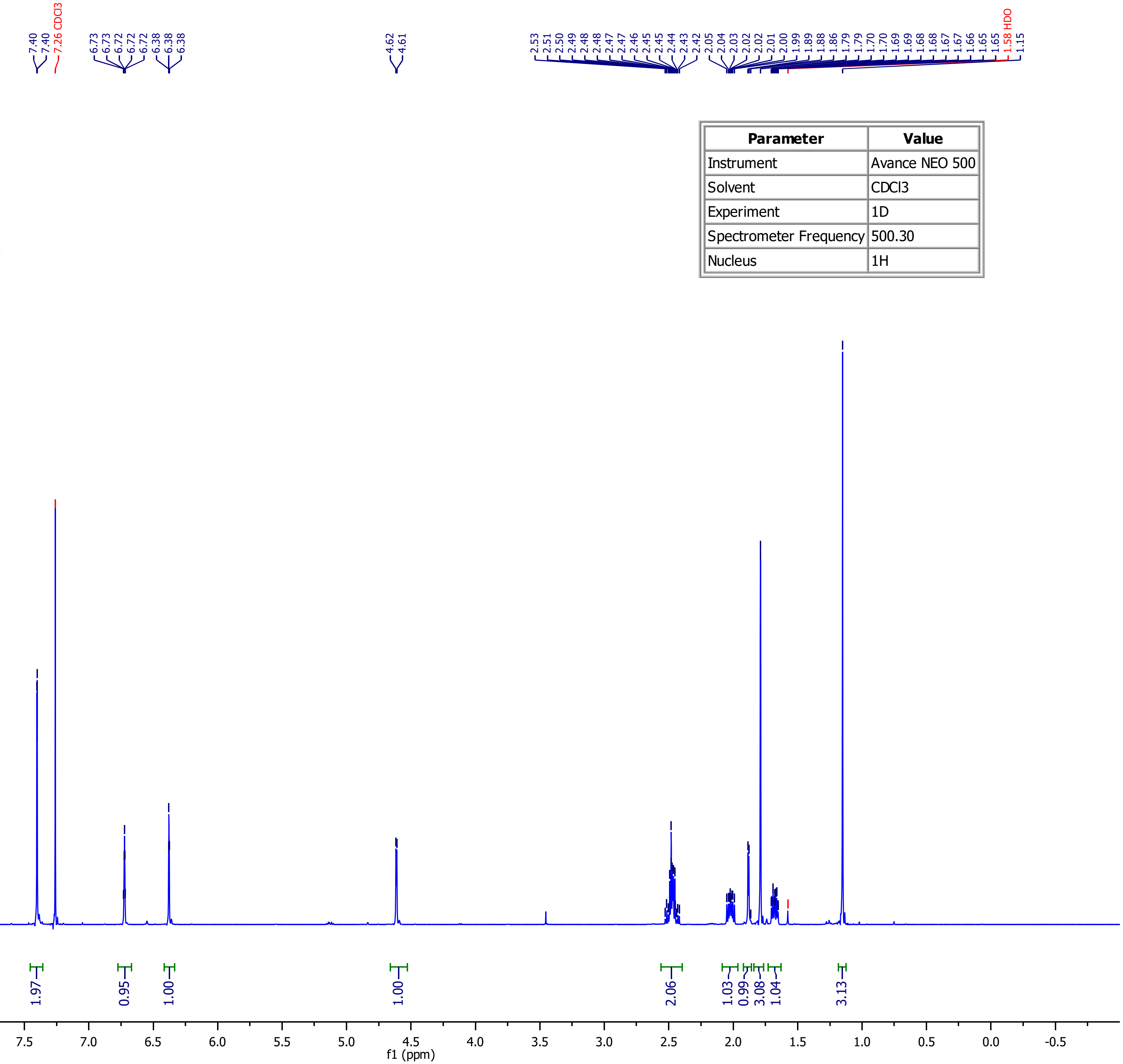

JNH_04_55.1.fid — 3-furyl stork danheiser liminoid product (got a little toasty on hivac but pure via fractions before heating)

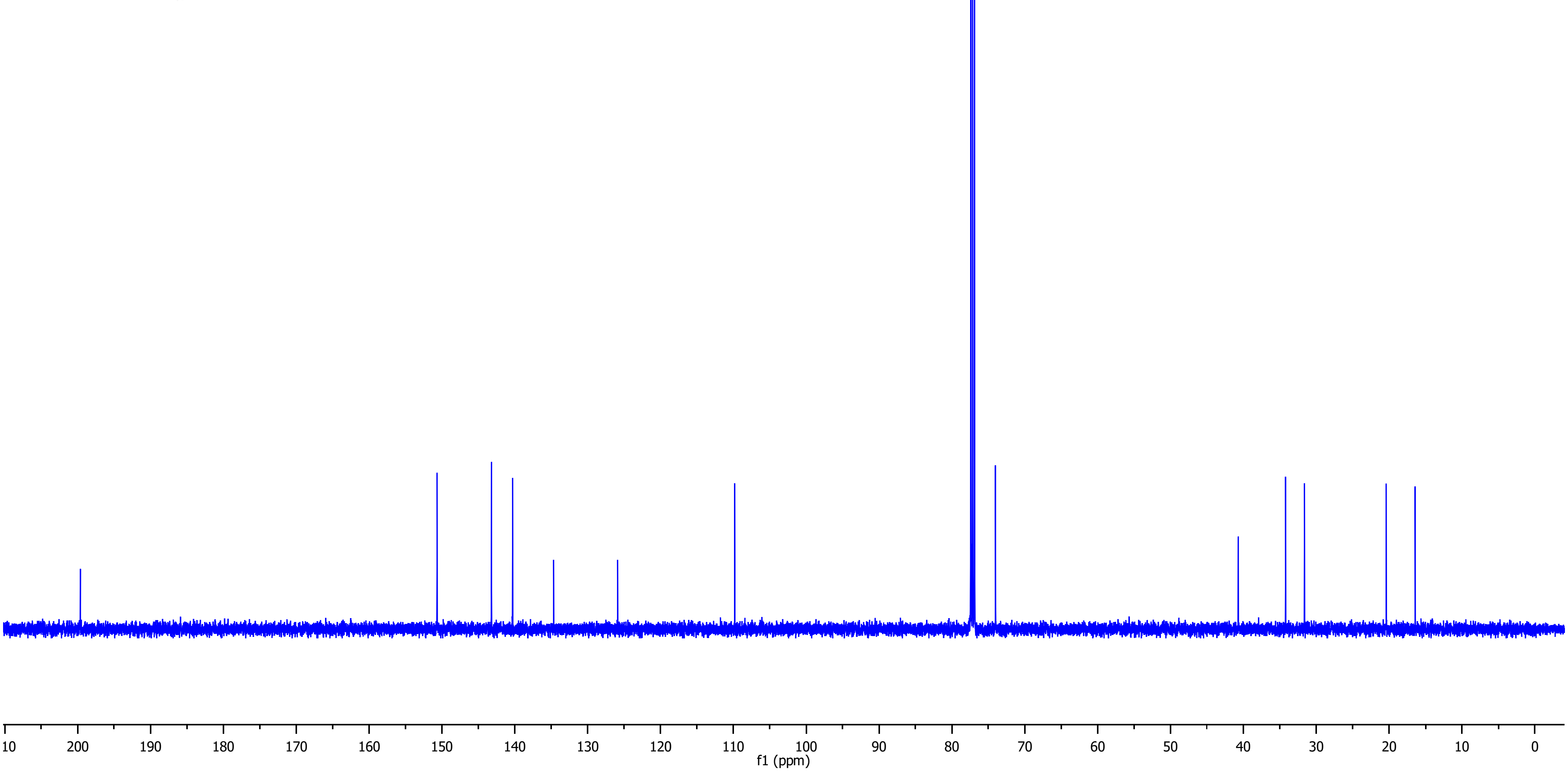

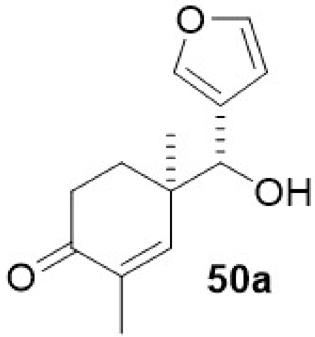

50a

Parameter	Value
Instrument	Avance NEO 500
Solvent	CDCl3
Experiment	1D
Spectrometer Frequency	125.82
Nucleus	13C

JNH_04_55 13C.1.fid — 3-furyl stork danheiser liminoid product (white solid)

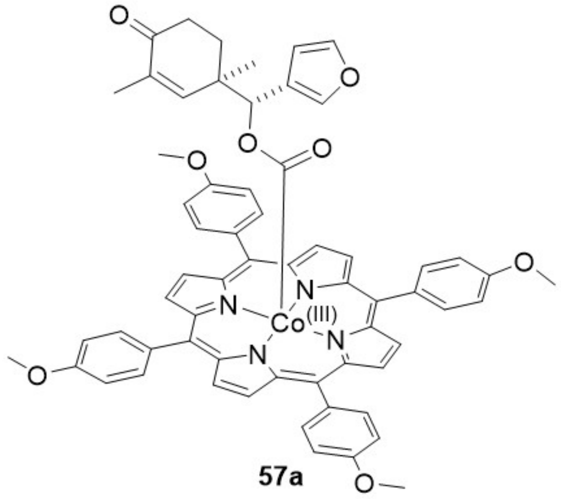

57a

Parameter	Value
Instrument	spect
Solvent	CDCl3
Experiment	1D
Spectrometer Frequency	600.21
Nucleus	1H

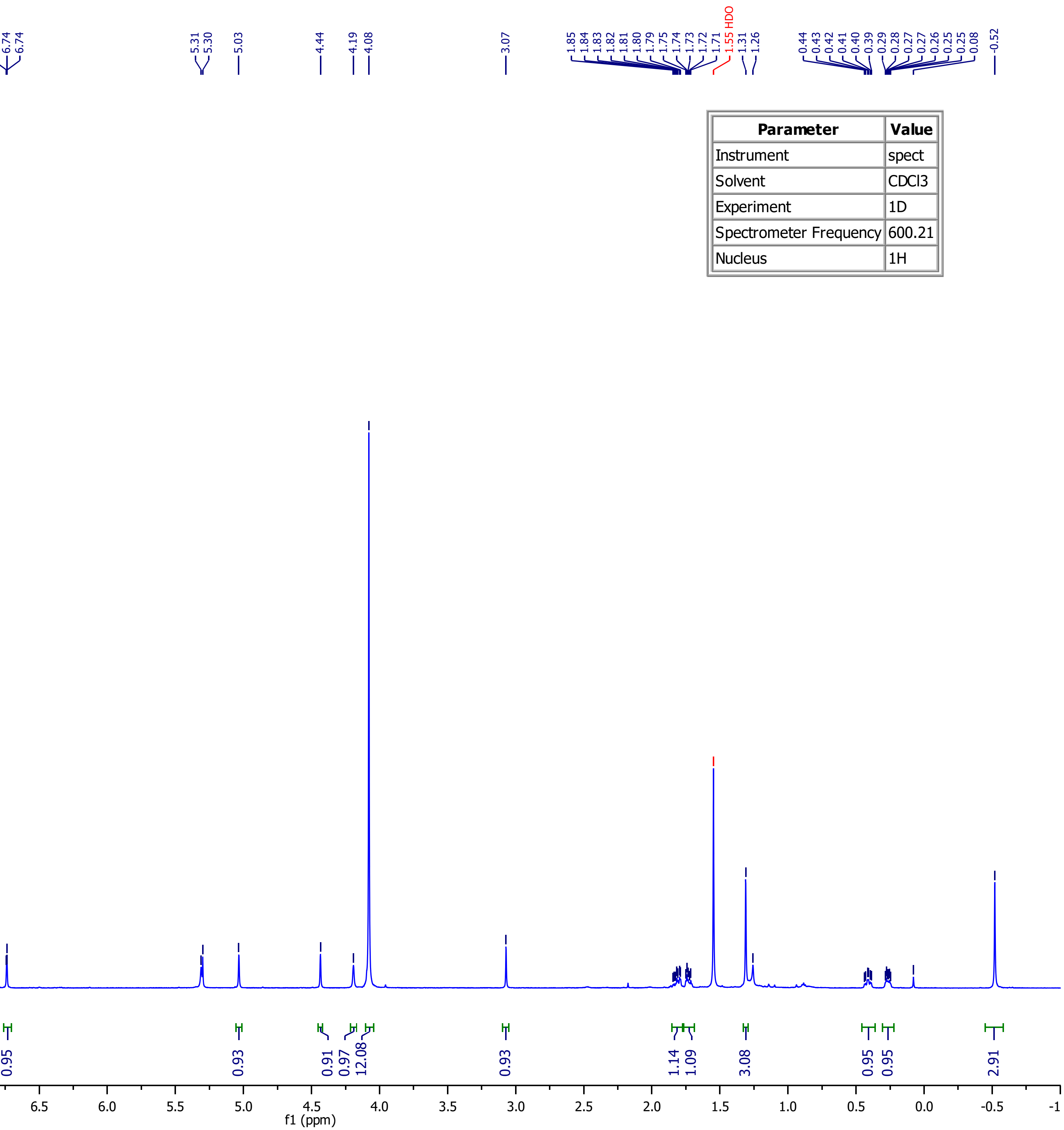

JNH_04_113.1.fid — 3-furanyl TAP carbonylation product limonoid (9.4 mg)

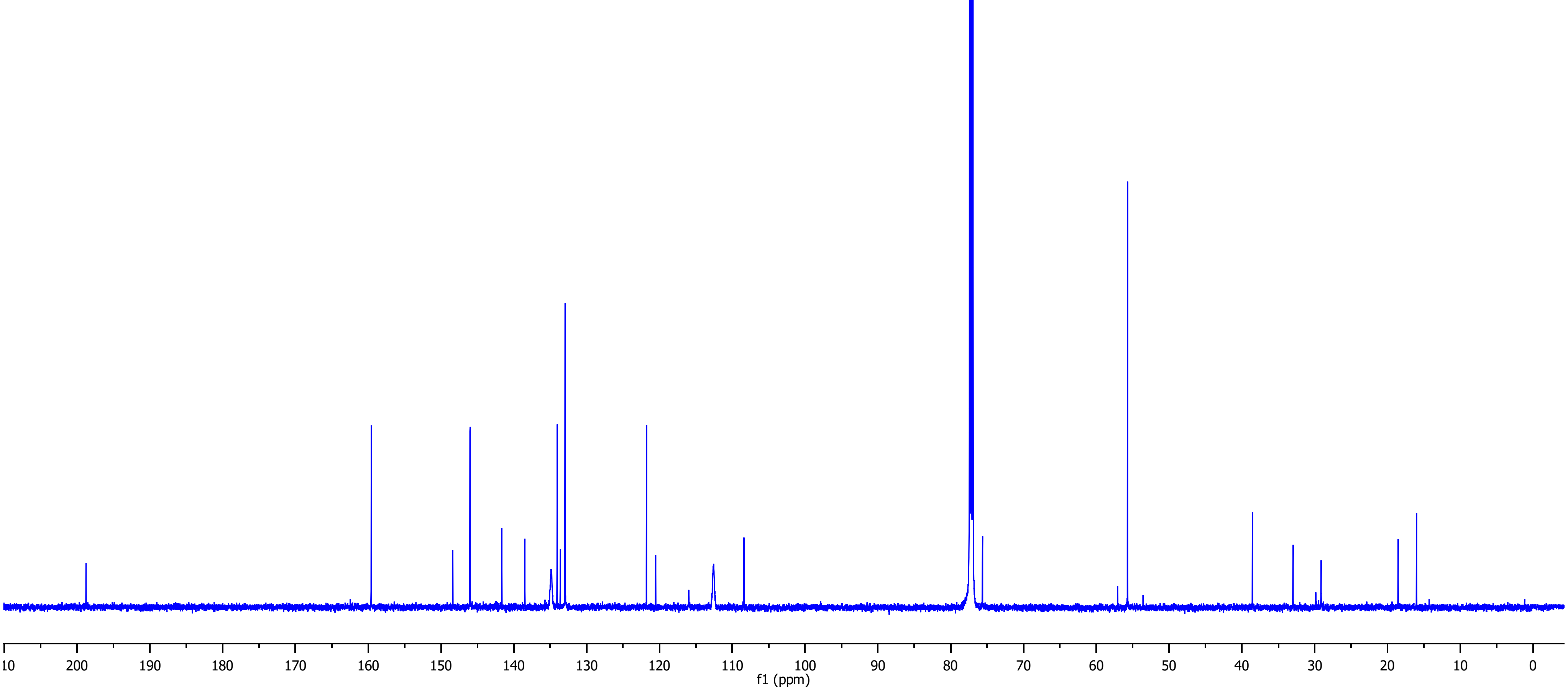

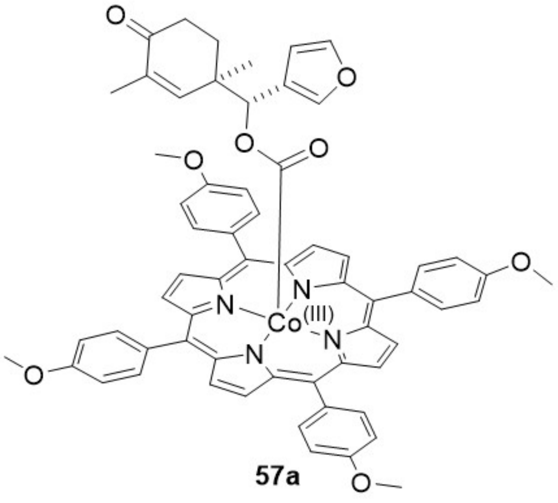

57a

Parameter	Value
Instrument	spect
Solvent	CDCl3
Experiment	1D
Spectrometer Frequency	150.94
Nucleus	13C

JNH_04_113 13C.1.fid — 3-furanyl liminoid precursor exp 1 13C

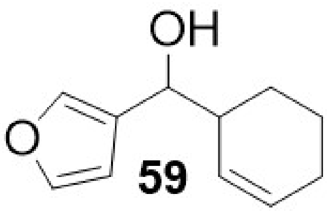

59

Parameter	Value
Instrument	Avance NEO 500
Solvent	CDCl3
Experiment	1D
Spectrometer Frequency	500.30
Nucleus	1H

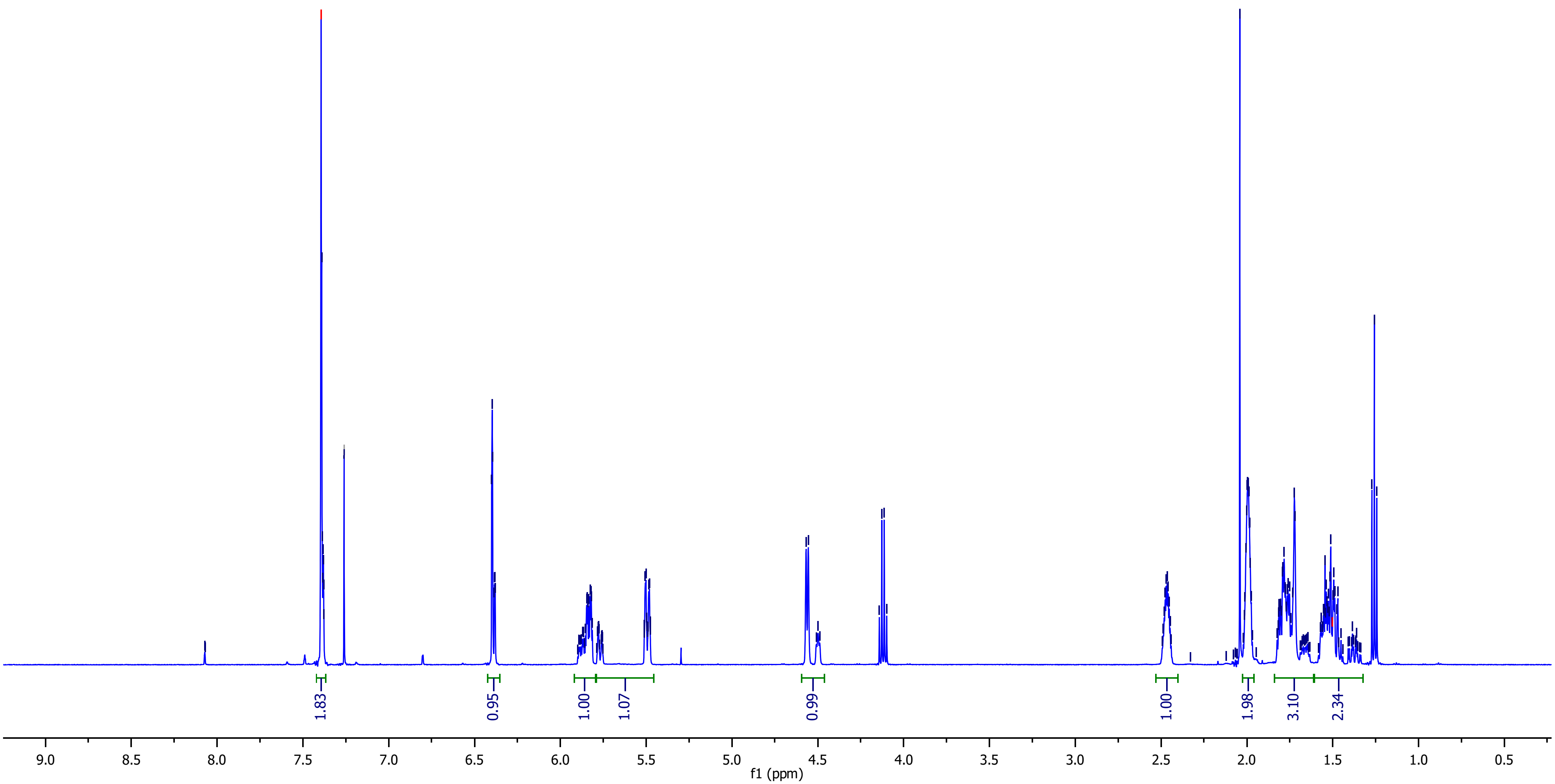

JNH_04_185.1.fid — 3-Furyl Barbier with cyclohexene

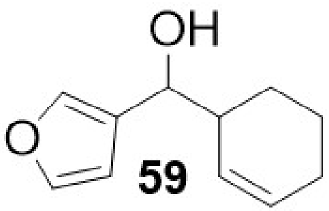

59

Parameter	Value
Instrument	spect
Solvent	CDCl3
Experiment	1D
Spectrometer Frequency	150.94
Nucleus	13C

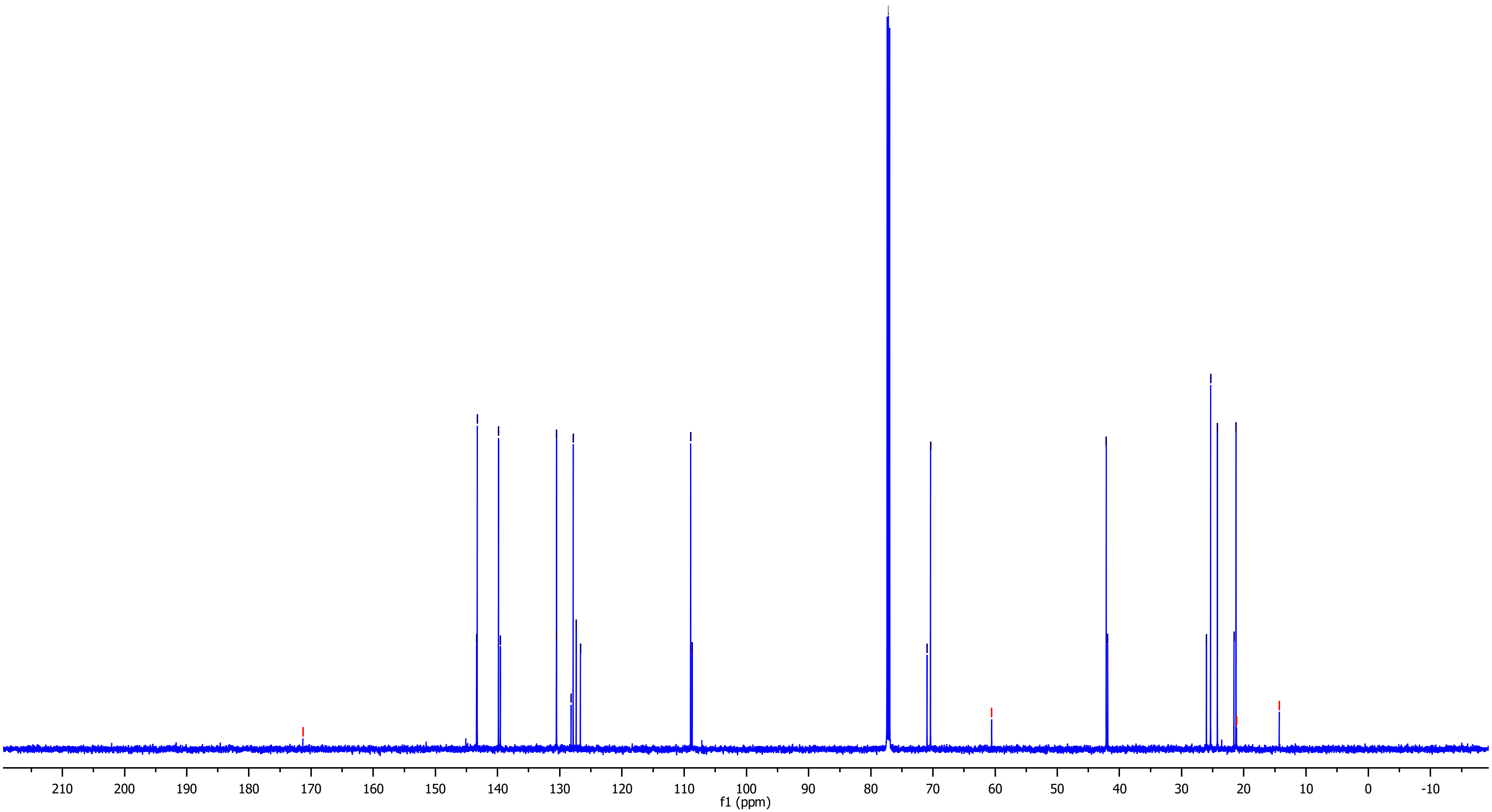

JNH_04_185 13C.2.fid — Barbier reaction

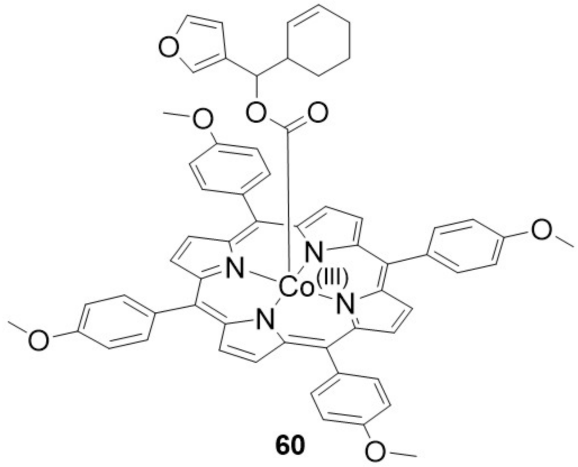

60

Parameter	Value
Instrument	Avance NEO 500
Solvent	CDCl3
Experiment	1D
Spectrometer Frequency	500.30
Nucleus	1H

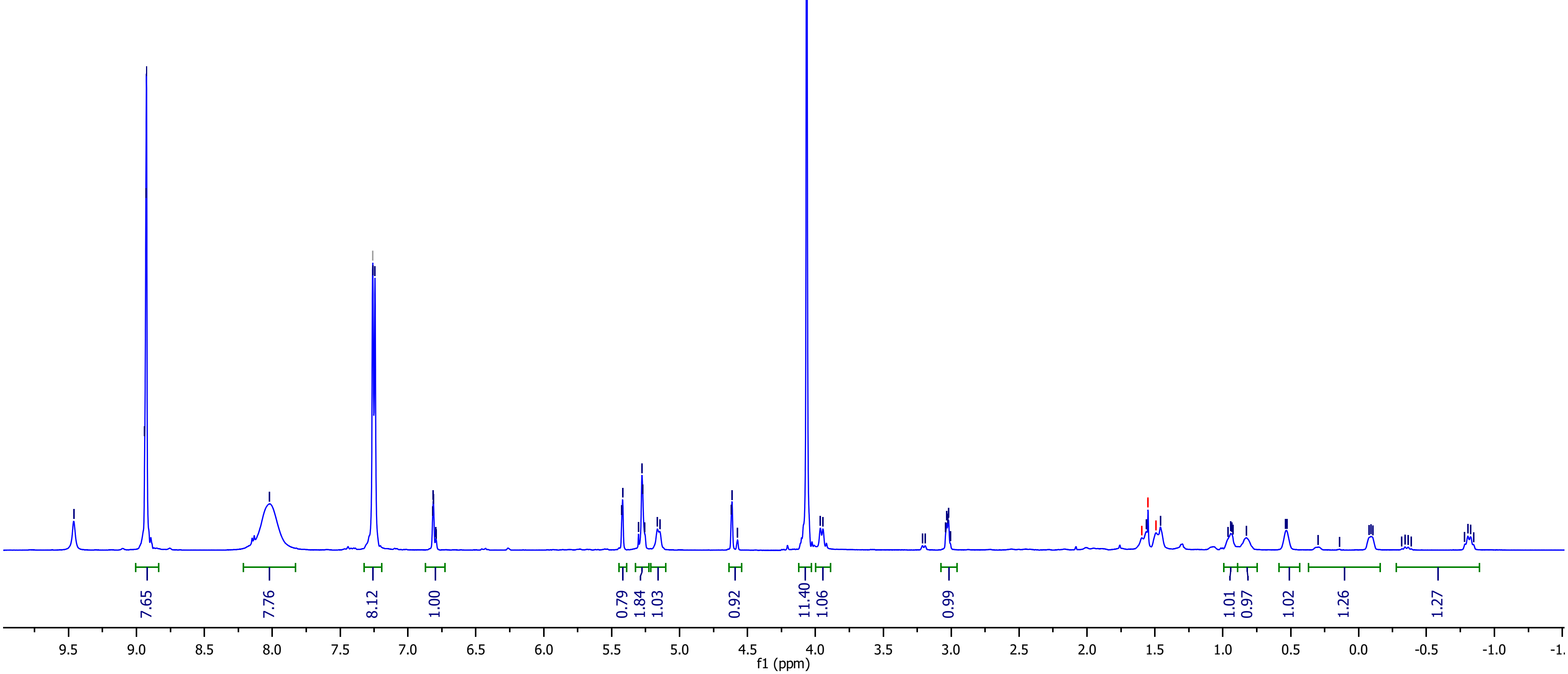

JNH_04_186.1.fid — 3-furyl cylcohexene carbonylation product

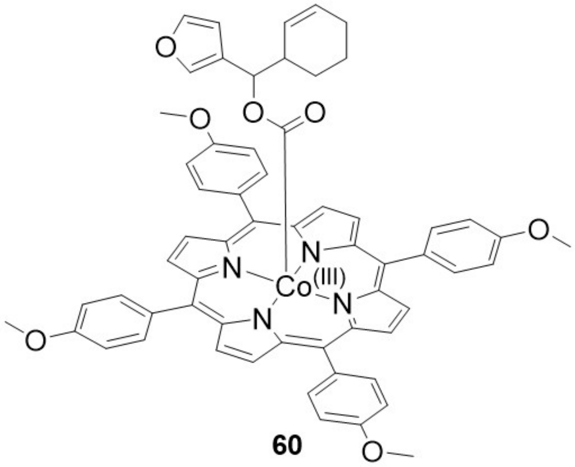

60

Parameter	Value
Instrument	Avance NEO 500
Solvent	CDCl3
Experiment	1D
Spectrometer Frequency	125.82
Nucleus	13C

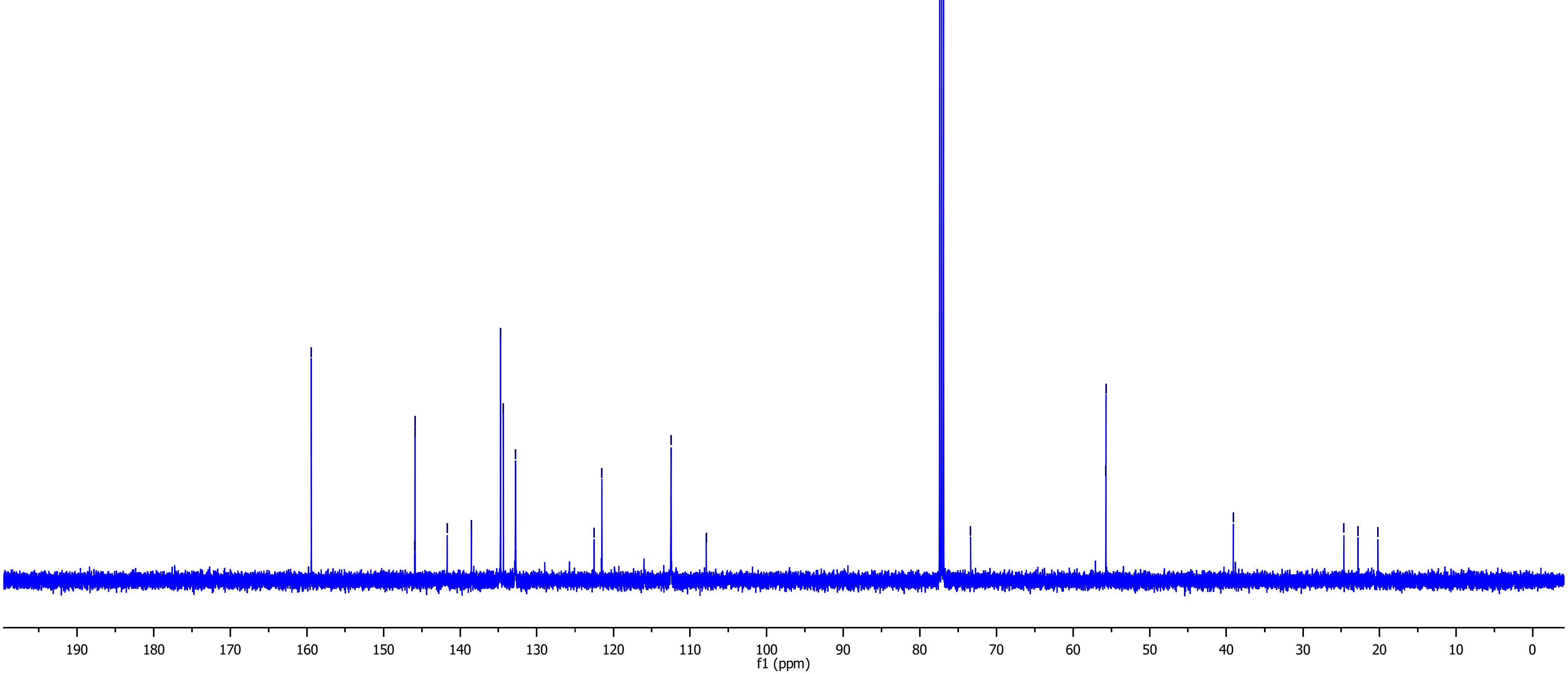

JNH_04_186 13C.1.fid — 3 furyl cyclohexene carbonylated product

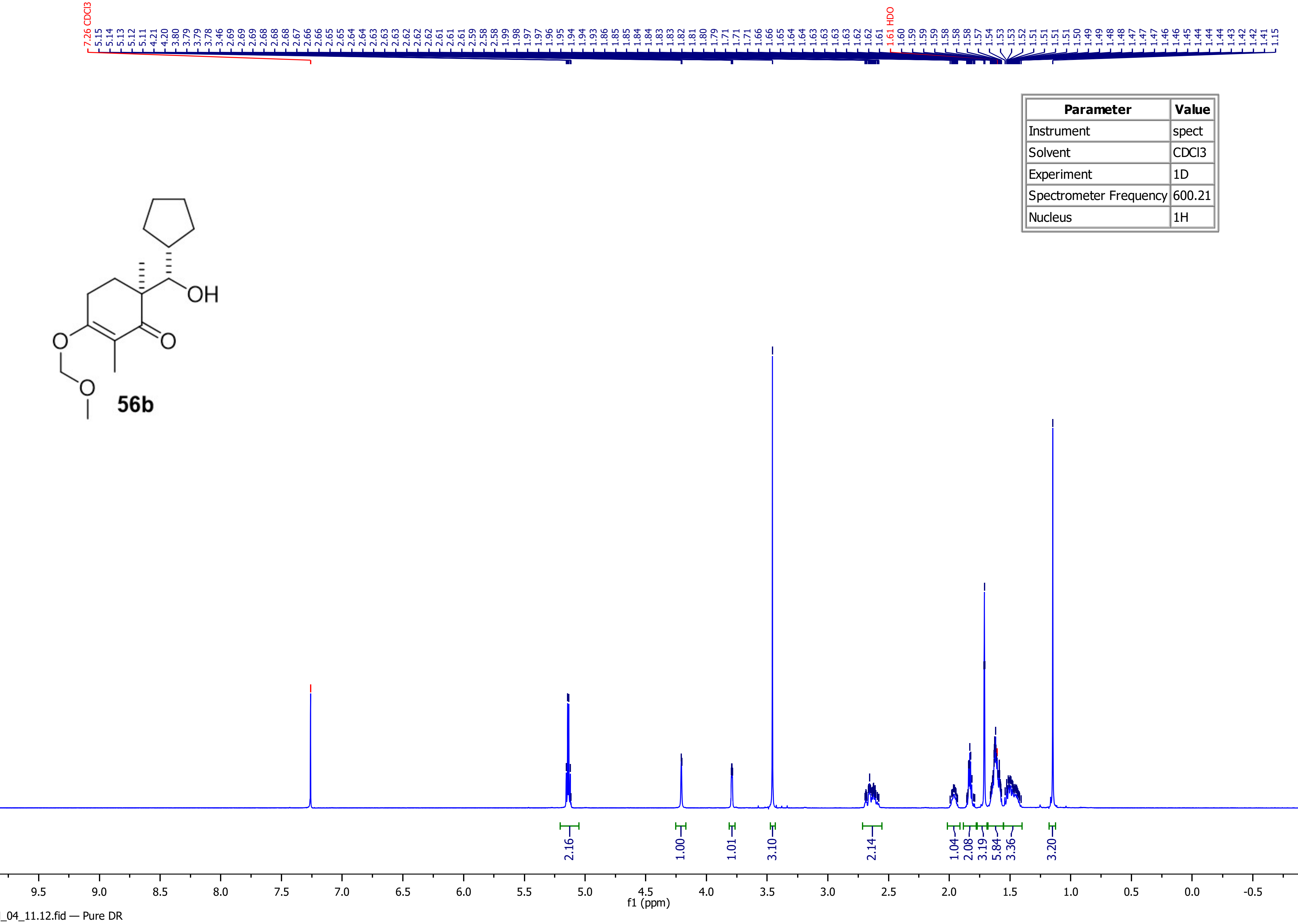

Parameter	Value
Instrument	spect
Solvent	CDCl3
Experiment	1D
Spectrometer Frequency	600.21
Nucleus	1H

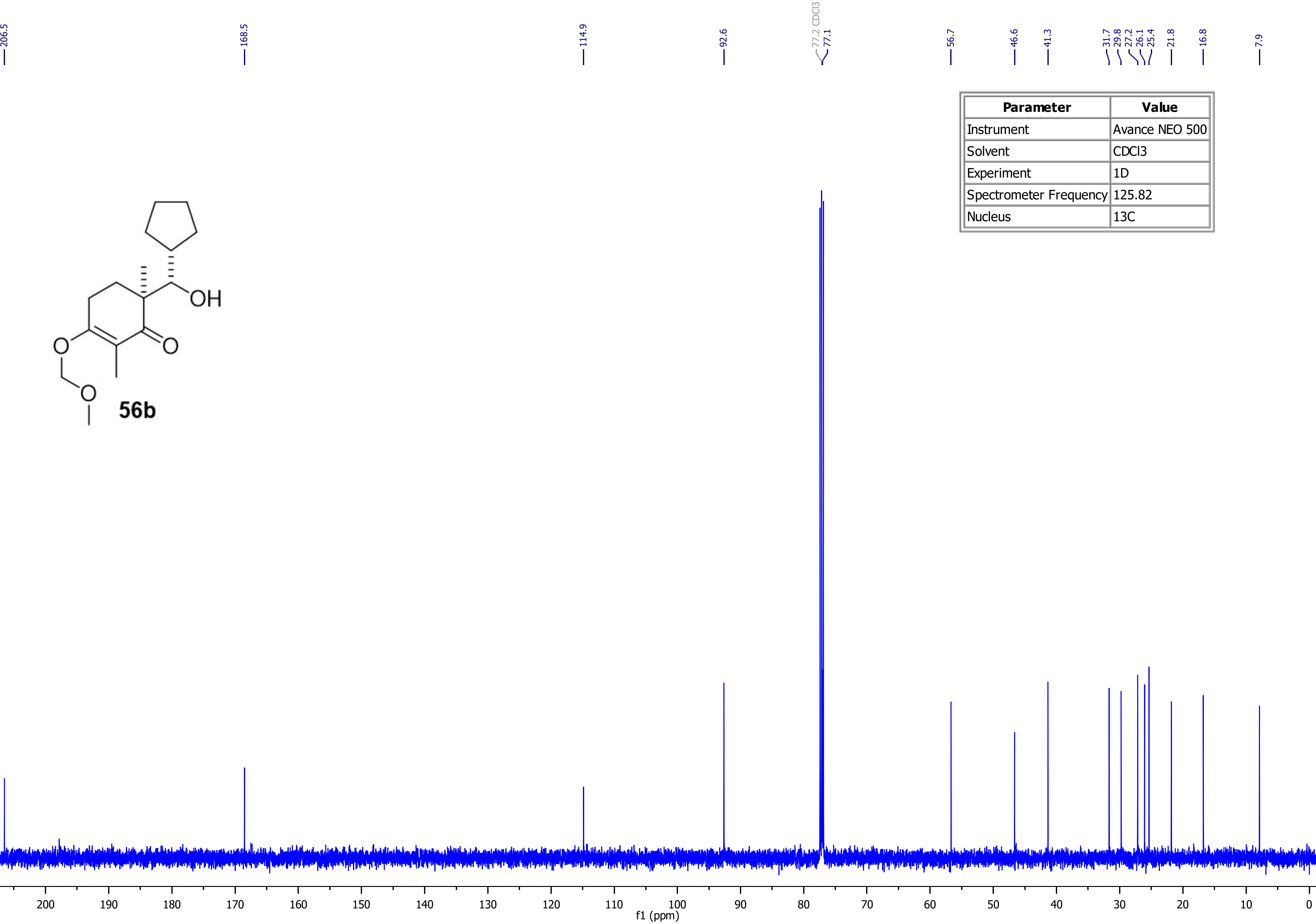

Parameter	Value
Instrument	Avance NEO 500
Solvent	CDCl3
Experiment	1D
Spectrometer Frequency	125.82
Nucleus	13C

_04_11 13C.14.fid — Pure Aldol Diastereomer

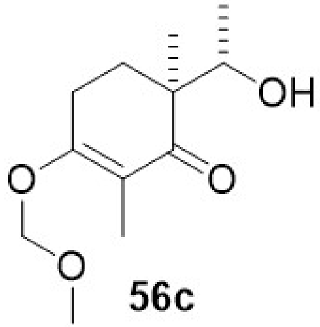

56c

Parameter	Value
Instrument	Avance
Solvent	CDCl3
Experiment	1D
Spectrometer Frequency	400.30
Nucleus	1H

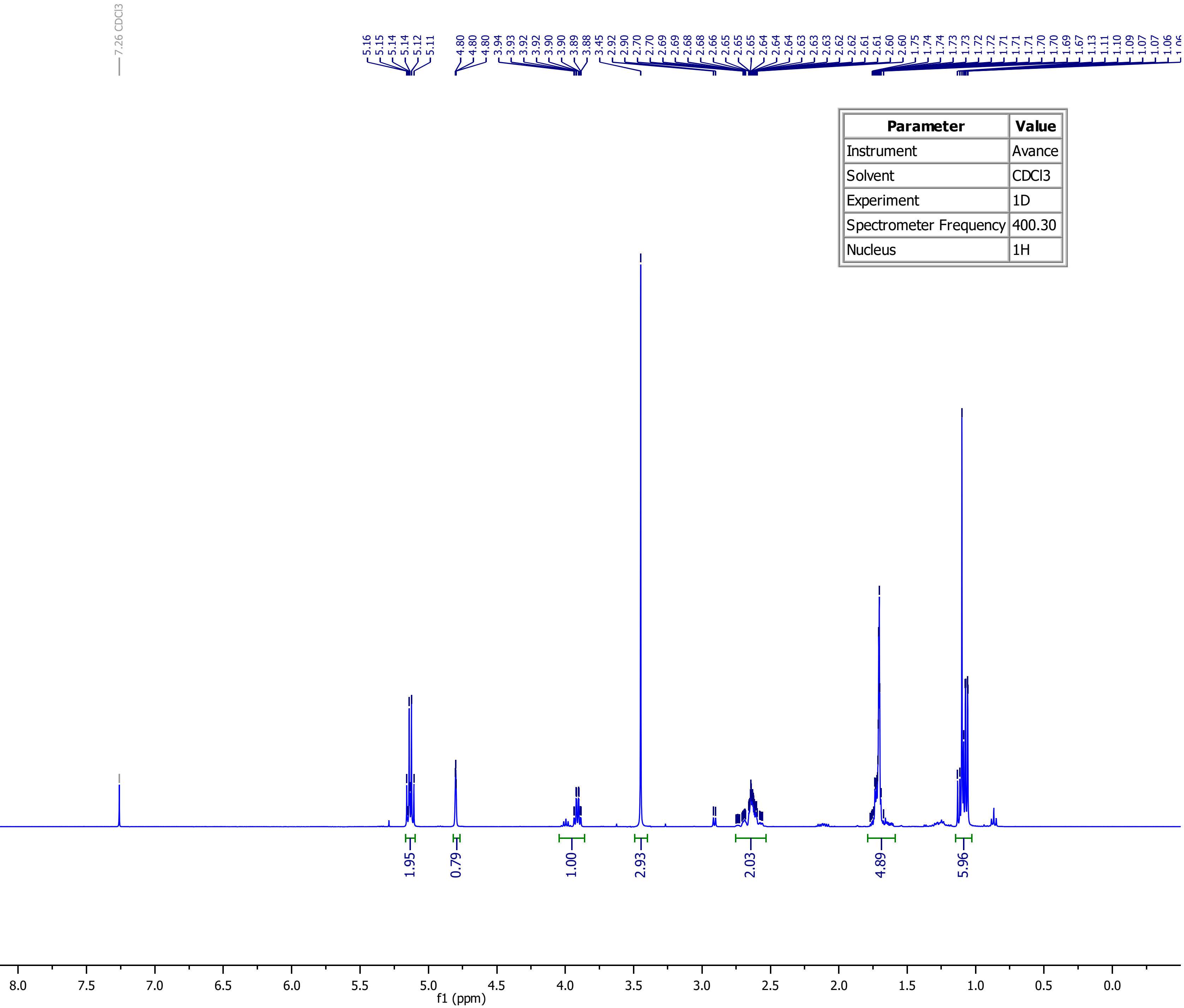

JNH_04_18.5.fid — Acetyl aldehyde aldol Liminoid adduct (0.9985 g, 86%)

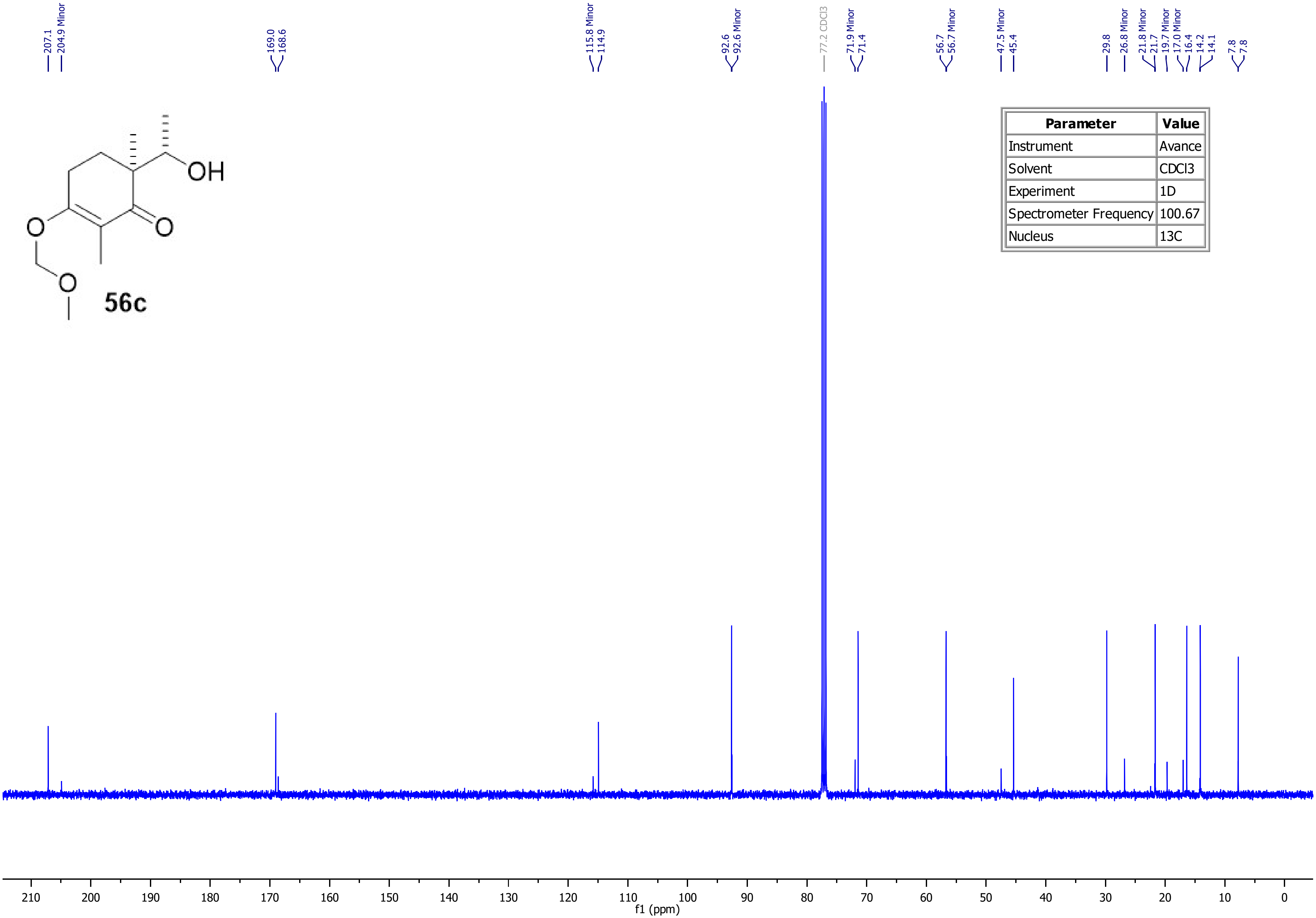

Parameter	Value
Instrument	Avance
Solvent	CDCl3
Experiment	1D
Spectrometer Frequency	100.67
Nucleus	13C

JNH_04_18 13C.5.fid — Acetyl aldehyde aldol Liminoid adduct (0.9985 g, 86%)

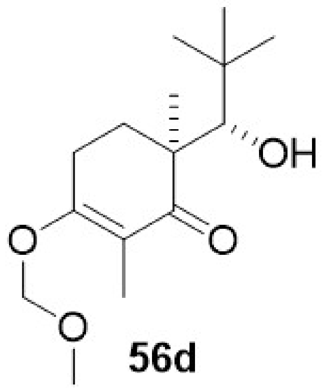

56d

Parameter	Value
Instrument	Avance
Solvent	CDCl3
Experiment	1D
Spectrometer Frequency	400.30
Nucleus	1H

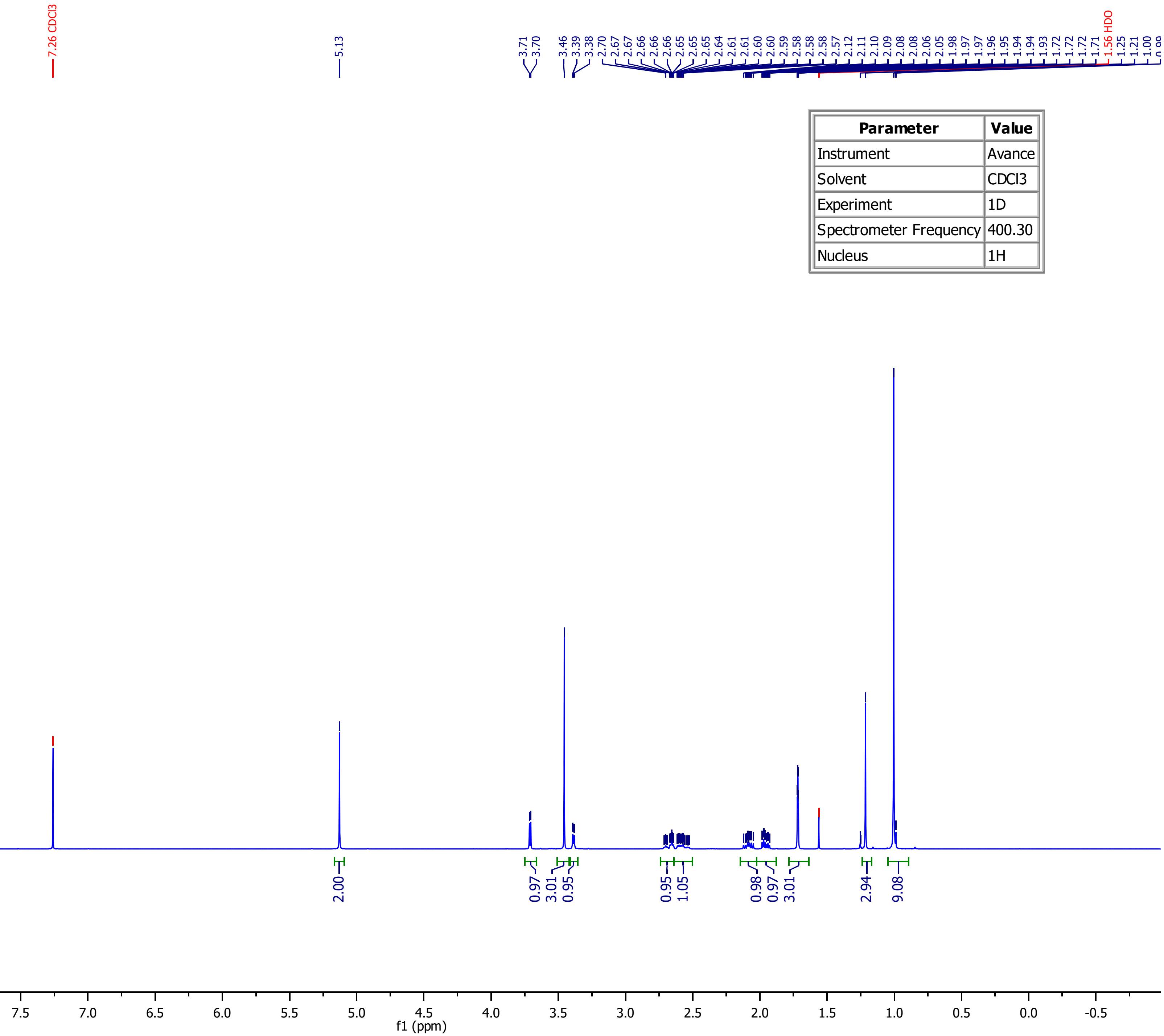

JNH_04_19.3.fid — Pivaldehyde aldol Liminoid adduct middle fraction (2.7.3)

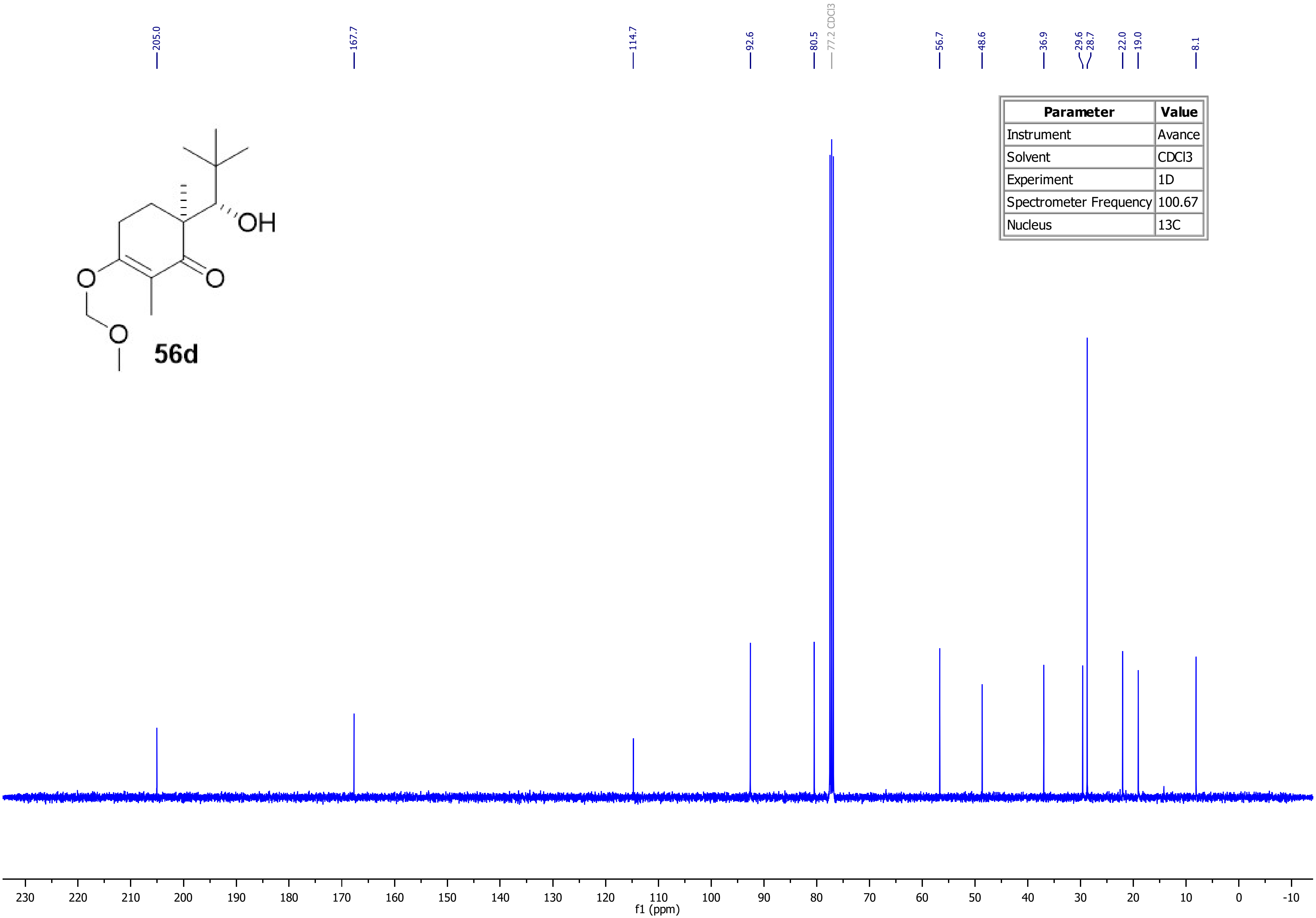

56d

Parameter	Value
Instrument	Avance
Solvent	CDCl3
Experiment	1D
Spectrometer Frequency	100.67
Nucleus	13C

JNH_04_19 13C.5.fid — Pivaldehyde aldol Liminoid adduct (0.4866 g, 31%)

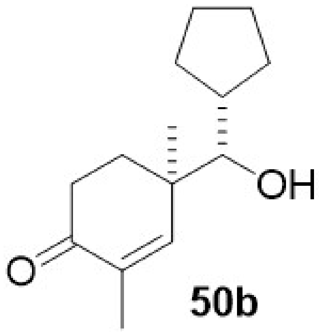

50b

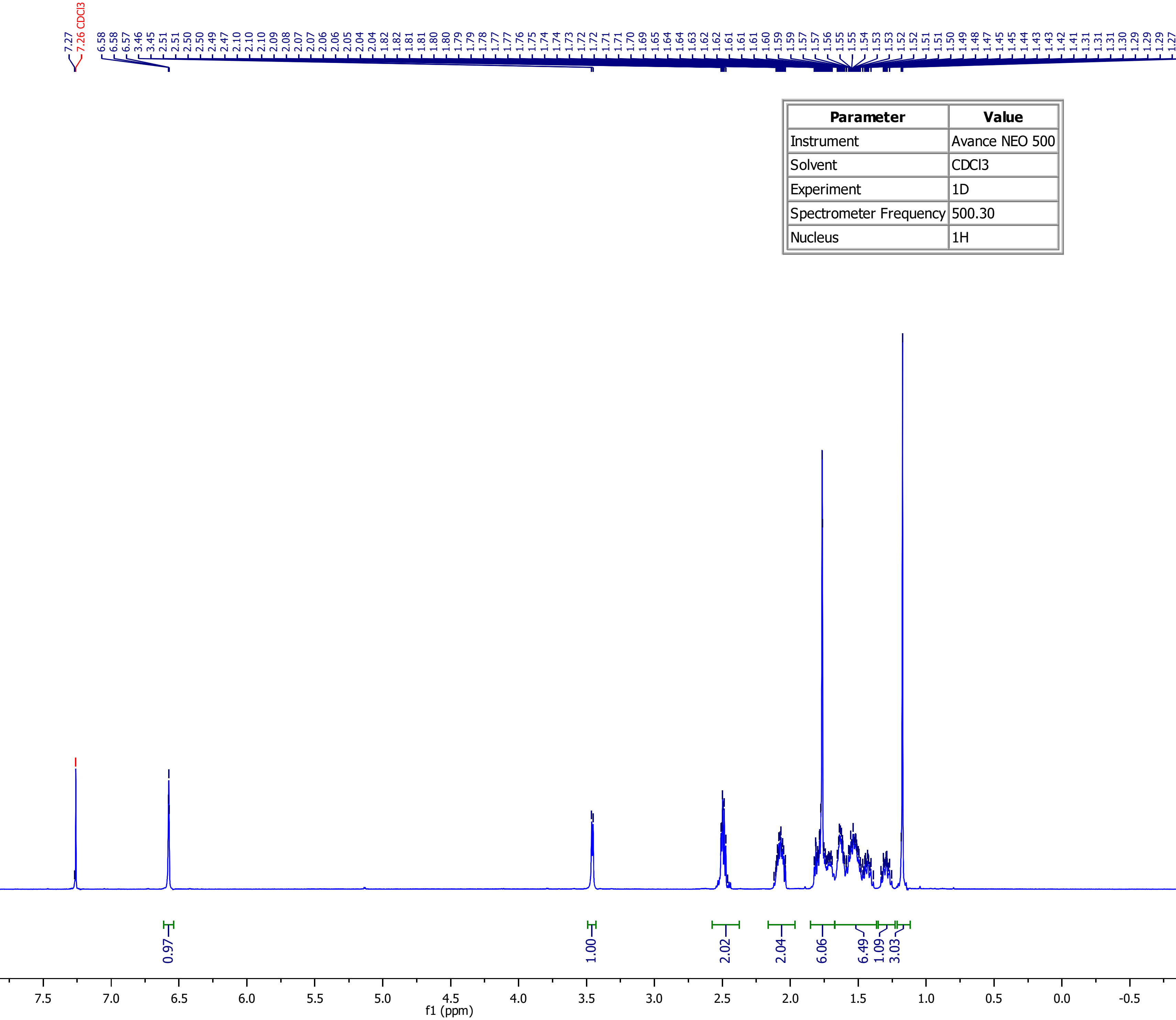

Parameter	Value
Instrument	Avance NEO 500
Solvent	CDCl3
Experiment	1D
Spectrometer Frequency	500.30
Nucleus	1H

_04_12.3.fid — Stork Danheiser Major spot (UV active)

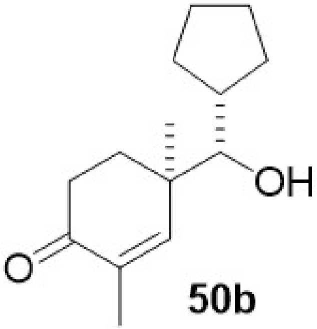

50b

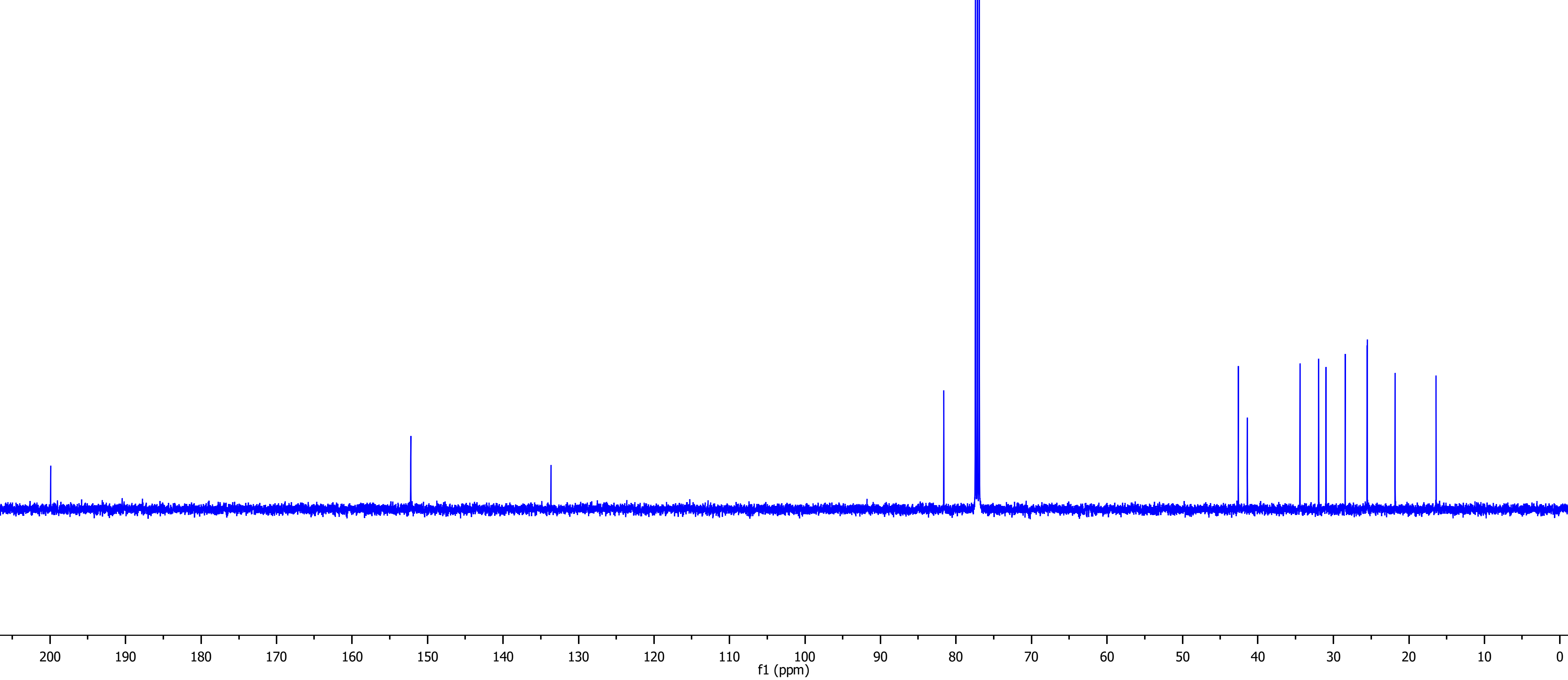

Parameter	Value
Instrument	Avance NEO 500
Solvent	CDCl3
Experiment	1D
Spectrometer Frequency	125.82
Nucleus	13C

_04_12 13C.3.fid — Stork Danheiser major product 13C

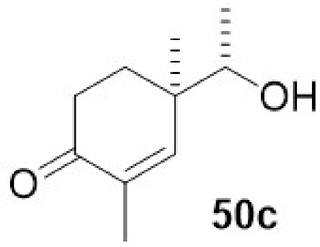

50c

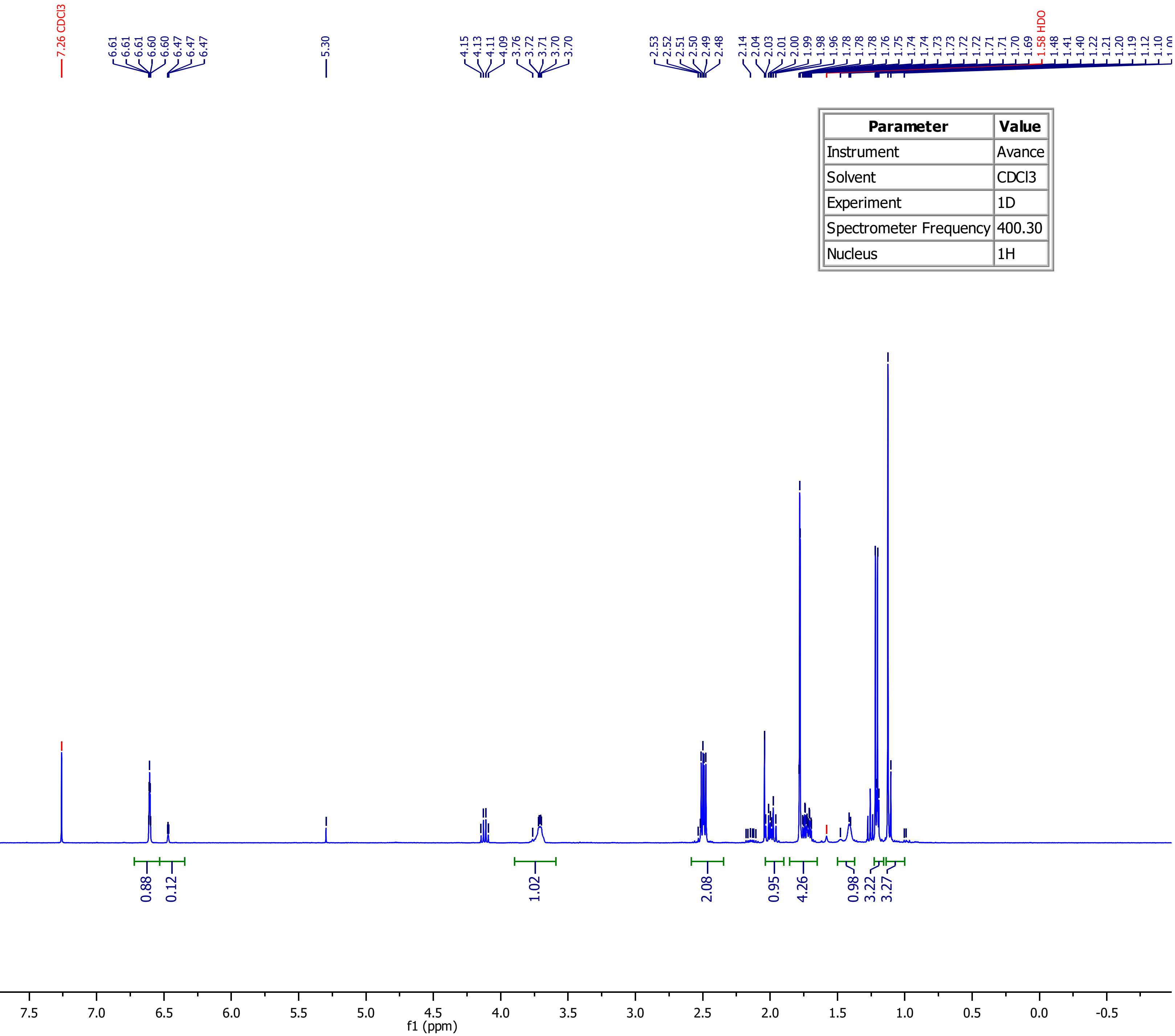

Parameter	Value
Instrument	Avance
Solvent	CDCl3
Experiment	1D
Spectrometer Frequency	400.30
Nucleus	1H

JNH_04_180.2.fid — Main Peak 1:1 etoac:hex for SD methyl liminoid

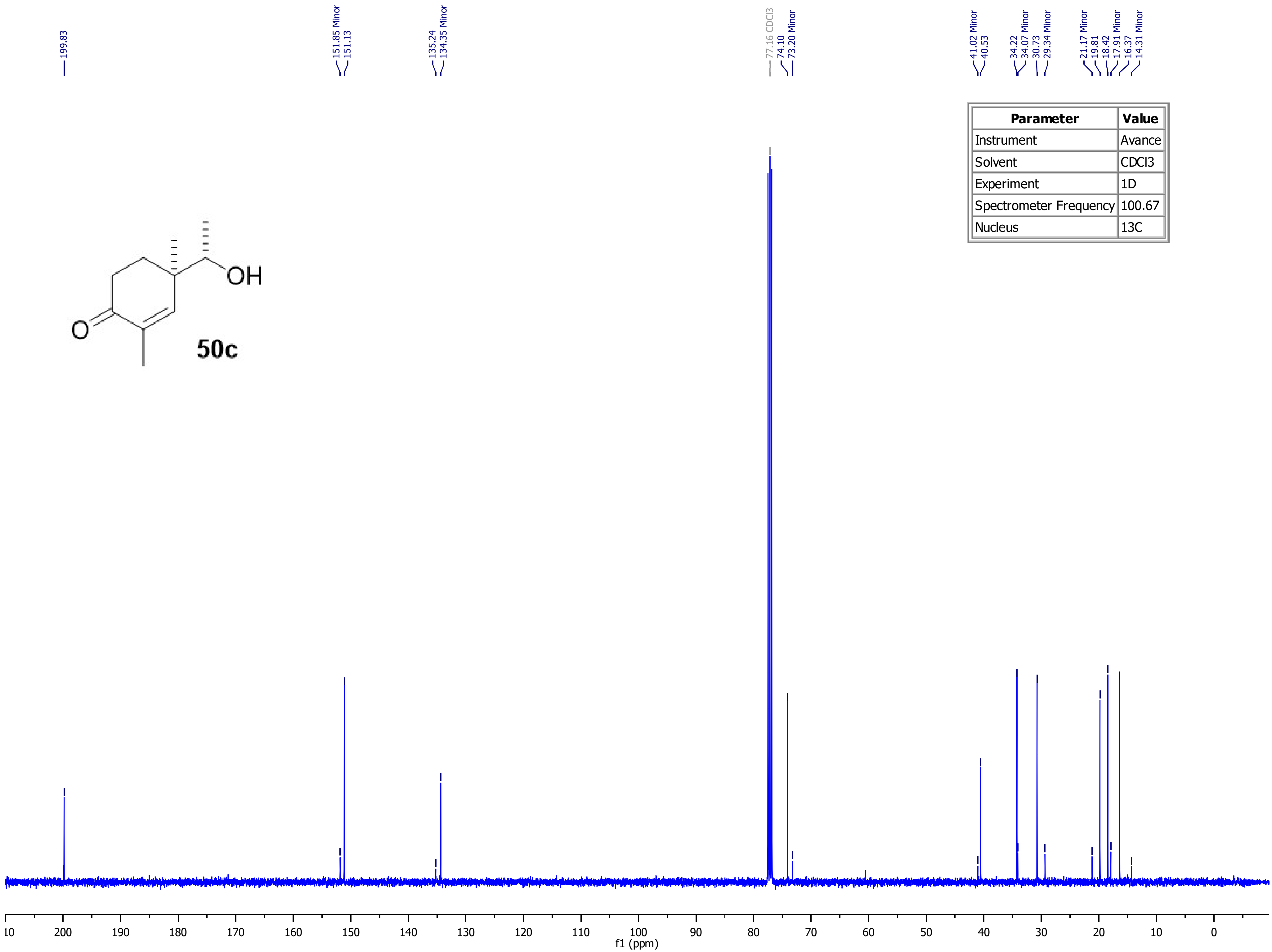

Parameter	Value
Instrument	Avance
Solvent	CDCl3
Experiment	1D
Spectrometer Frequency	100.67
Nucleus	13C

JNH_04_20 13C.4.fid — acetaldehyde stork danheiser mixed with JNH_04_180 as well

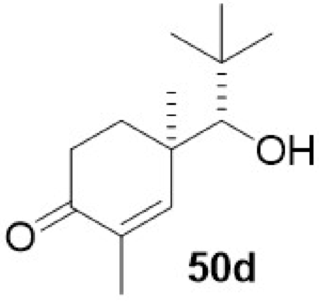

50d

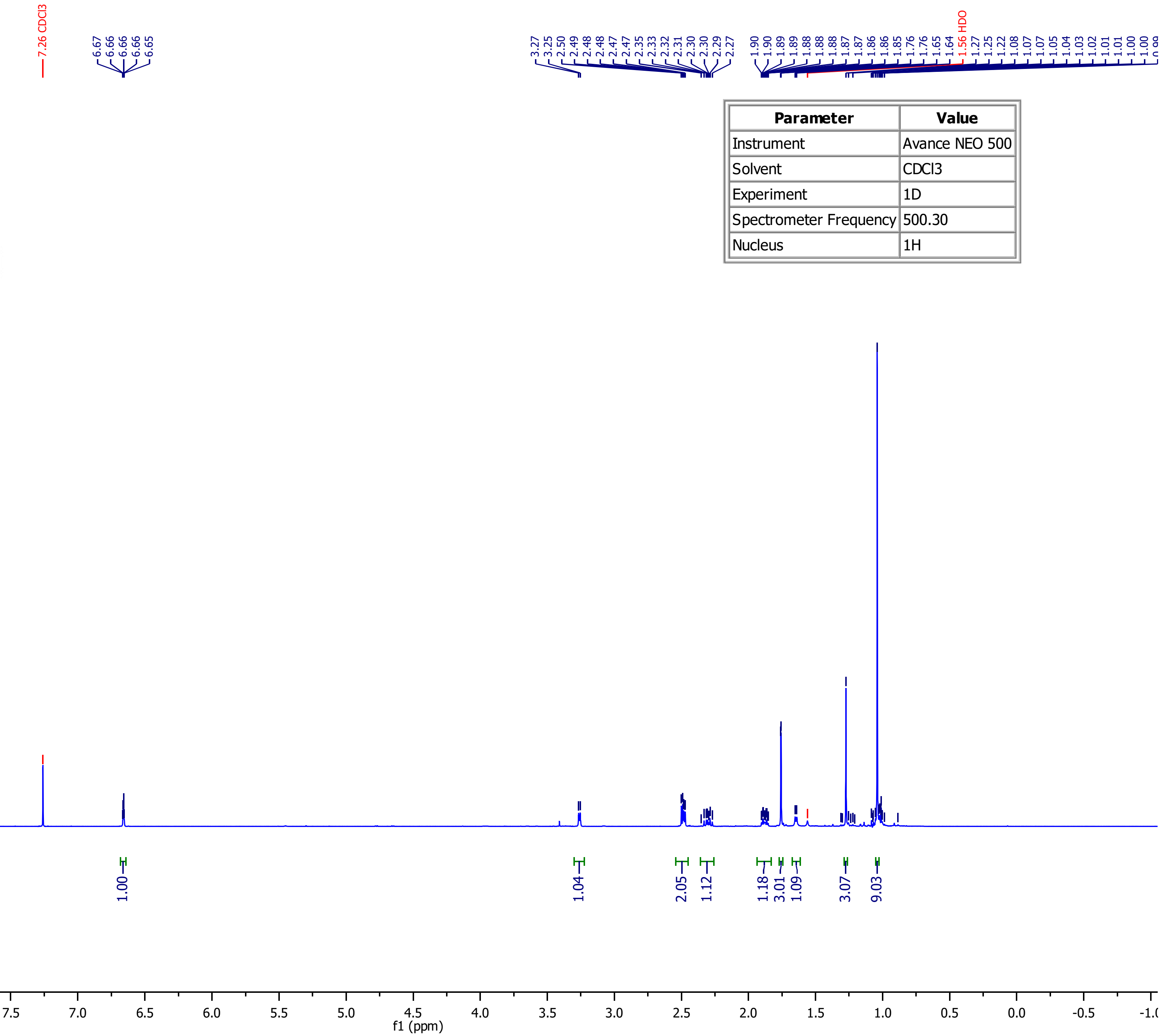

Parameter	Value
Instrument	Avance NEO 500
Solvent	CDCl3
Experiment	1D
Spectrometer Frequency	500.30
Nucleus	1H

JNH_04_21.1.fid — Crude Pivaldehyde SD (0.3681 g, 91% mass balance)

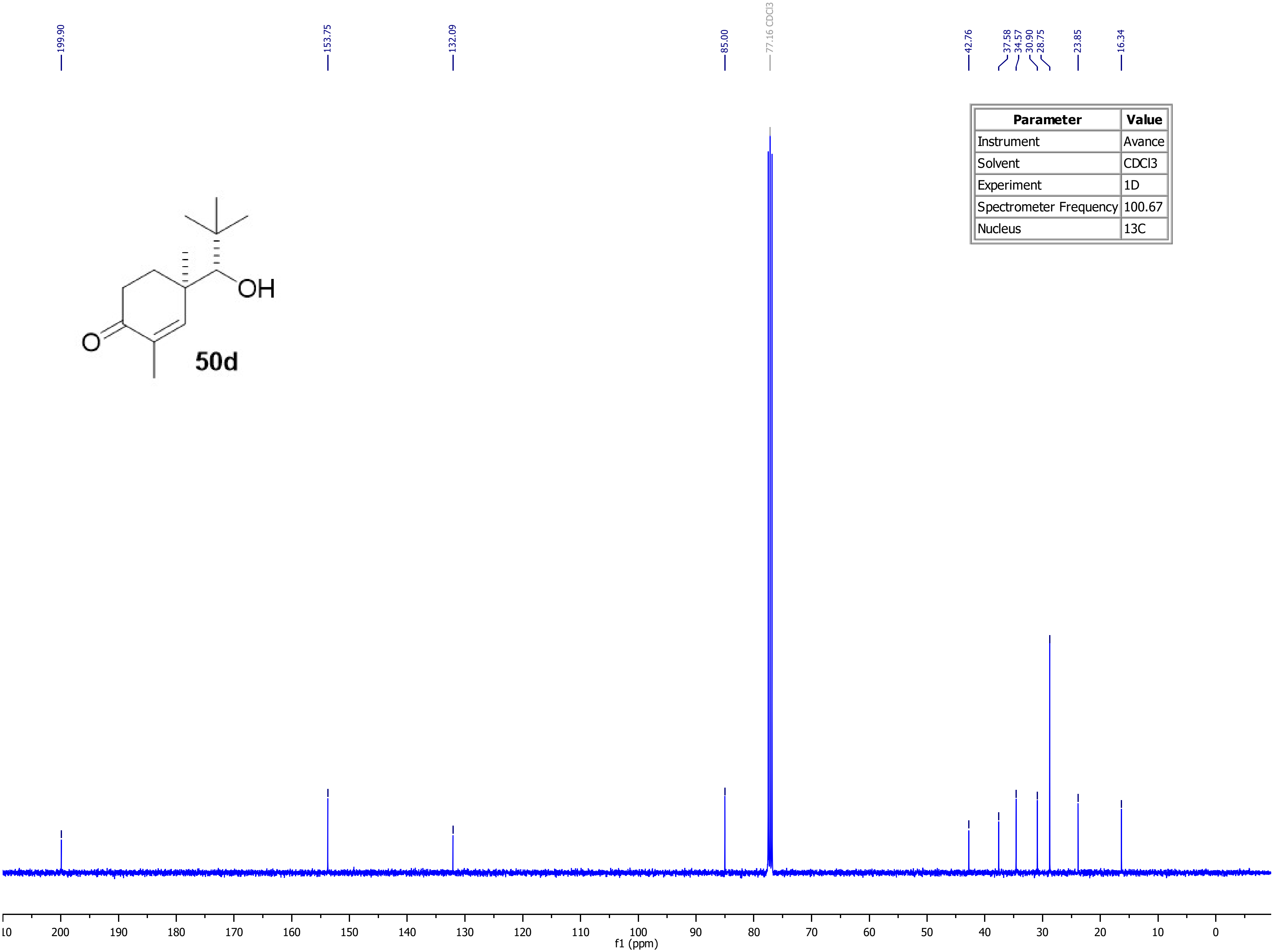

50d

Parameter	Value
Instrument	Avance
Solvent	CDCl3
Experiment	1D
Spectrometer Frequency	100.67
Nucleus	13C

JNH_04_21 13C.4.fid — pivaldehyde stork-danheiser post recrystallization

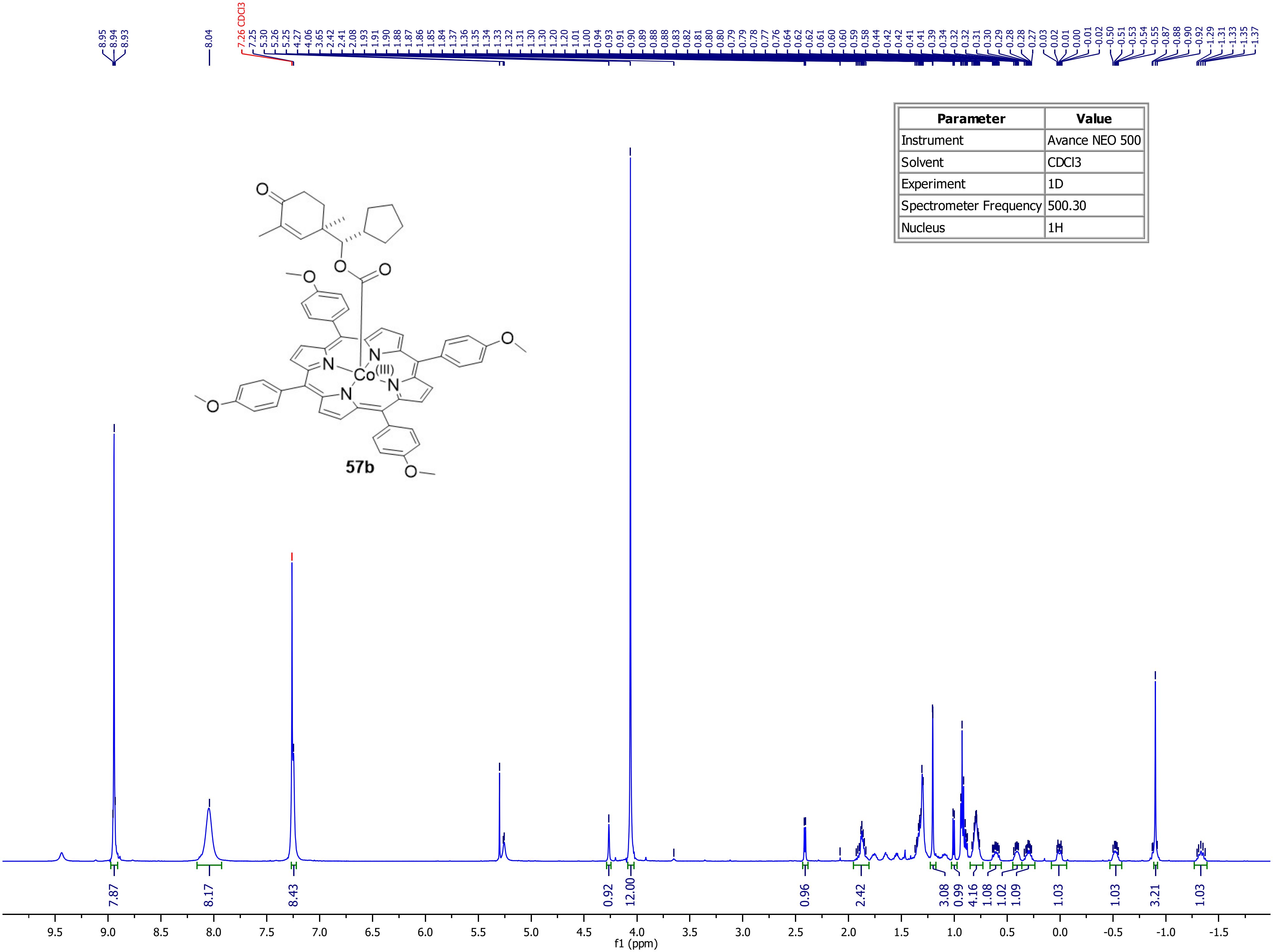

57b

Parameter	Value
Instrument	Avance NEO 500
Solvent	CDCl3
Experiment	1D
Spectrometer Frequency	500.30
Nucleus	1H

JNH_04_52.9.fid — 0.0548 g cyclopentyl limonoid carbonylation product

Parameter	Value
Instrument	Avance
Solvent	CDCl3
Experiment	1D
Spectrometer Frequency	100.67
Nucleus	13C

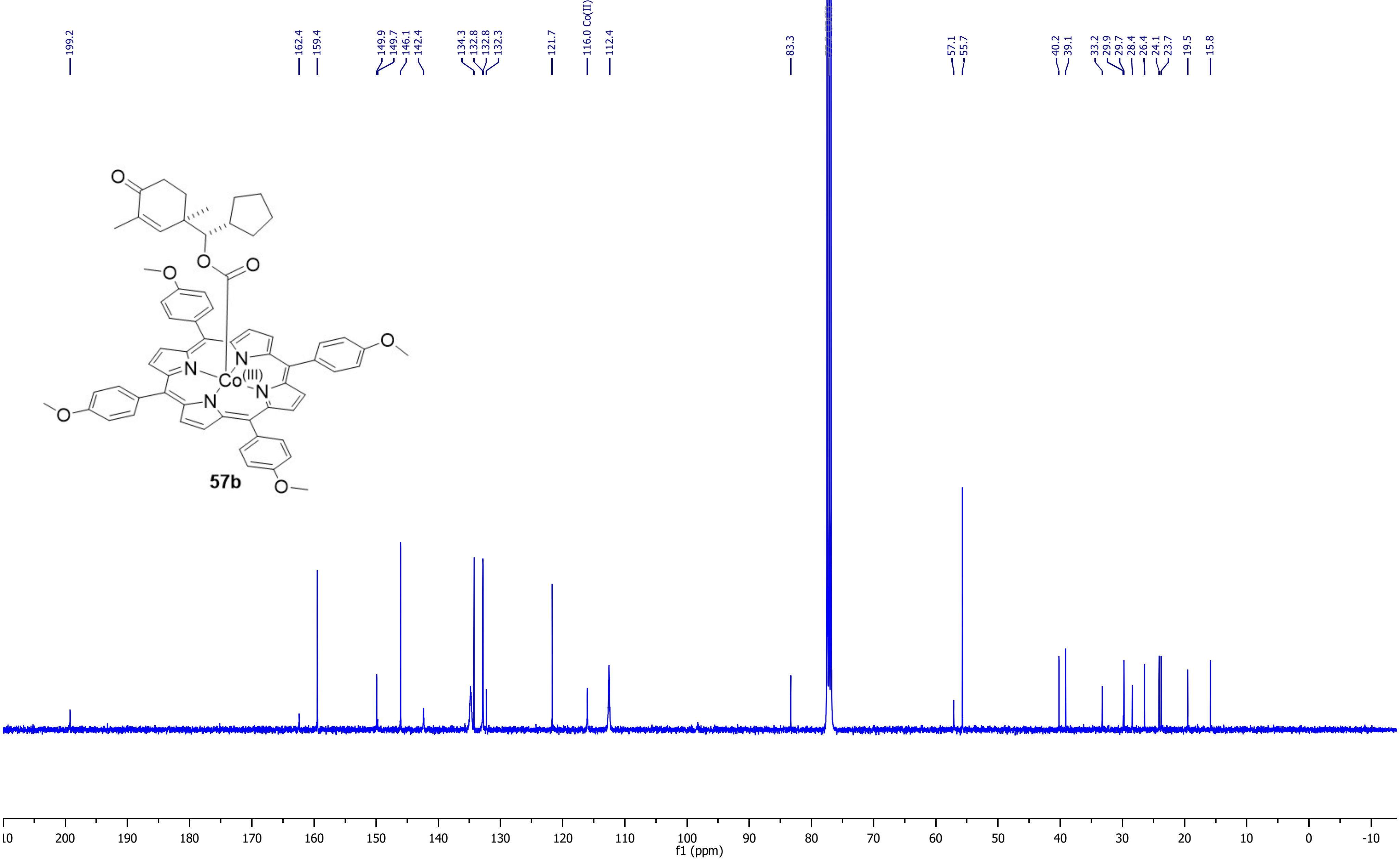

JNH_04_52 13C.1.fid — Cyclopentyl liminoid carbonylation TAP product (0.1033 g)

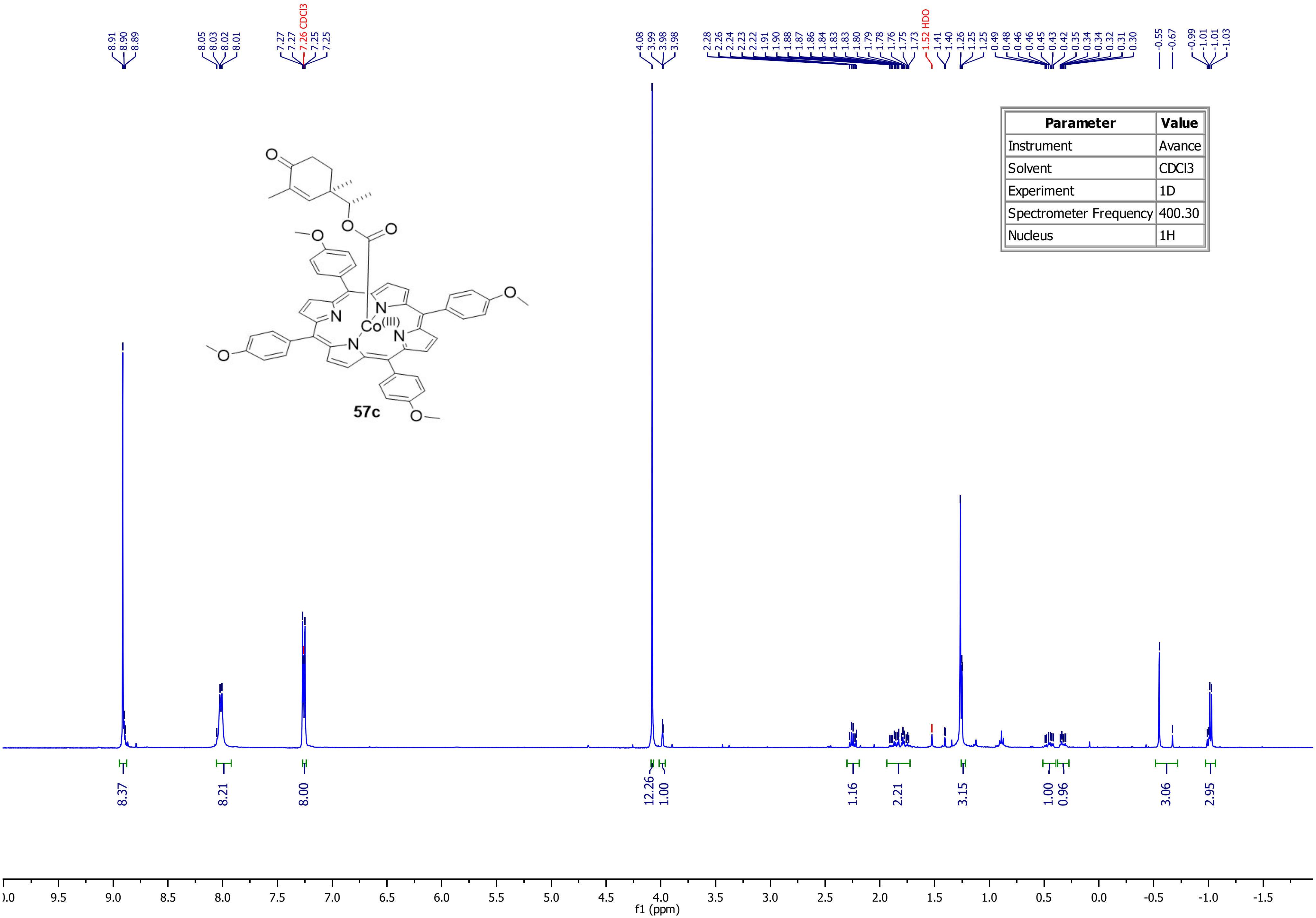

57c

Parameter	Value
Instrument	Avance
Solvent	CDCl3
Experiment	1D
Spectrometer Frequency	400.30
Nucleus	1H

JNH_04_178.3.fid — Methyl Liminoid Carbonylation Co(TAP) Post Prep Plate

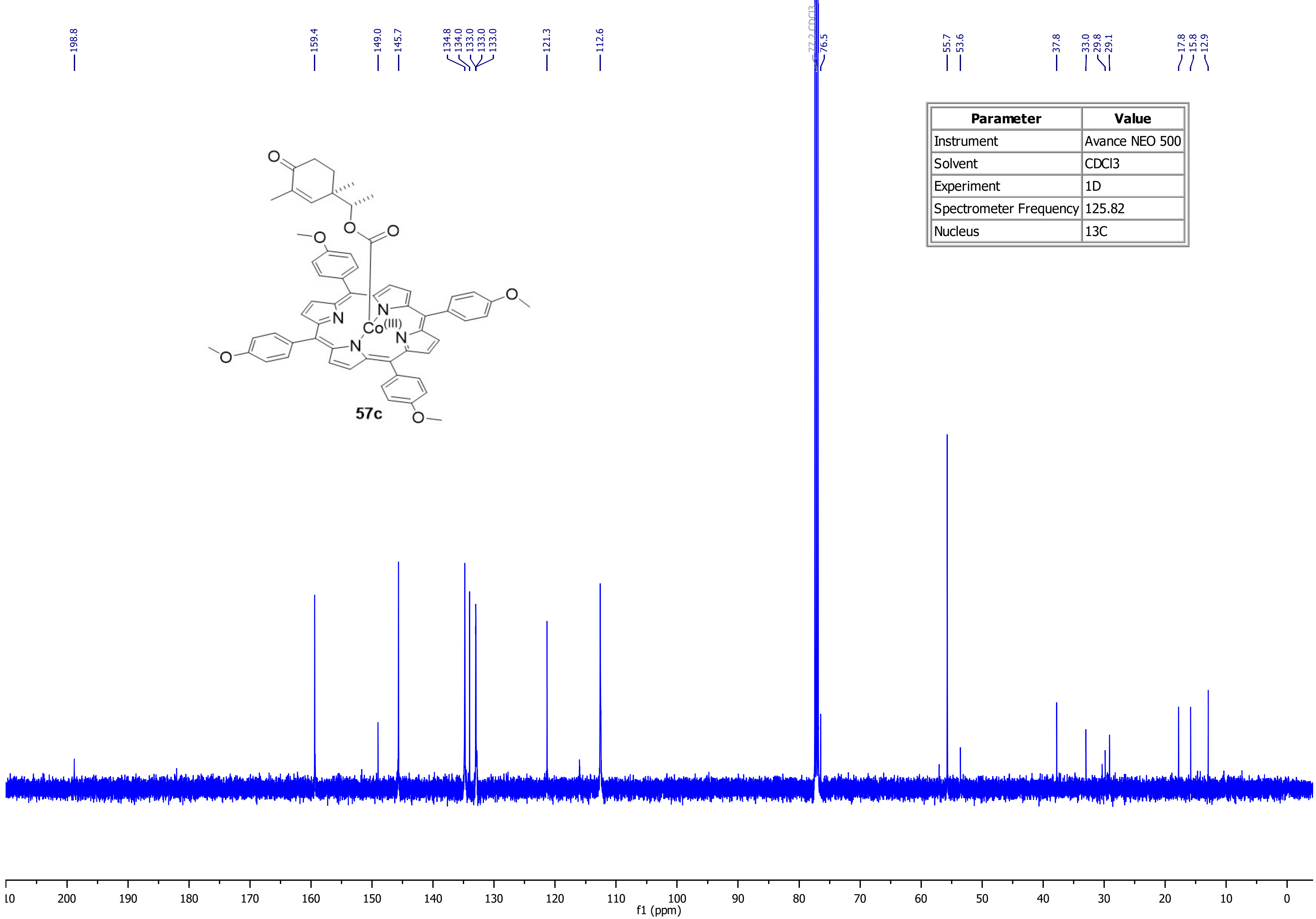

Parameter	Value
Instrument	Avance NEO 500
Solvent	CDCl3
Experiment	1D
Spectrometer Frequency	125.82
Nucleus	13C

57c

JNH_04_178 13C.1.fid — Methyl limonoid Carbonylation and filter over celite 0.2 g (34% yield)

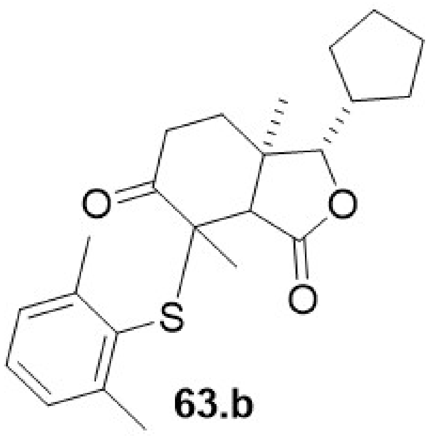

63.b

Parameter	Value
Instrument	Avance NEO 500
Solvent	CDCl3
Experiment	1D
Spectrometer Frequency	500.30
Nucleus	1H

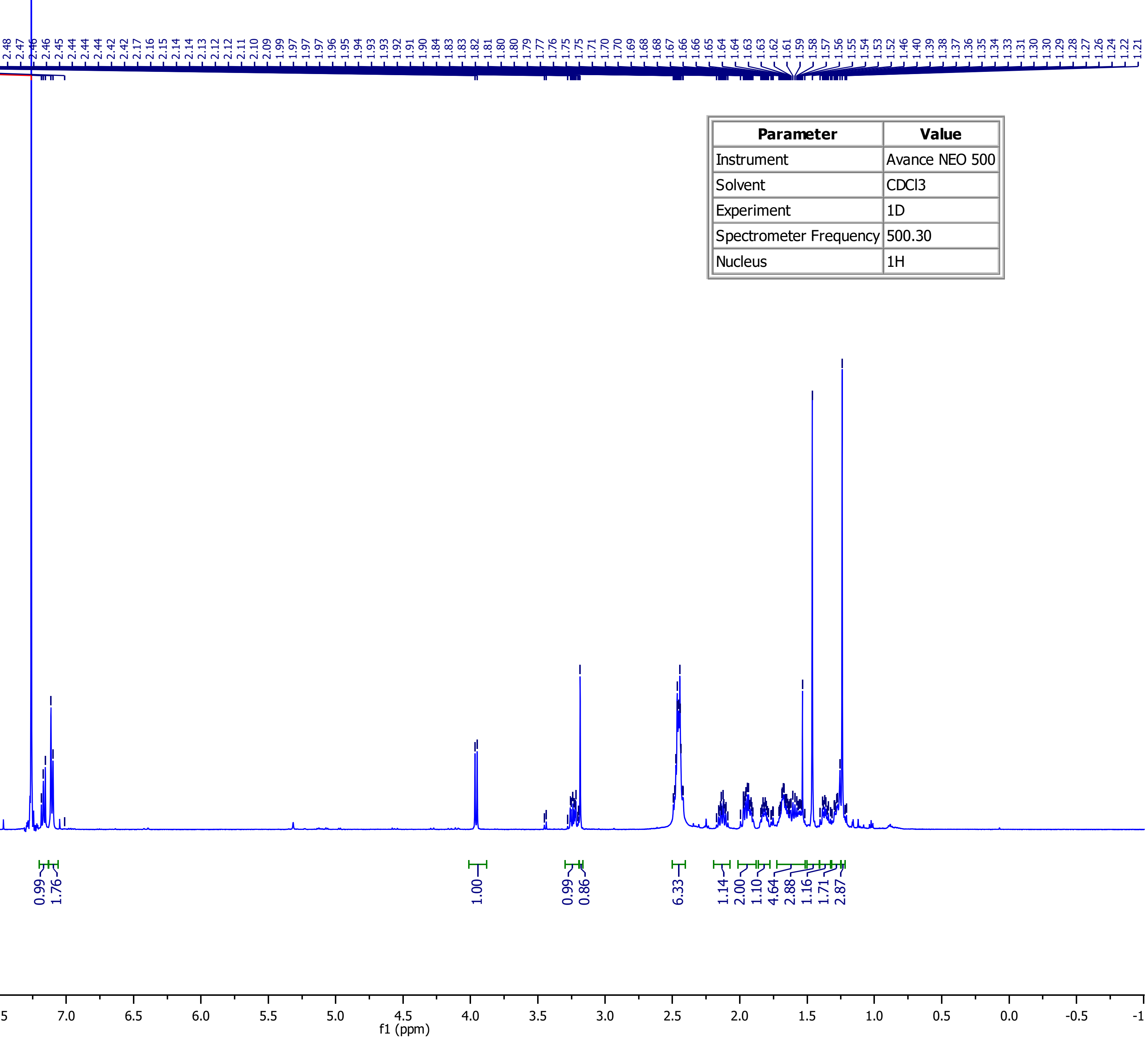

JNH_04_61.2.fid — Fraxinellonone Cyclopentyl model post lactonization frac 6-8 0.0029 g (polar uv active spot)

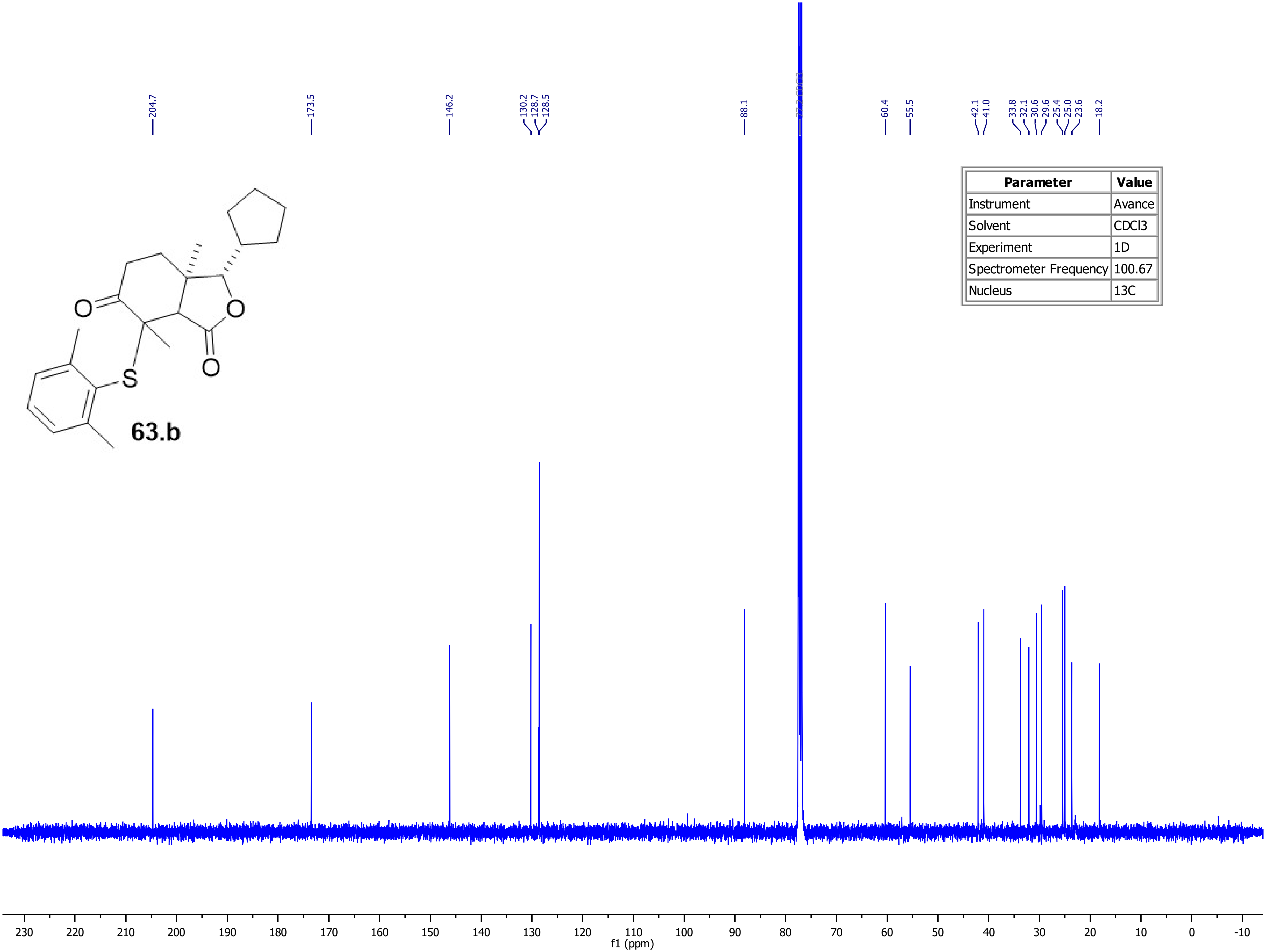

Parameter	Value
Instrument	Avance
Solvent	CDCl3
Experiment	1D
Spectrometer Frequency	100.67
Nucleus	13C

JNH_04_61 13C.2.fid — apparent desired product for cyclopentyl fraxinellonone cyclization

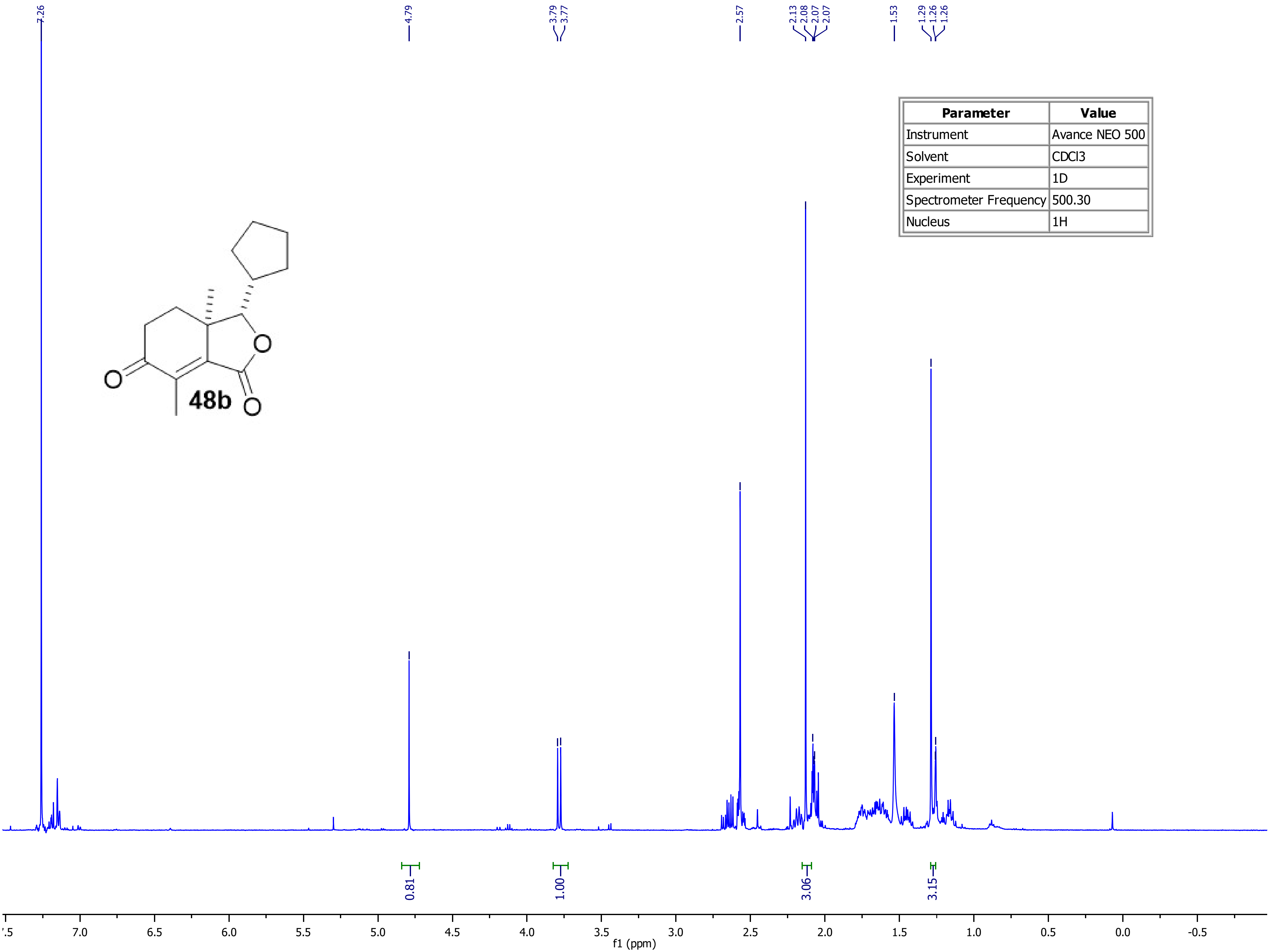

Parameter	Value
Instrument	Avance NEO 500
Solvent	CDCl3
Experiment	1D
Spectrometer Frequency	500.30
Nucleus	1H

JNH_04_61.5.fid — nonpolar top spot after DBU and column

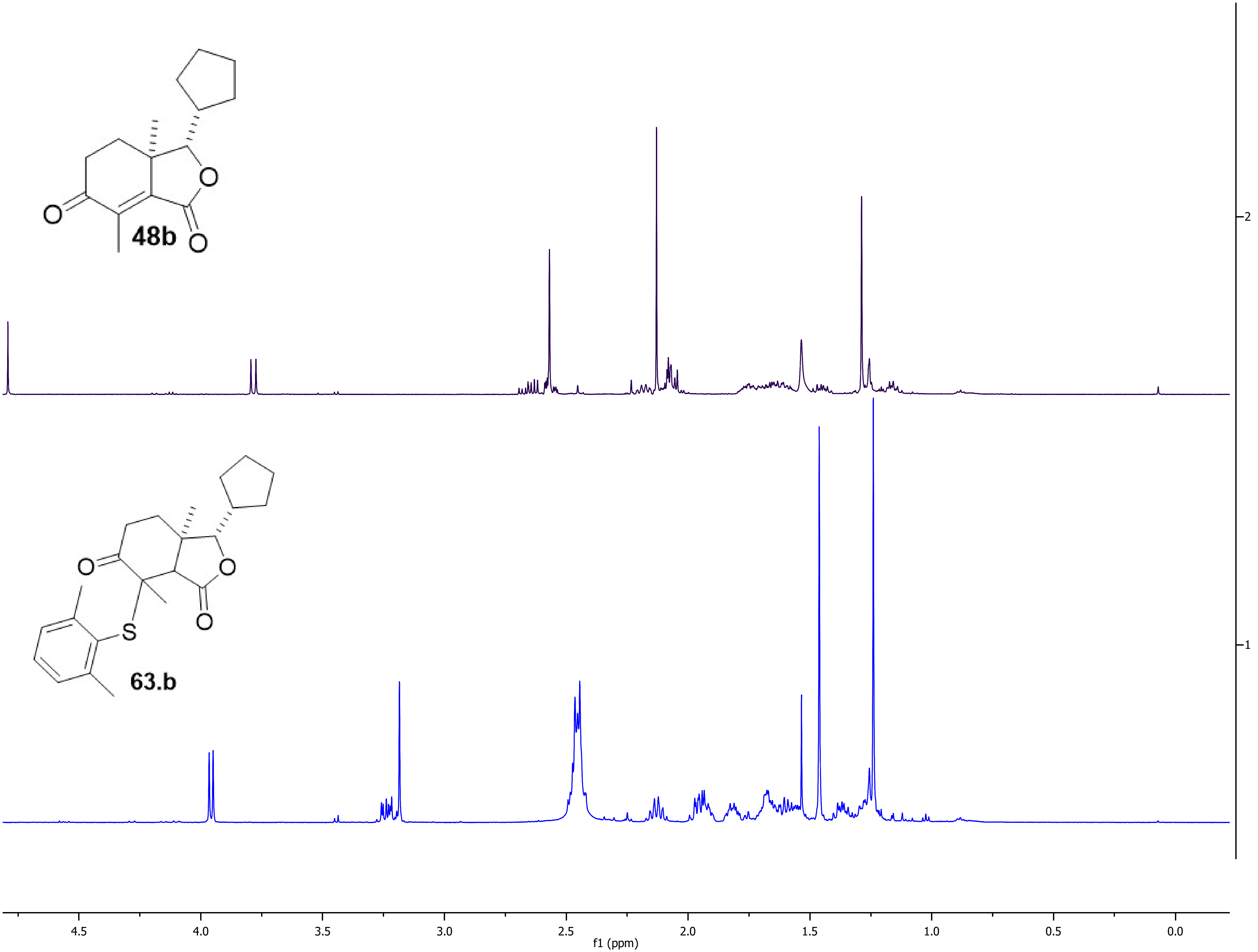

JNH_04_61.5.fid — nonpolar top spot after DBU and column
